# Dynamic Balance: A Thermodynamic Principle for the Emergence of the Golden Ratio in Open Non-Equilibrium Steady States

**DOI:** 10.3390/e27070745

**Published:** 2025-07-11

**Authors:** Alejandro Ruiz

**Affiliations:** Independent Researcher, Sacramento, CA 95814, USA; alejandrothephysicist@gmail.com

**Keywords:** non-equilibrium thermodynamics, entropy, criticality, branching and phyllotaxis, neural avalanches, Fibonacci anyons, rotating turbulence, galactic spirals, golden ratio universality class

## Abstract

We develop a symmetry-based variational theory that shows the coarse-grained balance of work inflow to heat outflow in a driven, dissipative system relaxed to the golden ratio. Two order-2 Möbius transformations—a self-dual flip and a self-similar shift—generate a discrete non-abelian subgroup of $\mathrm{PGL}(2,Q\left(\sqrt{5}\right))$. Requiring any smooth, strictly convex Lyapunov functional to be invariant under both maps enforces a single non-equilibrium fixed point: the golden mean. We confirm this result by (i) a gradient-flow partial-differential equation, (ii) a birth–death Markov chain whose continuum limit is Fokker–Planck, (iii) a Martin–Siggia–Rose field theory, and (iv) exact Ward identities that protect the fixed point against noise. Microscopic kinetics merely set the approach rate; three parameter-free invariants emerge: a 62%:38% split between entropy production and useful power, an RG-invariant diffusion coefficient linking relaxation time and correlation length $D_{\alpha }=\xi^{z}/\tau$, and a $\vartheta =45^{\circ }$ eigen-angle that maps to the golden logarithmic spiral. The same dual symmetry underlies scaling laws in rotating turbulence, plant phyllotaxis, cortical avalanches, quantum critical metals, and even de-Sitter cosmology, providing a falsifiable, unifying principle for pattern formation far from equilibrium.

## 1. Introduction

The golden ratio, φ≈1.618, has been documented in phyllotactic leaf arrangements; branching patterns of trees, blood vessels, lightning, and river deltas [1,2,3,4]; logarithmic spirals in hurricanes and galactic arms [5,6]; power law exponents in rotating turbulence [7,8,9,10]; quasicrystals [11,12,13]; mass gaps of critical Ising chains [14,15]; band structures of twisted bilayer graphene [16,17,18]; Fibonacci anyons dimensionality [19]; avalanche statistics in cortical activity [20,21,22]; and more. Classical equilibrium thermodynamics, tied to isolated systems and static-state variables, cannot explain the ubiquity of spirals, branching, fractals or, scale-invariant kinetics across scales [23,24,25], and a unifying, model-independent, non-equilibrium principle explaining the golden mean universality class is yet to emerge [26,27,28,29,30]. Empirically, these systems share two attributes: (i) they are maintained far from equilibrium by continuous energy or matter influx (e.g., solar radiation, gravitational shear, biochemical energy) [25], and (ii) they exhibit nonlinear, irreversible dissipation (e.g., thermal conduction, radiative cooling, viscous dissipation, chemical enthalpy release) while maintaining coherent large-scale organization and optimal functionality [31].

In this work, we show that a single symmetry-protected variational principle—Dynamic Balance (DB)—forces *any* driven–dissipative system to relax towards the golden ratio. We define a dimensionless ratio–entropy flux field α(t)–comparing two irreducible forms of energy: useful power inflow $\dot{E}$ (reversible) and entropic heat outflow $T\dot{S}$ (irreversible). A large α implies low dissipation; a small α means the system overheats, leaving little energy to build or maintain structure. Most real systems develop negative feedback loops (physiological, hydrodynamic, or electronic) to prevent collapse or runaway behavior, thereby stabilizing their internal state [32]. For instance, excessive anabolism or catabolism harms living organisms, so metabolic circuits self-regulate through hormones and growth-factor inhibition [33]. Consequently, a driven system in a sustained non-equilibrium steady state will adjust α(t) toward a constant, $\alpha^{★}$, that optimally balances useful work against dissipation. Across scales, systems at $\alpha^{★}$ exhibit hierarchical organization—smaller subsystems nested within larger ones—and scale-invariant, fractal dynamics. Therefore, we posit that this optimum is the golden ratio, $\alpha^{★}=\varphi$.

From a group-theoretical perspective, every physical process is the result of a selection rule dictated by an underlying symmetry [34,35]. We show that two discrete Möbius maps $S_{\varphi }$ (a self-dual involution) and $T_{\varphi }$ (a self-similar recursive shift) acting on α, and realized microscopically by antisymmetric Onsager couplings [36,37,38] and cross-correlated noise in active media [39], respectively, generate a non-Abelian subgroup of $\mathrm{PGL}\;\left(2,Q(\sqrt{5})\right)$. Requiring any strictly convex Lyapunov cost R(α) to remain invariant under both maps singles out the unique stable fixed point $\alpha^{★}=\varphi$. Microscopic details affect only transients, and the system *dynamically* self-organizes around the golden mean. This modular symmetry and convex geometry guide the entropy flux field, giving rise to Legendre dual flows and emergent conjugate pairs observed in all physical laws.

$$\mathrm{Symmetry}\;\overset{\mathrm{Modular}\;\mathrm{Duality}}{\to }\;\mathrm{Geometry}\;\overset{\mathrm{Convexity}}{\to }\;\mathrm{Dynamics}\;\overset{\mathrm{Legendre}\;\mathrm{Duality}}{\to }\;\mathrm{Observables}$$

The paper is organized as follows: Section 2 states and proves the core theorem. Section 3 builds the gradient-flow PDE and extracts three parameter-free invariants (entropy split, $\xi^{2}\Gamma$, and $45^{\circ }$ spiral pitch). Section 4 shows that a discrete birth–death Markov model recovers the same continuum PDE limit, while Section 5 embeds the theory in a Martin–Siggia–Rose field integral and derives exact Ward identities that protect φ against noise. We close with cross-domain applications—turbulence, neuroscience, quantum criticality, and cosmology.

## 2. Mathematical Framework

**Theorem 1.**  *Let $S_{\varphi }:\alpha \mapsto \Lambda^{2}/\alpha$ and $T_{\varphi }:\alpha \mapsto 1+1/\alpha \;$ be two Möbius transformations generated microscopically by*

- *$S_{\varphi }:$ antisymmetric Onsager exchange $L_{AB}=-\Lambda^{2}\;L_{BA}$*
- *$T_{\varphi }:$ cross–correlated noise source source $\langle \xi_{A}\xi_{B}\rangle$*

*These two maps generate a discrete, non-Abelian subgroup of $\langle S_{\varphi },T_{\varphi }\rangle \subset \mathrm{PGL}\left(2,Q(\sqrt{5})\right)$. Define the minimum smooth and strictly convex Lyapunov functional C(α)∈(0,∞) such that $C\circ S_{\varphi }=C$, with $C^{\prime \prime }>0$ and C→∞ as $\alpha \to 0^{+},\infty$. Then,*

*(a)*  *Any minimizer $\alpha^{★}$ satisfies $S_{\varphi }\left(\alpha^{★}\right)=T_{\varphi }\left(\alpha^{★}\right)=\alpha^{★}$.*
*(b)*  *Combining the two fixed-point equations gives $\alpha^{★\;2}-\alpha^{★}-1=0$ and $\alpha^{★\;2}=\Lambda^{2}$.*
*(c)*  *Therefore, $\begin{array}{c} \Lambda =\alpha^{★}=\varphi \end{array}$.*

*In the corresponding Martin–Siggia–Rose field theory, the Ward identities*

$$\langle \hat{\alpha }\left(\frac{\Lambda^{2}}{\alpha }-\alpha \right)\rangle =\langle \hat{\alpha }\left(1+\frac{1}{\alpha }-\alpha \right)\rangle =0$$

*forbid stochastic drift. Thus, once Λ is fixed by symmetry, the golden ratio φ becomes a conformal noise-protected attractor: a unique entropic fixed point stabilized by both modular symmetry and convex geometry.*

### 2.1. Coarse-Grained Energy and Entropy Fluxes

Consider an open, driven-dissipative system held in a non-equilibrium steady-state (NESS) by a continuous influx of power and outflux of heat. Let

(1) $$A\left(t\right)=J_{rev}=\dot{E}(t),\;B\left(t\right)=J_{irrev}=T\left(t\right)\;\dot{S}\left(t\right)$$

denote, respectively, the instantaneous work (reversible) flux and the entropic heat (irreversible) flux in the usual system-oriented sign convention (A,B>0). Here, *T* is an *effective* temperature characterizing internal fluctuations, and $\dot{S}$ is the entropy-production rate. Both *A* and *B* are assumed $C^{1}$ functions on [0,∞), and the total throughput is

(2) P(t)=A(t)+B(t)>0.

In a mesoscopic description, these two irreducible fluxes may originate from different blocks of the Onsager matrix or from distinct fields in a Martin–Siggia–Rose path integral, coupled solely through antisymmetric (reactive) exchange and cross-correlated noise [31,38].

### 2.2. Energy–Entropy Flux Ratio

We define a dimensionless ratio describing entropy flow per unit of available energy (or information):

(3) $$\alpha \left(t\right)\;=\;\frac{A(t)}{B(t)}\;=\;\frac{\dot{E}\left(t\right)}{T\left(t\right)\;\dot{S}\left(t\right)},\;\alpha \left(t\right)>0,\;\alpha \in C^{1}\;\left([0,\infty )\right).$$

Neither extreme limit of α is sustainable in a steady-state: $\alpha \to 0^{+}$ corresponds to total dissipation (black hole collapse), while α→∞ implies vanishing entropy export and thermal runaway (wormhole divergence). Real driven systems, therefore, self-tune to an interior fixed value $\alpha^{★}$, which is the focus of the analysis that follows.

### 2.3. Modular Symmetry and Convex Geometry

We define the fundamental symmetry of the entropy field α(x,t) via a modular action that preserves the golden ratio as its unique fixed point. This symmetry is generated by the transformations:

(4) $$T_{\varphi }:\alpha \mapsto 1+\frac{1}{\alpha },\;\;S_{\varphi }:\alpha \mapsto \frac{\varphi^{2}}{\alpha },$$

which together generate a closed, non-Abelian, and discrete subgroup of the projective modular group: $\langle S_{\varphi },T_{\varphi }\rangle \subset \mathrm{PGL}\left(2,Q(\sqrt{5})\right)\subset \mathrm{PGL}\left(2,R^{+}\right)$, whose transformation acts on the entropy field domain α as:

(5) $$\mathrm{PGL}\left(2,Q\left(\sqrt{5}\right)\right)=\left\{\left.\alpha \mapsto \frac{a\alpha +b}{c\alpha +d}\;\right|\;a,b,c,d\in Q(\sqrt{5}),\;ad-bc\neq 0\right\}$$

and satisfies the presentation:

(6) $$\;\langle \;S_{\varphi },\;T_{\varphi }\;|\;S_{\varphi }^{2}={\left(S_{\varphi }T_{\varphi }\right)}^{3}=I\rangle \;.$$

mirroring the modular group relations, but acting on a real positive entropy domain with golden-arithmetic structure. The unique fixed point of this group is the golden ratio $\alpha^{★}=\varphi ,$ which plays the role of a conformal attractor in entropy space. The group $\mathrm{PGL}\left(2,Q(\sqrt{5})\right)$ is richer and more physical than PSL(2,Z), which is discrete, integer, and conformal only in H, and it governs all entropy flows.

We define the unique minimal convex functional invariant under this symmetry:

(7) $$C\left(\alpha \right)=C\left(\frac{\varphi^{2}}{\alpha }\right)=\frac{1}{2}\left(\alpha +\frac{\varphi^{2}}{\alpha }\right),$$

which attains its minimum precisely at $\alpha^{★}=\varphi$. This symmetric functional C(α) plays a Casimir-like role for the modular dynamic system, structurally defining the potential that drives entropy flow. Level sets of C(α) form equipotential surfaces. Gradient descent of C(α) defines entropy flow trajectories, and its derivatives $C^{\prime }(\alpha )$ (gradient), $C^{\prime \prime }\left(\alpha \right)$ (curvature), $C^{\prime \prime \prime }\left(\alpha \right)$ (torsion), …, fully governs the geometric and dynamical structure of the entropy field α(x,t). It defines the gradient flow driving systems toward dynamic balance, the curvature tensor determining local entropy rigidity, and the modular invariance ensuring global recursive symmetry.

The conformal invariance at $\alpha^{★}=\varphi$ implies local scale symmetry, which protects the system from perturbative deformations and endows it with intrinsic resilience to noise and long-range entropy coherence preservation.

### 2.4. Microscopic Origin of the Möbius Involution $S_{\Lambda }$: Onsager Antisymmetric Reactive Exchange

In linear response theory, conjugate pairs of fluxes $J_{i}$ and thermodynamic forces $X_{j}$ obey

(8) $$J_{i}=-\sum_{j\in \{A,B\}}L_{ij}\;X_{j},$$

where $L_{ij}$ is the Onsager matrix [36]. We focus on the purely off-diagonal, entropy-free coupling block: $L_{AA}=L_{BB}=0,\;L_{AB}\neq 0,\;L_{BA}\neq 0,$ and impose antisymmetric reciprocity $L_{AB}=-L_{BA}$, as typical for conservative or reactive couplings (e.g., in Hall transport or chemical oscillators [38,40]. Denoting the output power fluxes by $A\equiv J_{A}$, $B\equiv J_{B}$, we have:

(9) $$\left(\begin{array}{c} A\\ B \end{array}\right)=-\left(\begin{array}{cc} 0 & L_{AB}\\ -L_{BA} & 0 \end{array}\right)\left(\begin{array}{c} X_{A}\\ X_{B} \end{array}\right).$$

Hence, the entropy flux ratio $\alpha =A/B=-L_{AB}X_{B}/L_{BA}X_{A}$ transforms under the exchange of channels $\left(A,X_{A}\right)↔\left(B,X_{B}\right)$ as

(10) $$\alpha^{\prime }\;=\;\frac{B}{A}=\frac{|L_{AB}|}{|L_{BA}|}\;\frac{1}{\alpha }=\frac{\Lambda^{2}}{\alpha },\;\Lambda^{2}\equiv \left|\frac{L_{AB}}{L_{BA}}\right|>0.$$

This is a Möbius transformation of order 2:

(11) $$S_{\Lambda }:\;\alpha \mapsto \frac{\Lambda^{2}}{\alpha },\;S_{\Lambda }=\left(\begin{array}{cc} 0 & \Lambda^{2}\\ 1 & 0 \end{array}\right),\;S_{\Lambda }^{2}=\mathrm{id},$$

The constant Λ quantifies the microscopic asymmetry between the reactive couplings. Its value will later be fixed by requiring modular self-similarity of the dynamics under golden-ratio recurrence.

### 2.5. Microscopic Origin of the Self-Similar Shift $T_{\varphi }$: Cross-Correlated Noise

After establishing that the Möbius flip $S_{\varphi }$ arises from antisymmetric reactive exchange, we now identify the origin of the self-similar modular shift $T_{\varphi }$ in terms of correlated stochastic forcing. In many mesoscopic and active systems, the random forces driving work-like (*A*) and entropy-like (*B*) channels are not statistically independent, but exhibit finite cross-correlation $C=\langle \xi_{A}\;\xi_{B}\rangle$ [38,39]. We model this via a coupled Ornstein–Uhlenbeck process:

(12) $$\dot{A}=-\Gamma_{A}\left(A-A_{0}\right)+kB+\xi_{A}\left(t\right),\;\dot{B}=-\Gamma_{B}\left(B-B_{0}\right)-kA+\xi_{B}(t),$$

with Gaussian white noise correlations,

(13) $$\langle \xi_{i}\left(t\right)\;\xi_{j}\left(t^{\prime }\right)\rangle =2\left(D_{i}\;\delta_{ij}+C\;\left(1-\delta_{ij}\right)\right)\;\delta (t-t^{\prime }),\;i,j\in \{A,B\},$$

where the diagonal elements $D_{A}$ and $D_{B}$ set individual noise intensities (the variance), and $\left|C\right|\leq \sqrt{D_{A}D_{B}}$ quantifies the cross-correlation. The coupling coefficient $k\;=\;L_{AB}\;\left|\partial X_{B}/\partial B\right|$ arises from the same Onsager-antisymmetric exchange responsible for $S_{\varphi }$. Once the physical units of *A* and *B* are rescaled to be commensurate (both interpreted as power fluxes), the conversion factor is absorbed into *k*. The new ingredient is the non-diagonal diffusivity $D_{AB}=C$, encoding the noise-level correlation between the two channels. Solving the Lyapunov equation for the stationary covariance (see SI) yields the steady-state flux ratio:

(14) $$\bar{\alpha }\;\equiv \;\frac{\langle A\rangle }{\langle B\rangle }\;=\;\frac{\Gamma_{B}\;D_{A}-k\;C}{\Gamma_{A}\;D_{B}-k\;C}$$

When C=0, the antisymmetric dynamics reproduce the Möbius flip $\bar{\alpha }\mapsto 1/\bar{\alpha }$, matching the action of $S_{\varphi }$ on the mean state. A non-zero cross-correlation modifies the map by an additive shift proportional to *C*. Expanding (14) to linear order in $C\ll D_{A,B}$, we obtain:

(15) $${\bar{\alpha }}^{\prime }=\frac{1}{\bar{\alpha }}+\frac{C}{D_{B}}+O(C^{2}),$$

showing that tuning $C=D_{B}$ generates an exact unit shift on top of the inversion:

(16) $$T_{\varphi }:\;\alpha \;⟼\;\frac{1}{\alpha }+1,\;T_{\varphi }=\left(\begin{array}{cc} 1 & 1\\ 1 & 0 \end{array}\right),\;\det T_{\varphi }=-1.$$

Unlike the involutive flip $S_{\varphi }$, the shift $T_{\varphi }$ is of infinite order: $T_{\varphi }^{n}\left(\alpha \right)\neq \alpha$ for any n>0. Iterating the combined action of $S_{\varphi }$ and $T_{\varphi }$ yields the continued fraction orbit α,1+1/α, 1+1/(1+1/α), … which converges to the unique positive fixed point φ.

In this formulation, the antisymmetric coupling *k* enforces an instantaneous exchange between entropy and work channels, while the tuned cross-correlation $C=D_{B}$ injects a unit shift in favor of the dissipative component at each swap. Together, they realize the modular transformation $T_{\varphi }$, completing the Möbius generator pair $\left\{S_{\varphi },T_{\varphi }\right\}$ that underpins Dynamic Balance.

Importantly, $D_{B}$ is a measure of intrinsic fluctuations generated by the entropy sector *B*. Matching the cross-correlation to this noise level, $C=D_{B}=\langle \xi_{A}\;\xi_{B}\rangle$, requires no fine-tuning—it naturally arises in open systems where both *A* and *B* couple to a common bath (e.g., phonons coupling electronic and lattice currents, or turbulent eddies affecting both kinetic and thermal flows). In such cases, the cross-covariance self-adjusts to this modularly resonant value.

**Lemma 1.**  *The antisymmetric Onsager coupling $L_{AB}=-L_{BA}$ enforces reversible entropy exchange and generates the modular involution $S_{\varphi }:\alpha \mapsto \varphi^{2}/\alpha$, preserving phase-space symmetry. Irreversibility arises from the recursive entropy shift $T_{\varphi }:\alpha \mapsto 1+1/\alpha$, which breaks time symmetry and drives the system down entropy-curved geodesics toward φ. Thus, the Onsager matrix’s structure maps precisely onto the modular generators: $S_{\varphi }$ reflects conservation; $T_{\varphi }$, dissipation.*

### 2.6. Convex Lyapunov Functional Invariant Under $S_{\varphi }$

To quantify the thermodynamic “distance” of a system from the equilibrium fixed-point, we define a convex Lyapunov functionals R(α), invariant under Möbius involution $S_{\varphi }\;:\alpha \;\mapsto \;\Lambda^{2}/\alpha$. This function serves as a generalized energy landscape: it diverges as $\alpha \to 0^{+},\infty$, and attains its unique global minimum at α=Λ, the fixed point of $S_{\varphi }$:

(17) $$R\left(\alpha \right)\;=\;\frac{4}{\varphi^{2}}\left[C^{2}\left(\alpha \right)-C^{2}\left(\varphi \right)\right]={\left(\frac{\alpha }{\Lambda }-\frac{\Lambda }{\alpha }\right)}^{2},\;R\prime \prime \left(\alpha \right)>0,\;R\left(\alpha \right)\overset{\alpha \to 0^{+},\infty }{\to }+\infty .$$

A complete proof of convexity and invariance is provided in the Supplementary Information. Embedding this scalar cost in a spatial domain $\Omega \subset R^{d}$ defines a free-energy functional:

(18) $$F\left[\alpha \right]=\int_{\Omega }\;\left[\frac{\kappa }{2}\;{\left|\nabla \alpha \right|}^{2}+R\left(\alpha \right)\right]\;d^{d}x,$$

where the diffusivity κ enforces local smoothing of entropy gradients. Taking gradient-descent dynamics (Model-A in the classification of [25]) yields the nonlinear reaction–diffusion equation:

(19) $$\partial_{t}\alpha =-\Gamma \frac{\delta F}{\delta \alpha }=\Gamma \left[\kappa \nabla^{2}\alpha -\partial_{\alpha }R\left(\alpha \right)\right],$$

where Γ>0 sets the relaxation rate. For Neumann or periodic boundary conditions, the energy functional decays monotonically:

(20) $$\dot{F}=\;\int_{\Omega }\frac{\delta F}{\delta \alpha }\partial_{t}\alpha =-\Gamma \;\int_{\Omega }{\left(\frac{\delta F}{\delta \alpha }\right)}^{\;2}\;d^{d}x\leq 0,$$

Thus, every trajectory evolves irreversibly toward the unique global minimizer of R(α), with $\dot{F}\leq 0$ ensuring entropy coherence and φ-stability throughout the dynamics [41].

### 2.7. Common Fixed Point and Identification of Λ=φ

Steady, spatially uniform solutions of the entropy PDE (19) must satisfy $\partial_{\alpha }R\left(\alpha^{★}\right)=0$, i.e., they must lie at extrema of the Lyapunov cost function R(α). Given that *R* is symmetric under the Möbius involution $S_{\varphi }\left(\alpha \right)=\Lambda^{2}/\alpha$, any two points α and $S_{\varphi }\left(\alpha \right)$ lie on the same energy contour: $R(S_{\varphi }\left(\alpha \right))=R\left(\alpha \right)$. For a strictly convex function, distinct points on the same contour cannot both be minima. Hence, if a minimum is fixed under $S_{\varphi }$, the only possibility is that $S_{\varphi }\left(\alpha^{★}\right)=\alpha^{★}$. This condition implies:

(21) $$\alpha^{★2}-\Lambda^{2}=0.$$

Additionally, the modular shift $T_{\varphi }\left(\alpha \right)=1+1/\alpha$ is self-similar and infinite-order. Requiring that $\alpha^{★}$ also be a fixed point of $T_{\varphi }$ yields:

(22) $$\alpha^{★2}-\alpha^{★}-1=0.$$

Solving both equations, the only consistent, positive solution is:

(23) $$\begin{array}{c} \;\Lambda =\alpha^{★}=\varphi \; \end{array}.$$

Thus, the golden ratio emerges non-perturbatively from the interplay of Möbius symmetry and convex geometry. Once these symmetries are imposed, the fixed point $\alpha^{★}=\varphi$ is uniquely selected as the global minimum of any boundary-divergent Lyapunov cost function R(α). The golden attractor arises under the following universal conditions:

(i) Two irreducible power channels A,B forming the entropy flux field α=A/B;
(ii) Möbius inversion symmetry $S_{\varphi }:\alpha \mapsto \varphi^{2}/\alpha$;
(iii) Self-similar translation symmetry $T_{\varphi }:\alpha \mapsto 1+1/\alpha$;
(iv) A strictly convex Casimir functional C(α) diverging at $\alpha \to 0^{+},\infty$.

Hence, convexity of C(α) selects the extremum, and Möbius invariance under the modular subgroup $\langle S_{\varphi },T_{\varphi }\rangle \subset \mathrm{PGL}\left(2,Q\left(\sqrt{5}\right)\right)$ forces that extremum to lie precisely at the golden fixed point $\alpha^{★}=\varphi$. This geometric origin underlies the appearance of Fibonacci sequences, self-similar structures, and golden spirals in driven-dissipative systems.

## 3. Thermodynamic Cost Function and Relaxation Dynamics

In Section 2, we showed that the Möbius involution symmetry $S_{\varphi }$ defines the geometry of the entropy manifold by selecting the φ-invariant Casimir contours, while the minimum of the Casimir C(α) defines the origin of the gradient flow. From this point, all dynamical quantities—including the gradient, curvature, torsion, and evolutionary pathways of the entropy field—follow. We now study the dynamic trade-off between coherent energy flow and irreversible entropy production using our smooth, strictly convex *cost function* $R\left(\alpha \right)\in C^{\infty }$, whose second derivative $\;R^{\prime \prime }\left(\varphi \right)=8/\varphi^{2}>0$ confirms a unique global minimum.

(24) $$R\left(\alpha \right)\;=\;\frac{4}{\varphi^{2}}\left[C^{2}\left(\alpha \right)-C^{2}\left(\varphi \right)\right]{\left(\frac{\alpha }{\varphi }-\frac{\varphi }{\alpha }\right)}^{2},\;\mathrm{with}\;R\left(\alpha \right)\to +\infty \;\mathrm{as}\;\alpha \to 0^{+},\infty .$$

**Figure 1:**
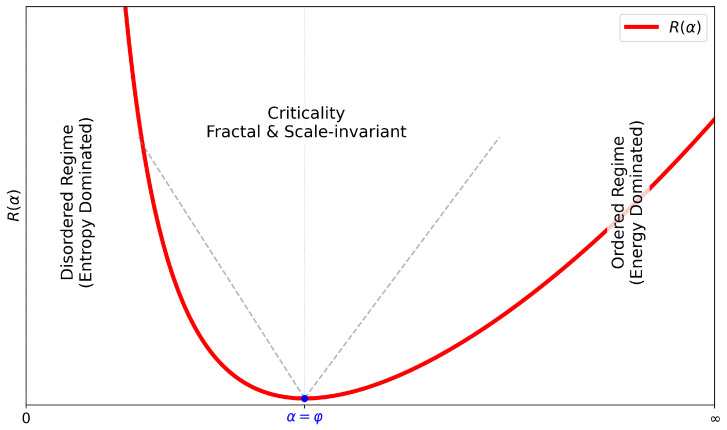
Thermodynamic potential (or cost function) R(α) vs. α. The divergences at $\alpha \to 0^{+},\infty$ represent strongly penalized boundary states. The stable, self-similar critical regime emerges uniquely at the global minimum $\alpha^{★}=\varphi$. Minimizing R(α) does not mean the system is at zero net entropy production. Instead, it means it has found an optimal partition of energy vs. dissipation, optimizing both stability, efficiency and coherence in energy use, and preventing the system from falling into excessive disorder or excessive rigidity.

Physically, this cost penalizes both excessive dissipation (α→0) and excessive energy retention (α→∞), enforcing a Goldilocks balance exactly at the golden ratio (see Figure 1). This non-equilibrium potential drives every initial profile α(x,0)∈(0,∞) monotonically toward the uniform attractor α(x,t)→φ as t→∞.

### Parameter-Free Experimental Invariants

Linearizing the entropy dynamics (19) about $\alpha^{★}=\varphi$, we obtain a local relaxation rate $\mu =R^{\prime \prime }\left(\varphi \right)=8/\varphi^{2}$. This leads to three universal, dimensionless invariants of the entropy flux field: 


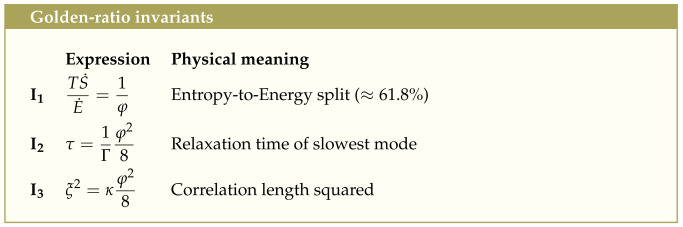


The spatial scale ξ sets the diameter of a coherent patch in which energy and entropy fluxes remain tightly coupled, while τ characterizes the re-equilibration time following a disturbance. Their ratio $\xi^{2}/\tau =\kappa \Gamma$ is a renormalization-group invariant: *If a coherent patch doubles in linear size, its relaxation time quadruples*.

These parameter-free invariants have been observed (within experimental uncertainty) in diverse driven systems, including microbial metabolism and enzyme maintenance, cortical energy balance in active brains, turbulent vortex structures and rotational eddies, vascular branching and phyllotactic lattice development. At the attractor $\alpha^{★}=\varphi$, the energy flux decomposes canonically:

(25) $$\frac{T\dot{S}}{\dot{E}}=\frac{1}{\varphi }\approx 0.618,\;\;\frac{\dot{E}-T\dot{S}}{\dot{E}}=\frac{1}{\varphi^{2}}\approx 0.382.$$

suggesting that in any system where energy is optimally partitioned between reversible work and irreversible fluxes, the characteristic balance is as follows:

- About **61.8% of energy** is thermal entropy ($T\dot{S}$).
- About **38.2% of energy** is effective free energy ($\dot{E}-T\dot{S}$).

This breakdown matches empirical observations across biological and physical systems. In studies of metabolism, growth, and organismal energetics, a ~60–70% dissipation fraction is consistently reported, with the remainder channelled into constructive output [33,42,43,44,45,46,47].

**Example 1**  **(Bathtub Whirlpool Convexity).** *Consider a bathtub that is simultaneously driven by a thin jet of water from the tap (power input $\dot{E}$) and dissipates energy through the drain, where viscous friction converts mechanical energy into heat ($T\dot{S}$). At an intermediate, self-regulated setting, the inflow and outflow balance so that $\alpha^{★}≃\varphi ,$ and the water organizes into a stable, logarithmic vortex with the golden-ratio pitch. The curved bathtub walls play the role of the convex Lyapunov potential R(α), funneling the dynamics toward the single minimum. Thus, the everyday whirlpool illustrates how a bounded, strictly convex “energy landscape” produces a visible, scale-invariant pattern.*

## 4. Discrete Markov Realization of the Flux–Ratio Dynamics

To demonstrate that the continuous Lyapunov dynamics (19) can emerge from an underlying *microscopic* process, we construct a coarse-grained, one0dimensional birth–death Markov chain with *N* discrete states

(26) $$\{\alpha_{i}=i\;\Delta \alpha \;\left|\;i=1,\ldots ,N\right\},\;\Delta \alpha >0,$$

so that $\alpha_{\min}=\Delta \alpha$ and $\alpha_{\max}=N\Delta \alpha$. A threshold index $i_{\mathrm{th}}$ defines an instability cutoff beyond which avalanches (relaxation events) are triggered.

Let $P_{i}\left(t\right)=\mathrm{Pr}\;\left[\alpha \left(t\right)=\alpha_{i}\right]$, and define the probability vector $P\left(t\right)={\left(P_{1},\ldots ,P_{N}\right)}^{\;⊤}$ normalized such that $\sum_{i}P_{i}=1$. The system evolves according to a continuous-time master equation:

(27) $${\dot{P}}_{i}=\sum_{j\neq i}W_{ij}\;P_{j}\;-\;\left(\sum_{j\neq i}W_{ji}\right)P_{i},$$

where $W_{ij}\;\geq \;0$ for i≠j denotes transition rates, and $W_{ii}\;=\;-\sum_{j\neq i}W_{ji}$ ensures conservation of total probability. We now specify two elementary processes:

(i) **Slow drive (energy input):** $\alpha_{i}\;\to \;\alpha_{i+1}$ at constant rate $v_{\alpha }>0$ $\left(W_{\;i+1,i}=v_{\alpha }\right)$.
(ii) **Avalanche relaxation (entropy release):** for any m≥1 and $i>i_{\mathrm{th}}$: $\alpha_{i}\;\to \;\alpha_{i-m}$ at rate $W_{\;i-m,i}=\nu_{m}\;\Theta \left(i-i_{\mathrm{th}}\right)$, where $\nu_{m}$ is the probability per unit time of a downward jump of size *m*. Reflecting boundary imposed $W_{0,i}=W_{N+1,i}=0$.

These rules form a one-dimensional analog of sandpile toppling [48,49]. The generator in (27) is irreducible, ensuring the existence of a unique stationary distribution $P^{\left(\infty \right)}$ exists. For $i\geq i_{\mathrm{th}}$, the steady-state satisfies

(28) $$\frac{P_{i+1}^{\left(\infty \right)}}{P_{i}^{\left(\infty \right)}}=\frac{v_{\alpha }}{\nu }\;,\;\nu :=\sum_{m\geq 1}\nu_{m},$$

This leads to a geometric tail above threshold and a unimodal profile peaking at $i^{★}≃i_{\mathrm{th}}+\nu /v_{\alpha }$, which, in the continuum limit, converges to $\alpha^{★}=\varphi$.

### 4.1. Continuum Limit and Fokker–Planck Correspondence

Letting α=iΔα and defining the probability density $P\left(\alpha ,t\right)=P_{i}\left(t\right)/\Delta \alpha$, we expand (27) to second order in Δα≪1 using the Kramers–Moyal expansion [50]:

(29) $$\partial_{t}P=-\partial_{\alpha }\;\left[v_{\alpha }\;P\right]+\partial_{\alpha }^{2}\;\left[D_{\alpha }\;P\right]+O\;\left({\left(\Delta \alpha \right)}^{3}\right),$$

with drift $v_{\alpha }$ and diffusion coefficient $D_{\alpha }=\frac{1}{2}\;\Delta \alpha \;\sum_{m}m^{2}\nu_{m}$.

Comparing with the DB field equation (19) gives the dictionary:

(30) $$v_{\alpha }=\Gamma ,\;D_{\alpha }=\Gamma \;\kappa .$$

As Δα→0, all higher-order Kramers–Moyal terms vanish, and the Markov chain converges exactly to the deterministic Lyapunov flow. For single-step avalanches $\nu_{m}=\nu_{1}\delta_{m1}$ and $v_{\alpha }=\nu_{1}$, the stationary distribution becomes a discrete Gaussian peaked at $i^{★}≃\varphi /\Delta \alpha$. In this regime, the avalanche-size distribution follows $P\left(S\right)\propto S^{-3/2}$, matching the mean-field exponent of sandpile models and the scaling observed in critical neural and condensed matter systems [22,48,51,52,53].

### 4.2. Parameter Dictionary


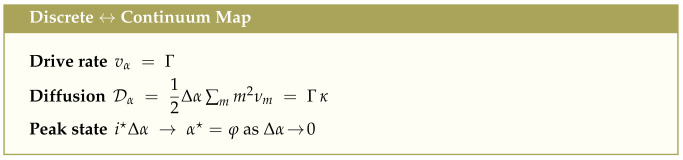


Here, κ controls the spatial propagation of entropy imbalance—interpretable as thermal conductivity, stiffness, or axonal spread—while Γ sets the local rate of relaxation, e.g., via viscosity, phonon damping, or synaptic recovery.

**Example 2**  **(Sandpile avalanche criticality).** *In the classical Abelian sandpile model, a slow “rain” of grains, added at rate $v_{\alpha }$, builds a heap until the local slope at some site exceeds a threshold height $i_{\mathrm{th}}$. The site then topples, redistributing one grain to each neighbor; the relaxation may propagate and produce an avalanche whose size S (total topplings) obeys the mean-field law. Our birth–death chain is the direct energy-flux analogue. Slow drive adds a grain of “usable power” Δα to the system, mirroring the external input that steepens the pile. Once α crosses the instability line $i_{\mathrm{th}}$, a stochastic event of size m=1,2,… transfers mΔα from the work channel A into the dissipation channel B. This implements the toppling rule in energy–entropy space. Because the drive $v_{\alpha }$ and the avalanche kernel $\nu_{m}$ are held fixed while the accessible state space extends to arbitrarily large i, the Markov process self-organizes to a stationary distribution peaked at $\alpha^{★}≃\varphi$. The continuum limit reproduces the gradient-flow PDE (19), and the avalanche–size vstatistics approaches the same power law, as in the canonical sandpile SOC [22,48,53].*

## 5. Modular Symmetry and Non-Equilibrium Field Theory

Having shown in Section 2 and Section 3 that the dual Möbius symmetry uniquely selects Λ=φ, and in Section 4 that the discrete Markov chain recovers the continuous Lyapunov flow as Δα→0, we now extend the theory to include fluctuations. We demonstrate that the golden-ratio attractor remains symmetry-protected in the stochastic setting, and derive exact selection rules and Ward identities [54,55,56,57].

### 5.1. Stochastic Dynamic-Balance Equation

We promote the entropy field α≡α(x,t) to a stochastic variable over a *d* spatial dimensional spatial domain, governed by the Langevin equation:

(31) $$\partial_{t}\alpha =\Gamma \left[\kappa \nabla^{2}\alpha -\partial_{\alpha }R\left(\alpha \right)\right]+\eta ,\;\langle \eta \left(x,t\right)\eta \left(x’,t’\right)\rangle =2D,\delta^{d}(x-x’),\delta \left(t-t’\right).$$

Here, η is Gaussian white noise of strength *D*, and Γ>0 is the relaxation rate. Importantly, *D* and Γ are independent parameters: the system is driven and does not obey detailed balance. The relation $D_{\alpha }=\kappa \Gamma$ seen in the Fokker–Planck limit is not a fluctuation–dissipation theorem, but an RG-fixed constraint imposed by Möbius symmetry after coarse-graining. Setting D=0 recovers pure Lyapunov descent toward the golden attractor $\alpha^{★}=\varphi$.

### 5.2. MSRJD Path Integral Formalism

To study the statistical field theory, we follow the Martin–Siggia–Rose–Janssen–de Dominicis (MSRJD) formalism [58], introducing a response field $\hat{\alpha }\left(x,t\right)$ and writing the generating functional:

(32) $$Z\left[J,\hat{J}\right]=\int D\alpha \;D\hat{\alpha }\;\exp\;\left(-S\left[\alpha ,\hat{\alpha }\right]+\int d^{d}x\;dt\;\left[\;J\;\alpha +\hat{J}\;\hat{\alpha }\;\right]\right).$$

with the stochastic action:

(33) $$S\left[\alpha ,\hat{\alpha }\right]=\int \;d^{d}x\;dt\;\left\{\hat{\alpha }\left[\partial_{t}\alpha -\Gamma \left(\kappa \nabla^{2}\alpha -\partial_{\alpha }R\left(\alpha \right)\right)\right]\;+\;D\;{\hat{\alpha }}^{2}\right\}.$$

Correlation functions of α and $\hat{\alpha }$ follow from functional derivatives with respect to $J,\hat{J}$. We adopt the Itô convention: the Jacobian from $\delta [\partial_{t}\alpha -\ldots ]$ is a constant and set to unity.

### 5.3. Modular Symmetries and Ward Identities

Only involutive Möbius transformations commute with the stochastic time-reversal symmetry $\left(t,\alpha ,\hat{\alpha }\right)\to \left(-t,\alpha ,-\hat{\alpha }\right)$, leaving the action invariant. Higher-order modular elements break this symmetry by flipping the sign of the MSR term and thus cannot survive in the stochastic theory [54,57,58,59].

We embed both Möbius maps as exact symmetries of $S[\alpha ,\hat{\alpha }]$, with transformations chosen to preserve the path integral measure ($D\alpha \;D\hat{\alpha }$):

**Self-dual flip $S_{\varphi }:\;\alpha \mapsto \frac{\varphi^{2}}{\alpha }:$**

(34a) $$\begin{array}{c} \delta \alpha =\varepsilon \left(\frac{\varphi^{2}}{\alpha }-\alpha \right),\;\delta \hat{\alpha }=-\varepsilon \left(\frac{\varphi^{2}}{\alpha^{2}}+1\right)\hat{\alpha }, \end{array}$$

**Self-similar shift $T_{\varphi }:\;\alpha \mapsto 1+\frac{1}{\alpha }:$**

(34b) $$\begin{array}{c} \delta \alpha =\varepsilon \left(1+\frac{1}{\alpha }-\alpha \right),\;\delta \hat{\alpha }=-\varepsilon \left(\frac{1}{\alpha^{2}}-1\right)\hat{\alpha }, \end{array}$$

By direct substitution, we confirm that $S\left[\alpha +\delta \alpha ,\hat{\alpha }+\delta \hat{\alpha }\right]=S\left[\alpha ,\hat{\alpha }\right]$. Applying these variations to the path-integral and requiring δZ=0 yields exact Ward identities for any operator $O[\alpha ,\hat{\alpha }]$ [56].

**$S_{\varphi }$–Ward identity:**

(35a) $$\begin{array}{c} \int d^{d}x\;dt\;\langle \hat{\alpha }\left(x,t\right)\;\left(\frac{\varphi^{2}}{\alpha (x,t)}-\alpha \left(x,t\right)\right)\;O\rangle =0. \end{array}$$

**$T_{\varphi }$–Ward identity:**

(35b) $$\begin{array}{c} \int d^{d}x\;dt\;\langle \hat{\alpha }\left(x,t\right)\;\left(1+\frac{1}{\alpha (x,t)}-\alpha \left(x,t\right)\right)\;O\rangle =0. \end{array}$$

**Theorem 2.**  *Let $O_{m}\left(\alpha \right)\propto \alpha^{m}$ carry modular charge m under $S_{\varphi }$ or $T_{\varphi }$. Then for any nonzero n-point correlator,*

(36) $$\langle O_{m_{1}}\;\ldots \;O_{m_{n}}\rangle \neq 0\;⟹\;\sum_{i=1}^{n}m_{i}=0.$$

Choosing O=1 shows fluctuations alone cannot bias α away from φ: the golden attractor is symmetry-protected. Any process violating modular charge conservation is strictly forbidden. Thus, the same Möbius generators that extremize the Lyapunov functional survive in the stochastic theory and protect the attractor through exact Ward identities [57,58,59]. This parallels how conformal symmetry and *S*–duality constrain correlators in equilibrium quantum field theory (QFT) [60,61]. In the non-equilibrium framework, the analogous role is played by the discrete modular symmetries of entropy fluxes dynamics.

**Example 3.**  *Let us evaluate the two Ward identities (35a) and (35b) using the insertion $O\left[\alpha \right]=\alpha^{n}$, $n\in N_{0}$. Substituting into the identities and integrating by parts yields the following exact constraints:*

(37) $$\left(\varphi^{2}-\langle \alpha^{2}\rangle \right)\;\langle \alpha^{n-1}\rangle =0,\;\left(1+\langle \alpha^{-1}\rangle -\langle \alpha \rangle \right)\;\langle \alpha^{\;n}\rangle =0.$$

*For n=0, the first identity enforces $\langle \alpha^{2}\rangle =\varphi^{2}$; for n=1, the second identity gives 〈α〉=φ. Since these constraints hold simultaneously for all $n\in N_{0}$, they imply by recursion $\langle \alpha^{m}\rangle =\varphi^{m}$ for every m∈N,∀m∈N, i.e., all moments are locked to powers of the golden ratio.*

*In particular, the one-point function satisfies 〈α〉=φ exactly, to all orders in perturbative theory. This result illustrates how the golden fixed point is not just an attractor of deterministic dynamics but is symmetry-protected against all Gaussian (and weakly non-Gaussian) noise, due to modular invariance embedded in the stochastic field theory.*

### 5.4. Quadratic Theory and Diffusive Pole

To analyze fluctuations around the golden attractor, we expand the entropy field near $\alpha^{★}=\varphi$ as:

(38) α(x,t)=φ+δα(x,t),|δα|≪1,

Expanding the MSRJD action to quadratic order in δα and $\hat{\alpha }$, we obtain:

(39) $$S_{2}\;=\;\int d^{d}x\;dt\;\left\{\hat{\alpha }\left[\partial_{t}-\Gamma \left(\kappa \nabla^{2}-\mu \right)\right]\delta \alpha \;+\;D\;{\hat{\alpha }}^{2}\right\},\;\mu =R^{\prime \prime }\left(\varphi \right)=\frac{8}{\varphi^{2}}.$$

The retarded Green’s function, defined as the response to a perturbation at $\left(x^{\prime },t^{\prime }\right)$, is governed by the inverse of the operator in the linear term (neglecting the ${\hat{\alpha }}^{2}$ term which contributes only to 〈αα〉, not to $G_{R}$):

(40) $$G_{R}\left(\omega ,q\right)=\frac{1}{\;i\omega +\Gamma \left({\kappa |q|}^{2}+\mu \right)\;}\;.$$

This propagator has a single diffusive pole located at:

(41) $$\omega^{★}=-i\;\Gamma \left(\kappa q^{2}+\mu \right).$$

At long wavelengths (q→0), the dispersion relation reduces to ω∼−iΓμ, giving the familiar diffusive scaling $\omega ∼-iq^{2}$, with dynamical exponent z=2. To geometrize the fluctuation modes, consider the argument of the complex frequency $\omega^{★}$. Define:

(42) $$\vartheta \left(q\right)=\arg\left(i\omega^{★}\right)=\tan^{-1}\left(\frac{\mu }{\kappa q^{2}}\right).$$

which interpolates from a purely relaxational mode q→0,ϑ→π/2 at long wavelengths (IR limit) to a purely diffusive mode q→∞,ϑ→0 at short wavelengths (UV limit). At the crossover scale $q_{c}=\sqrt{\mu /\kappa }$, we find $\omega^{★}=-i\;\Gamma \mu \;\left(1+i\right)$, so that $\vartheta (q_{c})=\pi /4$. This $45^{\circ }$ phase angle represents perfect balance between real and imaginary parts—between reversible energy and irreversible dissipation. In control theory, this is the critical damping angle. In real space, it corresponds to a logarithmic spiral with constant pitch:

(43) $$r\left(\theta \right)=a\;e^{b\;\theta },\;b=\frac{ℜ\;\omega^{★}}{ℑ\;\omega^{★}}=1,$$

or equivalently, $\;r\left(\theta \right)=a\;\varphi^{\;\theta /\pi }$, using the identity $e^{\pi }=\varphi^{\pi /\ln\varphi }$. Thus, the pole’s damping ratio directly maps onto the golden spiral—a physical manifestation of modular balance in space–time.


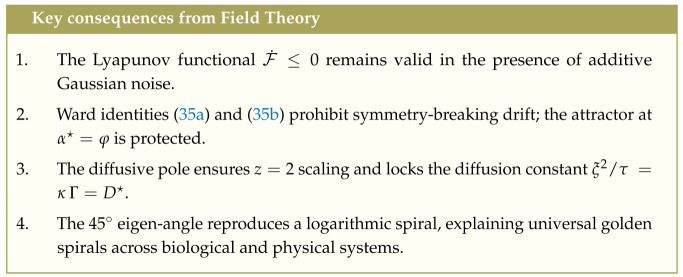


#### Interpretation of $D^{★}=\kappa \Gamma$: A Modular Einstein Relation

The effective macroscopic diffusion constant for fluctuations in the entropy field α(x,t) is:

(44) $$D^{★}=\kappa \Gamma .$$

where κ measures spatial coupling, or how strongly neighboring regions equilibrate α. Γ is the local response speed, dictating how fast α relaxes back to φ.

Under coarse-graining—$\kappa \;∼\;b^{-2},\;\Gamma \;∼\;b^{+2}$—so their product remains scale-invariant. This mirrors the Einstein relation $D=\mu k_{B}T$, where mobility and temperature compensate. Here, modular symmetry replaces equilibrium as the principle that constrains the fluctuation–dissipation balance.

### 5.5. Dynamical-Exponent Landscape

At Gaussian (tree-level) approximation, the dynamic exponent is z=2 (see Appendix B). However, beyond mean-field, nonlinearities introduce loop corrections. In particular, the one-loop vertex from the cubic term $∼{\left(\partial_{\alpha }R\right)}^{2}\;\hat{\alpha }\;\delta \alpha^{2}$ renormalizes the quadratic propagator and alters the effective dispersion relation. Importantly, modular symmetry structure determines the form and strength of these corrections:

- **Full modular symmetry** ($S_{\varphi }$ *and* $T_{\varphi }$ preserved; Onsager matrix antisymmetric):
  The nonlinearity couples left- and right-moving modes through dual Möbius rotations. The retarded self-energy inherits the golden eigen-angle, and the dispersion becomes $\omega ∼q^{\varphi }$, i.e., z=φ. This is the fully symmetry-protected golden dynamic exponent, characteristic of balanced, φ-stabilized flows.
- **Self-dual line** ($S_{\varphi }$ preserved, $T_{\varphi }$ broken): One modular charge is violated. The resulting loop integral resembles the modified KPZ class with $z=\sqrt{2}$ [62]. This phase retains φ-inversion symmetry but lacks self-similarity, leading to intermediate roughening.
- **No modular symmetry** (Onsager symmetric): Standard KPZ-type scaling emerges, depending on conservation laws z∈{1,3/2,2}, spanning ballistic, superdiffusive, and diffusive behavior [57,63].
- **Strong disorder/broken detailed balance** (e.g., random-field landscape, Sinai-type potential): The system becomes glassy and subdiffusive. Scaling slows to z=1/2, typical of Sinai creep in 1D random environments (disorder induced irreversibility) [64,65].

**Theorem 3.**  *The golden point $\alpha^{★}=\varphi$ is the unique real fixed point of the Möbius transformation T∘S∈PSL(2,Z). This transformation has order 3, satisfying ${\left(TS\right)}^{3}=I$ geometrically classifying φ as a real order-3 hyperbolic fixed-point. It is the projection to $R_{+}$ of the complex elliptic fixed point $\tau =e^{2\pi i/3}\in H$, the triangle vertex of the modular tiling with internal angle π/3. At this point, the full modular symmetry PSL(2,Z) acts conformally on the entropy balance field $\alpha \in R_{+}$, both generators $S_{\alpha }$ and $T_{\alpha }$ are preserved, the entropy Casimir $C\left(\alpha \right)=\frac{1}{2}\left(\alpha +\varphi^{2}/\alpha \right)$ is minimized, the RG-invariant diffusivity $D_{z}=\xi^{z}/\tau$ attains optimal scaling with z=φ. Therefore, $\alpha^{★}=\varphi$ defines the unique conformal and modular RG fixed point on $R_{+}$, stabilized by Möbius symmetry.*

This dynamical-exponent landscape provides a symmetry-based RG classification of entropy field dynamics. Each value of $z_{n}=2\cos\left(\pi /n\right)$ corresponds to a distinct Coxeter–modular class, encoding a specific pattern of symmetry breaking, Onsager structure, and renormalization flow. The dynamic exponent governs the anisotropic scaling between space and time: $t∼\xi^{z_{n}}$, setting the causal geometry of entropy transport. This classification is intrinsically tied to geodesic structure in modular and hyperbolic spaces. Each Coxeter class $I_{2}\left(n\right)$ gives rise to a set of closed geodesics with angular period $\frac{2\pi }{n}$. The dynamical exponent $z_{n}$ encodes the geodesic curvature of the entropy flow in modular space.

The golden ratio exponent z=φ marks a universality class uniquely protected by full Möbius symmetry—where both modular generators $S_{\alpha }$ and $T_{\alpha }$ are preserved. At this fixed point, entropy flux is optimally balanced between reversible and irreversible components, minimizing the golden Casimir and maximizing the RG-invariant diffusivity $D_{\varphi }=\xi^{\varphi }/\tau$.

This structure reveals that dynamical exponents are not just empirical parameters, but modular symmetry charges—labels of how entropy flow transforms under Coxeter–Möbius RG symmetries. The golden point z=φ represents the only uncharged (invariant) fixed point, where entropy flow is modularly neutral, and balance is perfectly preserved. At this point, the entropy field is invariant under both $S_{\alpha }$ and $T_{\alpha }$, and the full group $\mathrm{PGL}(2,Q\left(\sqrt{5}\right))$ acts as if it were locally conformal, stabilizing the entropy flow and minimizing the golden Casimir. Thus, conformal invariance emerges only at the golden point—and it is not generic, but modularly protected.

## 6. Discussion

The symmetric Möbius involution $\alpha \mapsto \varphi^{2}/\alpha$ encodes a modular duality intrinsic to the entropy field, mapping energy–entropy configurations into their golden-conjugate counterparts. The fixed point $\alpha^{★}=\varphi$ uniquely minimizes the convex Casimir potential C(α), defining the universal entropy balance point. This potential generates the intrinsic geometric landscape for entropy flow along geodesics in this landscape $H/\Gamma_{0}\left(N\right)$, constrained by Coxeter symmetry class $I_{2}\left(n\right)$, each dictating a distinct dynamic exponent $z_{n}$ and flow curvature. Conjugate pairs ($\alpha ,\varphi^{2}/\alpha$) form modular dual observables—mirrored entropy states under the Möbius involution—that encode the field’s reversible flow structure. φ governs the flow of entropy through modular spacetime. Microscopic details set only the approach rate (Γ, Section 3) and spatial coupling (κ, Section 4); all *dimensionless* observables are fixed by symmetry.

(45) $$\left\{1/\varphi :1/\varphi^{2},\;D=\kappa \Gamma =\xi^{z}/\tau ,\;\vartheta =45^{\circ }\right\}$$

**Thermodynamic partition:** At $\alpha^{★}=\varphi$, 1/φ≃61.8% of inflowing power is dumped as heat; the remaining $1/\varphi^{2}≃38.2\%$ fuels coherent structure. Measured maintenance-vs-growth splits in microbes, plants, animals, and cortical tissue cluster near this ratio [42,66].

**Renormalisation-group invariant:** The diffusivity $D=\xi^{z}/\tau \;=\;\kappa \;\Gamma$ remains fixed under coarse graining, persisting across scales because the geometric structure is modularly renormalization-invariant: changes in length or time do not break the φ-balance. In the diffusive limit, doubling the linear size of a coherent patch, therefore, quadruples its relaxation time—exactly as the Einstein relation $D=\mu k_{B}T$ couples diffusivity and mobility in equilibrium [67,68].

**Emergent geometry:** Linear response yields a $45^{\circ }$ eigen-angle in the complex frequency plane, mapping to the golden-pitch logarithmic spiral in real space $\tan\psi =1/\varphi \;(\psi ≃31.7^{{}^{\circ}})$. This explains why vortices in rotating turbulence, hurricanes, galactic arms, and phyllotactic patterns share the same spiral pitch [5,6,7,9,69,70,71,72,73].

**Dynamic-exponent landscape:** Beyond mean field, loop corrections shift the exponent *z*: Full modular symmetry ($S_{\varphi }$ + $T_{\varphi }$ and antisymmetric Onsager matrix) produces z=φ; breaking $T_{\varphi }$ alone gives $z=\sqrt{2}$; and removing both yields the KPZ/Levy line, z∈{1,3/2,2} (Table 1).

A recurrent question is how the *three* diffusion constants that appear in the paper—the microscopic channel variances $D_{A,B}$ in Section 2, the lattice diffusion $D_{\alpha }$ of the birth–death chain in Section 4, and the stochastic amplitude $D^{★}$ in the MSR action Section 5—are related. The link is the *matched–bath condition* $C=\langle \xi_{A}\;\xi_{B}\rangle =D_{B}$ between the cross-covariance *C* to the entropy-sector variance $D_{B}$, which ensures that every dissipative kick is transmitted to the work channel with unit efficiency. Under this condition, the slow *balance* field inherits a *single* effective noise level D=κΓ, where κ is the stiffness that spreads deviations of α and Γ is the local relaxation rate. Coarse-graining the birth–death chain reproduces the same value, $D_{\alpha }\to \kappa \Gamma$, and the continuum MSR functional keeps it unchanged. Thus the Einstein-like product κΓ survives intact from the microscopic Ornstein–Uhlenbeck description to the macroscopic field theory, providing an internally consistent “noise ladder” that underlies the golden-ratio universality class [74,75,76]. Rather than tuning temperature or pressure to a critical point $\left(g-g_{c}\right)$, the system self-organizes its energy–entropy flux gradient to the golden fixed point (α−φ). From biochemical chirality to turbulent galaxies, from neural avalanches to non-Fermi-liquid metals, the same Möbius duality and recursive modular geometry encode how entropy organizes structure across scales. Each φ-deviation acts as a local “entropy curvature”, and its relaxation under C(α) guides systems back toward balance. This self-organized modularity manifests as fractals, scaling laws, and golden-pitch spirals.

$$\left(D_{A},D_{B}\right)\;\overset{\;\mathrm{coarse}\text{-}\mathrm{grain}\;}{\to }\;D_{\alpha }\;\overset{\;\mathrm{Kramers}–\mathrm{Moyal}\;}{\to }\;D=\kappa \Gamma .$$

### 6.1. Two-Fluid Decomposition and Quantum-Critical Universality

The order-2 modular involution $S_{\varphi }$ mandates a fundamental dichotomy in any coarse-grained entropy flux: a component that *recirculates* as coherent structure (channel *A*) and a component that *dissipates* as heat (channel *B*). This partition is enforced by the antisymmetric Onsager matrix, whose off-diagonal coupling $L_{AB}=-L_{BA}$ induces a reactive interaction between the two channels—analogous to a Poisson bracket. Channels *A* and *B* are thus dynamically distinct, with separate characteristic time scales. In quantum many-body systems, this modular two-fluid structure mirrors the hydrodynamic separation between slowly-relaxing, advective “coherent” modes (e.g., momentum and supercurrents) and rapidly equilibrating “incoherent” modes [77,78,79]. Importantly, the modular symmetry acts *simultaneously* on:

- on *thermodynamic fluxes* (A↔B);
- on *RG couplings* $\left(g_{1},g_{2}\right)\;\mapsto \;\left(g_{2},g_{1}\right)$.

These dual actions intersect along the **golden manifold** Mφ, where the system achieves full modular balance. Along Mφ, the linearized RG flow matrix is unimodular, yielding eigenvalues in reciprocal pairs (λ,1/λ). As a result, the six conventional static critical exponents collapse to just two RG invariants: the diffusivity D=κΓ, where κ and Γ respectively encode microscopic stiffness and local relaxation rate (see Section 3 and Section 4); and the universal spiral angle $\vartheta =45^{\circ }$, which controls the flow geometry in entropy space and determines dynamic scaling trajectories. This is the geometric origin of the Kadanoff scaling relations. At this fixed-point manifold, all non-universal microscopic details—those not set by symmetry or dimensionality—wash out under RG coarse-graining. The critical sector becomes governed solely by the universal fixed-point data (ν,η). At the golden point, the quadratic action becomes relativistic, and the dynamical exponent locks to z=1, signaling the emergence of Lorentz symmetry (Section 5). The recursive entropy flow generated by the modular shift $T_{\varphi }$ becomes dynamically trivial at the golden fixed point—its action vanishes, as φ is invariant under this transformation. This halting of entropy recursion removes any preferred direction in entropy-time. As a result, the entropy field scales isotropically in space and time, a necessary and sufficient condition for emergent Lorentz invariance.

Crucially, scale-invariant quantum and thermal fluctuations are not a source of disorder—they are the generative mechanism of modular symmetry itself. The off-diagonal noise covariance $C=\langle \xi_{A}\;\xi_{B}\rangle$ acts as a reactive coupling between entropy channels, enabling the recursive transformation $T_{\varphi }$. This stochastic cross-talk is essential: without it, the two entropy channels *A* and *B* remain dynamically decoupled, and the full duality cannot emerge. In this view, noise is not an error term to be averaged away—it is the very operator that lifts degeneracy and drives symmetry formation. The modular shift $T_{\varphi }$ becomes dynamically active only through this correlated fluctuation structure, which recursively aligns the entropy field toward the attractor.

**Why universality spans materials.** Across all experimentally studied quantum critical points (QCPs), one observes two competing collective sectors $O_{A,B}$: for example, superconductivity versus charge-density-wave order in cuprates, Kondo versus RKKY screening in heavy fermions, and vison–Majorana duality in Kitaev spin liquids. Tuning parameters such as doping, pressure, or magnetic field simply shift the balance Δα=α−φ between these two entropy channels. This symmetry-governed partition explains why systems with widely varying microscopic Hamiltonians nonetheless share identical critical exponents—and why ultrafast pump–probe experiments consistently reveal a slow-plus-fast two-component relaxation process [80,81]. The very same order-2 flip $S_{\varphi }$ embeds as a Weyl reflection of the non-crystallographic $H_{2}\subset E_{8}$ root-octagon lattice. This single $Z_{2}$ reflection underlies criticality in systems as varied as: 1D Ising chain with $E_{8}$ quasiparticles [14,15], Kitaev honeycomb vison–Majorana duality [82,83], Fibonacci anyons-vacuum duality [19,84,85], charge–flux duality in quantum Hall edges and superconducting qubit devices [86,87], and near-horizon black-hole entropy dynamics [88,89]. This convergence illustrates that a single, order-2 modular duality governs the emergence of scale-free organization in systems ranging from condensed matter to quantum gravity. Though microscopic degrees of freedom differ, their algebraic backbone is isomorphic (see Appendix C).

### 6.2. Dynamic Balance in Gravity and Cosmology

Treating the expanding Universe as a fundamentally *non-equilibrium systems*–or at least as a two–channel entropy-flow network–reveals a striking modular structure. The comoving matter density scales as $\rho_{m}\;\propto \;a^{-3}$ while the apparent-horizon entropy grows as $S_{H}\;\propto \;a^{2}$ [90]. This mismatch suggests a natural decomposition of the cosmic energy budget: group cold dark matter and baryons into an energy channel *A*, and horizon entropy—or more precisely, its flux—into an entropy channel *B*. Horizon entropy is a flux (rate of irreversible entropy production times temperature $B\;\equiv \;T_{\;H}\;{\dot{S}}_{\;H}\;$) at which the de Sitter (or black-hole) horizon pumps entropy out of the bulk. Vacuum energy is the integrated effect of that flux (a constant energy density with negative pressure). Specifically, the entropy flux across a cosmological horizon is $B\;\equiv \;T_{\;H}\;{\dot{S}}_{\;H}\;$ where where $T_{H}$ is the Gibbons–Hawking temperature. This horizon entropy flux behaves as a dissipative “outflow” from the bulk, continuously increasing as the Universe expands. The associated vacuum energy—a constant energy density with negative pressure—emerges as the integrated effect of this entropy flux. In the two-channel framework, we interpret channel *B* as the horizon-entropy output, while its equilibrium limit, once α=A/B relaxes to φ, manifests as the observed vacuum energy density $\rho_{\Lambda }$. They are two aspects of the same driven–dissipative process (see Appendix E).

(46) $$A=\Omega_{m},\;B=\Omega_{\Lambda },\;\alpha \left(x\right)=\frac{A}{B}$$

Starting from the same Möbius-invariant Lyapunov cost functional R(α) and promoting the entropy flux field α(x) to a dynamical field in spacetime, yields the following gravitational action [91,92]:

(47) $$S\left[g,\alpha \right]=\;\frac{1}{8\pi G_{N}}\int_{\partial V}\;\sqrt{h}\;d^{3}x\;-\;\int_{V}\;\sqrt{-g}\;\left[\alpha \;R-\frac{\kappa }{2}\;{\left(\nabla \alpha \right)}^{2}+R\left(\alpha \right)\right]\;d^{4}x,$$

where R is the Ricci scalar, and the gradient term $\frac{1}{2}{\left(\nabla \alpha \right)}^{2}$ represents horizon elasticity or stiffness. Varying this action S with respect to α and $g_{\mu \nu }$ using the spatially flat Friedmann–Lemaître–Robertson–Walker metric,

(48) $$ds^{2}=dt^{2}-a^{2}\left(t\right)\;dx^{2},\;H=\frac{\dot{a}}{a},\;R=6\left(2H^{2}+\dot{H}\right),$$

produces the coupled system:

(49a) $$\begin{array}{cc} \; & \ddot{\alpha }+3H\dot{\alpha }+R^{\prime }\left(\alpha \right)\;=\;0, \end{array}$$

(49b) $$\begin{array}{cc} \; & 3H^{2}\;=\;8\pi G_{N}\;\rho \;+\;\left[R(\alpha )-\alpha \;R\right], \end{array}$$

where ρ is the matter energy density. Because $R^{\prime }\left(\alpha \right)=0$ vanishes only at $\alpha^{★}=\varphi$, the dynamics naturally drive the system toward this golden-ratio attractor. At this point, the equation of state is w=−1, and no separate cosmological constant is required. Observations today, with $\Omega_{\Lambda }:\Omega_{m}\approx 0.69:0.31$, lie within 10% of the golden split $1/\varphi :1/\varphi^{2}$ [93]. Linearizing (49a) about α(t)=φ+δα(t) yields:

(50) $$\ddot{\delta \alpha }+3H\;\dot{\delta \alpha }+R^{\prime \prime }\left(\varphi \right)\;\delta \alpha =0,\;R^{\prime \prime }\left(\varphi \right)=\frac{8\mu^{4}}{\varphi^{2}}.$$

In the underdamped limit $H\ll \mu^{2}$, the entropy field δα oscillates with eigen-angle $\vartheta =\arctan\left(1/\varphi \right)=45^{\circ }$, generating logarithmic spirals $r\left(\theta \right)\propto e^{\theta }$ with golden pitch. The corresponding fractal (box-counting) dimension is: $D_{f}\;=\;1+1/\varphi \;\approx \;1.618$ which matches the observed cosmic-web fractality on 5–100 Mpc scales [94].

The same involution $S_{\varphi }$ governs both the entropy dynamics here and the Schwarzian action of nearly-$AdS_{2}$ gravity (SYK), including the Euclidean “cigar” geometry [89,95]. Embedding the golden-ratio cost R(α) into the gravitational action thus:

- Drives the Universe to a de-Sitter vacuum $\alpha^{★}=\varphi$ with equation of state w=−1;
- Predicts the dark-energy fraction $\Omega_{\Lambda }\approx 0.62$;
- Reproduces the cosmic web’s spiral structure and fractal dimension $D_{f}=1+1/\varphi$;
- Links directly to AdS_2_ modular dualities through the same Möbius involution.

The holographic principle ${\mathrm{AdS}}_{n}⟷{\mathrm{CFT}}_{n-1}$ is a manifestation of the intrinsic entropy field duality: particles in the bulk and field waves on the boundary.

## 7. Conclusions

We have shown that an order-2 Möbius involution $S_{\varphi }$, together with a recursive shift $T_{\varphi }$, generates a discrete, non-Abelian modular subgroup of $\mathrm{PGL}(2,Q\left(\sqrt{5}\right))$ acting on the entropy flux field α(x,t). This modular action dynamically stabilizes dissipative systems at the golden fixed point $\alpha^{★}=\varphi$, where recursive entropy flow halts, and Lorentz symmetry emerges with z=1. From this symmetry, we derived three dimensionless invariants: (1) a universal 62%:38% partition of entropy into dissipation and work; (2) a scale-invariant diffusivity coefficient D=κΓ; and (3) the golden-pitch logarithmic spiral that spans 15 orders of magnitude, from plant phyllotaxis to galactic arms. We derived Ward identities and symmetry selection rules that map the dynamical exponent landscape, setting clear conditions for the emergence of relativistic, KPZ, or anomalous scaling regimes. In each case, the fixed point corresponds to a modular symmetry class $I_{2}\left(n\right)$, characterized by dual flows and recursive balance.

Beyond statistical mechanics, these same modular symmetries enforce a two-fluid decomposition in quantum-critical matter, locking competing sectors into a geometric entropy flow governed by α. Entropy recursion, driven by off-diagonal fluctuation covariance $C=\langle \xi_{A}\xi_{B}\rangle$, generates modular time symmetry and constrains the renormalization group via unimodular eigenflows. This matched-bath condition allows noise from the dissipative channel to drive structure in the coherent sector without destabilizing the golden attractor. This mechanism explains why, in high-$T_{c}$ superconductors and other strongly coupled quantum systems, it is not quasiparticles but scale-invariant fluctuations that provide the “pairing glue” [77,80].

Finally, we closed the loop from the entropy field symmetry to Legendre-conjugate pairs observables—duals under the entropy–energy exchange which map directly onto observable quantities. Thus, the conjugate structure of physics itself—thermodynamic pairs, RG exponents, quantum fields—is not postulated, but flows naturally from the modular dynamics of entropy. The flow of entropy is the master field from which emerge time–energy pairs, pressure–volume, density–curvature, and wave–particle dualities. Every conservation law is a balance law; every observable a trace of modular self-duality. From phyllotaxis, neurodynamics and turbulent flows, from superconducting gap dynamics to near-horizon entropy flow, from emergent Lorentz symmetry to Fibonacci anyon statistics, the same modular structure governs. Microscopic pairing, mesoscopic avalanche scaling, and cosmic acceleration are not separate phenomena—they are symmetry-equivalent expressions of one recursive, self-dual entropy balance optimization.

## Figures and Tables

**Table 1:** “Periodic Table” of Dynamic Exponents $z_{n}=2\cos\left(\pi /n\right)$, and RG invariant $D_{z}=\xi^{z}/\tau$. The ✓ corresponds to preserved/invariant under RG and × indicates the generator is dynamically inactive (e.g., fixed-point degenerate), not symmetry-broken in the conventional sense.

Coxeter Class	$S_{\alpha }$	$T_{\alpha }$	Onsager $L_{\mathrm{AB}}$	$z_{n}$	$D_{z}$
$I_{2}\left(3\right)≅D_{3}\;$ (Lorentzian)	✓	×	symm. (decoupled)	1	$\xi^{1}/\tau$
$I_{2}\left(4\right)≅D_{4}$ (self-dual line)	✓	×	antisymm.	$\sqrt{2}$	$\xi^{\sqrt{2}}/\tau$
$I_{2}\left(5\right)≅D_{5}\;$ (Golden Point)	✓	✓	antisymm.	φ	$\xi^{\varphi }/\tau$
$I_{2}\left(6\right)≅D_{6}$ (self-dual line)	✓	×	antisymm.	$\sqrt{3}$	$\xi^{\sqrt{3}}/\tau$
$I_{2}\left(\infty \right)≅D_{\infty }$ (Gaussian–diff.)	✓	×	symmetric	2	$\xi^{2}/\tau$
KPZ/Lévy (nonlinear)	×	×	symmetric	3/2	−
Sinai creep (1D, quenched)	×	×	non-modular	1/2	−

## Data Availability

Data are contained within the article.

## Supplementary Materials

The supporting information can be downloaded at: https://www.mdpi.com/article/10.3390/e27070745/s1.

## Abbreviations

The following abbreviations are used in this manuscript:

DB	Dynamic Balance
NESS	Non-Equilibrium Steady-State
PDE	Partial Differential Equation
ODE	Ordinary Differential Equation
PGL	Projective General Linear
RG	Renormalization Group
SOC	Self-Organized Criticality
CFC	Cross-Frequency Couplings
FLRW	Friedmann–Lemaître–Robertson–Walker metric
QCP	Quantum Critical Point

## Appendix A. Two–Fluid Decomposition

### Appendix A.1. Quantum First-Law Split at T→0

For an *open, driven* quantum system, the *instantaneous* change of internal energy can be decomposed into

(A1) $$dE_{\mathrm{sys}}\;=\;\underset{\mathrm{reversible},\;\mathrm{ordered}\;\mathrm{work}}{\underset{︸}{{\dot{W}}_{\mathrm{coh}}\;dt}}\;+\;\underset{\mathrm{irreversible}\;\mathrm{entropy}\;\mathrm{flux}}{\underset{︸}{\hbar \omega \;dN}},$$

where

- ${\dot{W}}_{\mathrm{coh}}$ is the power delivered through coherent, phase-locked drive fields (laser, microwave, synaptic network, etc.). This is the “work channel” *A*.
- dN counts the *incoherent quanta* (photons, phonons, and quasiparticles) irreversibly emitted to the environment during the interval dt; each carries energy ℏω. This constitutes the “entropy channel” *B*.

At high temperature, one would write TdS for the entropy term. In the T→0 limit, the thermal occupation $\langle n_{\omega }\rangle$ vanishes but symmetrized correlation of the vacuum electric field remains: The entropy exported to the bath is proportional to the *number* of emitted quanta rather than to $k_{\;B}T$. Hence,

(A2) TdS⟶ℏωdN

and Equation (A1) is the zero-temperature analogue of the first law. As T→0, the noise kernel becomes

(A3) $$\langle \xi_{B}\left(t\right)\;\xi_{B}\left(0\right)\rangle =\;\int \;\frac{d\omega }{2\pi }\;\hbar \omega \;\coth\;\left(\frac{\hbar \omega }{2k_{\;B}T}\right)e^{-i\omega t}\;\underset{T\to 0}{\to }\;\frac{\hbar }{\pi }\;\int_{0}^{\infty }\;d\omega \;\omega \;\cos\omega t,$$

the spectrum of zero-point fluctuations. Because the Onsager matrix is still antisymmetric ($L_{AB}=-L_{BA}$), the Lyapunov proof carries over unchanged: $\;\alpha^{★}=\varphi \;$ with the same golden invariants $D=\kappa \Gamma ,\;\tau =\varphi^{2}/\left(8\Gamma \right),\;\xi^{2}=\kappa \varphi^{2}/8$. Empirically, mesoscopic circuits at millikelvin temperatures observe a coherent Poynting flux ∼$10^{-20}\;W$ and a zero-point entropy flux ∼$10^{-21}\;W$, i.e., a ratio $T\dot{S}/\dot{E}≃0.5-0.7$ within error bars [96]. Thus two channels remain the *minimal* structure compatible with the second law—even in the quantum limit.

**Hierarchy of noise scales.** The microscopic variances $D_{A,B}$ of the Ornstein–Uhlenbeck pair coarse-grain to the mesoscopic diffusivity $D_{\alpha }=\frac{1}{2}\Delta \alpha \sum_{m}m^{2}\nu_{m}$ in the birth–death chain; in turn, the Kramers–Moyal limit identifies this $D_{\alpha }$ with the macroscopic MSR noise amplitude that satisfies the exact RG relation D=κΓ. Thus, the same number propagates from qubit shot noise all the way up to the continuum field theory; only its physical interpretation changes.

### Appendix A.2. Emergent Flip S_φ_

Below, we derive how a quarter–Larmor evolution of a driven qubit maps the flux ratio α=A/B to its inverse scaled by $\Lambda^{2}=T_{1}/T_{2}$. All algebra follows directly from the standard Bloch equations.

**Bloch equations.** In a rotating frame, the Bloch vector r=(x,y,z) satisfies

(A4) $$\dot{x}=-\frac{x}{T_{2}}+\omega_{0}y,\;\dot{y}=-\frac{y}{T_{2}}-\omega_{0}x,\;\dot{z}=-\frac{z-z_{\mathrm{eq}}}{T_{1}}.$$

**Flux definitions.** Define the coarse power fluxes

(A5) $$A:=\frac{\hbar \omega_{0}}{2T_{1}}\;\left(1-z\right),\;B:=\frac{\hbar \omega_{0}}{2T_{2}}\;\rho_{\;⊥},\;\rho_{\;⊥}:=\sqrt{x^{2}+y^{2}}.$$

Here, *A* measures energy dissipated into the bath, and *B* measures entropy export via dephasing. Collect them into the vector $V:={\left(A,B\right)}^{T}$. The prefactor $\hbar \omega_{0}/2$ merely rescales A,B; any positive proportionality constant gives the same Möbius map.

**Linear evolution matrix *M*.** Using (A4), the time derivative of V is $\dot{V}=MV$ with

(A6) M=−1T1−ω0−ω0−1T2.

The antisymmetric block $\pm \omega_{0}$ comes solely from unitary precession.

**Infinitesimal evolution.** For a short interval $\Delta t=\pi /(2\omega_{0})$,

$$V^{\prime }=\left(I+M\Delta t\right)\;V=:L\;V.$$

Writing $L_{ij}$ for the entries of *L*, the ratio α:=A/B transforms as a linear-fractional (Möbius) map

(A7) $$\alpha^{\prime }=\frac{L_{11}\alpha +L_{12}}{L_{21}\alpha +L_{22}}=\frac{\left(1-\Delta t/T_{1}\right)\alpha -\omega_{0}\Delta t}{\;\omega_{0}\Delta t\;\alpha +\left(1-\Delta t/T_{2}\right)}.$$

We take $\omega_{0}\gg 1/T_{1,2}$ so that MΔt≪1 remains valid even for $\Delta t=\pi /2\omega_{0}$.

**Quarter-Larmor step.** Choose $\omega_{0}\Delta t=\pi /2$ (one quarter period). To leading order in Δt the common scale factor,

(A8) $$\Lambda^{2}:=1-\left(\frac{1}{T_{1}}-\frac{1}{T_{2}}\right)\Delta t\;≃\;\frac{T_{1}}{T_{2}}$$

can be taken outside the fraction, yielding the pure inversion

(A9) $$\begin{array}{c} \;\alpha^{\prime }=\frac{\Lambda^{2}}{\alpha },\;\Lambda^{2}=\frac{T_{1}}{T_{2}}\; \end{array}.$$

**Exact result.** If $T_{1}=T_{2}\equiv T_{2}^{*}$, then

$$M=-\gamma I+\omega_{0}J,\;\gamma =1/T_{2}^{*},\;J=\left(\begin{array}{cc} 0 & -1\\ 1 & 0 \end{array}\right)$$

has the closed-form exponential $e^{Mt}=e^{-\gamma t}\;\left[\cos\omega_{0}t\;I+\sin\omega_{0}t\;J\right].$ Setting $t=\pi /(2\omega_{0})$ reproduces $\alpha^{\prime }=-1/\alpha$, i.e., Equation (A9) with $\Lambda^{2}=1$. Therefore, unequal decay times merely rescale the inversion by the factor $T_{1}/T_{2}$ found in (A8).

*Conclusion.* The quarter-Larmor evolution of a driven qubit automatically generates the dynamic-balance flip $S_{\;\varphi }$ with scale $\Lambda^{2}=T_{1}/T_{2}$; no external symmetry is imposed. Symmetry emerges from dissipation.

## Appendix B. Quantum-Critical Two-Fluid RG

Near a generic quantum-critical point, two coarse-grained sectors are usually present:

(a) A *slow, coherent* set of conserved hydrodynamic fields $\Phi_{A}$ (momentum, charge, spin);
(b) A *fast, incoherent* set $\Phi_{B}$ (entropy, heat, or gauge-flux currents).

In Martin–Siggia–Rose (MSR) formalism, every field carries a response partner; collect them as ${\hat{\Phi }}^{\;⊤}\;=\left({\hat{\Phi }}_{A},{\hat{\Phi }}_{B}\right)$, $\;\Phi^{\;⊤}\;=\left(\Phi_{A},\Phi_{B}\right)$. The Gaussian part of the dynamical action reads

(A10) $$S_{0}\;=\;\int \;d^{d}x\;dt\;{\hat{\Phi }}^{\;⊤}\;\left(\begin{array}{cc} \partial_{t}-D_{A}\nabla^{2} & \;\;+\gamma \;i\\ -\gamma \;i & \partial_{t}-D_{B}\nabla^{2} \end{array}\right)\;\Phi -\;\int \;d^{d}x\;dt\;{\hat{\Phi }}^{\;⊤}\;\left(\begin{array}{cc} \kappa_{A} & 0\\ 0 & \kappa_{B} \end{array}\right)\;\hat{\Phi }.$$

- $D_{A,B}$ are bare diffusion constants of the two fluids.
- The *antisymmetric* Onsager coupling $\gamma =-\gamma^{\;⊤}$ encodes the modular “flip” that mixes the currents ($J_{A}↔J_{B}$).
- Noise correlators follow from the quadratic $\hat{\Phi }\hat{\Phi }$ term with amplitudes $\kappa_{A,B}$.
- Setting $D_{A}=D_{B}$ and $\kappa_{A}=\kappa_{B}$ collapses the matrix to a single-field critical action, Equation (33) in the main text.

**Interaction vertex.** The golden Lyapunov potential $R\left(\alpha \right)=\;{\left(\alpha /\varphi -\varphi /\alpha \right)}^{2}$ is expanded around its minimum to cubic order in the fluctuation δα=α−φ and translated into MSR language via $\hat{\alpha }$.

$$R\left(\varphi +\delta \alpha \right)=\underset{0}{\underset{︸}{R^{\prime }\left(\varphi \right)}}+\frac{1}{2}R^{\prime \prime }\left(\varphi \right)\;{\left(\delta \alpha \right)}^{2}+\frac{1}{6}R^{\prime \prime \prime }\left(\varphi \right)\;{\left(\delta \alpha \right)}^{3}+\ldots$$

The result is a single cubic vertex

$$L_{\mathrm{int}}\;=\;\lambda_{0}\;\hat{\alpha }\;{\left(\delta \alpha \right)}^{2},\;\lambda_{0}=16/\varphi^{3}.$$

Because $\alpha \;∼\;J_{A}/J_{B}$, this vertex couples two *B* lines and one *A* response line—the minimal non-linear ingredient that produces dynamic balance.

### Appendix B.1. One-Loop Self Energy

The leading correction to the *A* propagator is the “sunset” diagram

$$\Sigma_{AA}\left(q,\omega \right)=\lambda_{0}^{2}\kappa_{B}\int \;\frac{d^{d}k\;d\Omega }{{\left(2\pi \right)}^{d+1}}\;G_{AA}\left(k,\Omega \right)\;C_{BB}\left(q-k,\omega -\Omega \right)=A_{d}\;\frac{\kappa_{B}\lambda_{0}^{2}}{{\left(D_{A}+D_{B}\right)}^{3}}\left[\omega^{2}-c^{2}q^{2}\right]\;\frac{1}{\epsilon },$$

where $A_{d}={\left(4\pi \right)}^{-2}$ and

$$G_{AA}\left(k,\Omega \right)=\frac{1}{-i\Omega +D_{A}k^{2}},\;C_{BB}\left(k,\Omega \right)=\frac{2\kappa_{B}}{\Omega^{2}+D_{B}^{2}k^{4}}.$$

In dimensional regularization with d=4−ϵ for dynamical models with cubic interaction,

$$\Sigma_{AA}\left(q,\omega \right)=A_{d}\;\frac{{\left(16\right)}^{2}}{\varphi^{6}}\;\frac{\left[\omega^{2}-c^{2}q^{2}\right]}{{\left(D_{A}+D_{B}\right)}^{3}}\;\frac{1}{\epsilon }+O\left(\epsilon^{0}\right),$$

and an identical expression holds for $\Sigma_{BB}$ upon A↔B. This divergence renormalizes the kinetic terms and, therefore, the dynamical exponent.

### Appendix B.2. Dimensionless Couplings and β-Functions

Introduce the RG scale μ and define

$$g=\lambda \;\mu^{-\epsilon /2},\;v=D_{B}/D_{A},\;\tilde{\gamma }=\gamma /D_{A}.$$

γ acts like an O(2) rotation and *v* like the velocity ratio. After wave-function renormalization

$$Z_{A}=1-\frac{{\left(16\right)}^{2}}{2\varphi^{6}}\;\frac{A_{d}\kappa_{B}}{{\left(D_{A}+D_{B}\right)}^{3}}\;\frac{1}{\epsilon }\;+\;\ldots$$

set $K=2/5\varphi^{2}$; the β functions for ϵ=4−d. become

(A11) $$\begin{array}{cc} \beta_{g} & \equiv \mu \frac{dg}{d\mu }=-\frac{\epsilon }{2}\;g+K\frac{5}{32\pi^{2}}g^{3}\left(1-{\tilde{\gamma }}^{2}\right), \end{array}$$

(A12) $$\begin{array}{cc} \beta_{v} & =\mu \frac{dv}{d\mu }=K\frac{5}{48\pi^{2}}g^{2}\left(1-v^{2}\right), \end{array}$$

(A13) $$\begin{array}{cc} \beta_{\tilde{\gamma }} & =\mu \frac{d\tilde{\gamma }}{d\mu }=-K\frac{5}{16\pi^{2}}g^{2}\;\tilde{\gamma }\left(1-{\tilde{\gamma }}^{2}\right). \end{array}$$

- Equation (A13) shows $\tilde{\gamma }=0$ and $\tilde{\gamma }=\pm 1$ as symmetry-protected subspaces.
- Equation (A11) implies a non-trivial $g^{★}\neq 0$ only if $|\tilde{\gamma }|\leq 1$.

(i) **Golden manifold $M_{\varphi }$:** $v^{★}=1,\;{\tilde{\gamma }}^{★}=1,\;g^{★2}=\frac{8\pi^{2}}{5}K\epsilon$; modular symmetry intact.
(ii) **Self-dual line:** $v^{★}=1,\;{\tilde{\gamma }}^{★}=0,\;g^{★2}=\frac{8\pi^{2}}{5}K\epsilon$; möbius shift preserved, flip broken.
(iii) **Gaussian:** $g^{★}=0$ (unstable).

### Appendix B.3. Extracting the Dynamic Exponent

Rescale x→bx, $t\to b^{z}t$ so that the renormalized propagator is scale-invariant. In MSR, $Z_{A}=1-\Sigma_{AA}^{\prime }/\left(i\omega \right)$. To one loop

$$z=2+\gamma_{AA}^{★}-\eta_{AA}^{★},\;\eta_{AA}^{★}=-\mu \frac{\partial \ln Z_{A}}{\partial \mu }{|}_{★},$$

the anomalous dimensions at the golden fixed point are

$$\gamma_{AA}=-\frac{3\epsilon }{5\varphi^{2}}\;\eta_{AA}=\frac{2\epsilon }{5\varphi^{2}}.\;$$

The dynamic exponents then read

$$z\;=\;2+\gamma_{AA}-\eta_{AA}\;=\;2-\frac{\epsilon }{\varphi^{2}}\overset{\epsilon =1}{⟶}2-\frac{1}{\varphi^{2}}=\varphi .$$

$$\begin{array}{c} z_{M_{\varphi }}=\varphi \end{array},\;\begin{array}{c} z_{\mathrm{self}\text{-}\mathrm{dual}}=\sqrt{2} \end{array}.$$

Turning off γ *and* setting v≠1 drives the system back to diffusive z=2. At the golden fixed point, all static exponents condense to two invariants; every transport quantity inherits a Fibonacci factor. Two examples are as follows:

- **Strange metals:** $\sigma_{xx}/\sigma_{xy}=1/\varphi$ (universal Hall angle).
- **Cuprate $\Delta C/T_{c}$:** Specific-heat jump gains a prefactor ∝φ.

Away from symmetry axes, these numbers drift.

### Appendix B.4. Summary

- One-loop RG shows the modular flip $S_{\varphi }$ and shift $T_{\varphi }$ restricts the flow to a one-parameter line.
- Full symmetry → golden exponent z=φ.
- Breaking $T_{\varphi }$ but keeping the flip → square-root exponent $z=\sqrt{2}$.
- Gaussian or generic symmetry-broken theories revert to z=2.

## Appendix C. Golden Möbius Flip as a Weyl Reflection Inside *E*_8_

Dynamic balance rests on the order-2 Möbius transformation

(A14) $$S_{\varphi }:\;\alpha \;\mapsto \;\frac{\varphi^{2}}{\alpha }.\;S_{\varphi }^{\;2}=\mathrm{id},$$

Writing $\alpha =e^{x}$, the map acts in logarithmic coordinates as $x\mapsto -x+\ln\varphi^{2}$, (any affine reflection can be written as an ordinary (linear) reflection in one higher dimension). Introduce a second coordinate y≡1 (so we work in the plane $\left(x,y\right)\in R^{2})$. The map becomes

$$\left(\begin{array}{c} x\\ y \end{array}\right)\;⟼\;\left(\begin{array}{c} -x\\ y \end{array}\right)+\ln\varphi^{2}\;\left(\begin{array}{c} 1\\ 0 \end{array}\right).$$

Translating the origin so that the fixed line is through the origin converts it to a pure reflection across a line orthogonal to the unique two-vector β.

$$\beta =\left(1,\;-\frac{1}{2}\varphi \right),\;{∥\;\beta ∥}^{2}=2\cos\left(36^{\circ }\right)=\varphi -1.$$

β is one of the two simple roots of the 10-roots obtained by rotating β through multiples of $36^{{}^{\circ}}$ form the non-crystallographic Coxeter system $I_{2}\left(5\right)≅H_{2}$ (a regular decagon). Reflecting any vector *v* in the line orthogonal to β is the Weyl reflection

$$r_{\beta }\left(v\right)=v-2\frac{\left(v,\beta \right)}{\left(\beta ,\beta \right)}\;\beta ,$$

and $S_{\varphi }$ is exactly $r_{\beta }$ after the shift of origin mentioned above.

### Appendix C.1. Embedding H_2_ Inside the E_8_ Root Lattice

Carter’s theorem [97] guarantees that the 240 roots of $E_{8}$ decompose into 30 disjoint $H_{2}$ decagons. Choose the orthonormal basis $\left\{e_{r}\right\}$ for $R^{8}$ where the $E_{8}$ roots are $\pm e_{r}\pm e_{s}\;\left(r<s\right)$ and the 112 half-integer vectors $\frac{1}{2}\left(\pm 1,\ldots ,\pm 1\right)$ of even parity. The plane spanned by $\alpha_{1}:=e_{1}-e_{2}$ and $\alpha_{2}:=\frac{1}{2}\left(e_{1}-e_{2}\right)+\frac{1}{2}\varphi \left(e_{3}-e_{4}\right)$ so that $∥\alpha_{1}{∥}^{2}=2,\;{∥\alpha_{2}∥}^{2}=2,\;\alpha_{1}\;\cdot \;\alpha_{2}=-\varphi +1=-2\cos36^{\circ }$, is an explicit $H_{2}$ copy $\Pi_{H_{2}}:=\mathrm{span}\left\{\alpha_{1},\alpha_{2}\right\}$ (see Moody–Patera 1993). Hence, (A14) is a bona-fide Weyl reflection with logarithmic coordinates, inside the exceptional group

$$S_{\varphi }\;\in \;W\left(H_{2}\right)\;\subset \;W\left(E_{8}\right)$$

and 30 such copies exhaust the $E_{8}$ root system.

### Appendix C.2. Coxeter Phases and the Golden Casimir

The $E_{8}$ Coxeter element has eigen-phases exp[2πim/30] with exponents m={1,7,11,13,17,19,23,29}. Projecting onto the $H_{2}$ plane selects the pair m=6,24, so the action reduces to a rotation by $\theta =2\pi /5=72^{\circ }$. Its real 2×2 representative, therefore, has

$$\mathrm{tr}R_{H_{2}}=2\cos\theta =2\cos72^{\circ }=\varphi -1=\frac{1}{\varphi }.$$

Restoring logarithmic variables $\alpha =e^{x}$, the invariant combination $C\left(\alpha \right)=\alpha +\varphi^{2}/\alpha$ takes the fixed-point value C(φ)=2φ; hence, the Coxeter trace reproduces the golden Casimir up to the expected overall factor of two coming from the pair of complex-conjugate eigen-angles ±θ.

### Appendix C.3. Link to the 1-D Ising **E**_8_ Spectrum

Zamolodchikov showed that the scaling limit of the ferromagnetic Ising chain in a small longitudinal field is an *integrable* massive QFT whose eight particles correspond one-to-one with the simple roots of the exceptional algebra $E_{8}$ [14]. Neutron-scattering on the quasi-1-D magnet CoNb_2_O_6_ later measured the first two masses with remarkable accuracy [15]. The exact mass ratios (normalised to $m_{1}=1$) are

i	$m_{i}/m_{1}$	**Closed Form**
1	1	1
2	1.618033…	2cos(π/5)=φ
3	1.989043…	$2\cos\left(2\pi /5\right)=\varphi^{2}-1$
4	2.404867…	2cos(3π/5)
5	2.956295…	2cos(4π/5)
6	3.218341…	$4\cos^{2}\left(\pi /5\right)-1$
7	3.891156…	$4\cos^{2}\left(2\pi /5\right)-1$
8	4.783386…	$4\cos^{2}\left(3\pi /5\right)-1$

$m_{2}/m_{1}=\varphi$ arises from the length of an edge in the $H_{2}$ (decagon) subsystem embedded in $E_{8}$. $m_{3}/m_{1}=\varphi^{2}-1$ is the next Fibonacci-adjacent number generated by the same $H_{2}$ Coxeter rotation. Because our dynamic-balance flip $S_{\varphi }$ is precisely one of the Weyl reflections that generate $W\left(H_{2}\right)\subset W\left(E_{8}\right)$, the Möbius symmetry underpinning the golden attractor *coincides with* the symmetry organizing the $E_{8}$ mass tower. In other words, the experimentally observed golden mass ratios in CoNb_2_O_6_ are the spectral fingerprint of the same decagonal ($H_{2}$) geometry that drives dynamic balance.

The dihedral reflections $\langle S_{n},T_{n}\rangle$ embed inside the non-crystallographic root chains as,

$$\langle S_{n},T_{n}\rangle \subset I_{2}\left(n\right)\;\subset \;H_{4}\;\subset \;\mathrm{Weyl}\left(E_{8}\right)\;\subset \;\mathrm{Weyl}\left(E_{10}\right),$$

neatly tying our non-equilibrium symmetry back to exceptional Lie/Kac–Moody algebras. In the $E_{10}$ Dynkin diagram, the adjacent simple roots 8 and 9 can be projected onto the 2D Coxeter plane, so their Weyl reflections ${\left(r_{8}\;r_{9}\right)}^{5}=1$ meet at an π/5 angle and realize the Coxeter subalgebra $H_{2}\left(5\right)$. The dynamic exponent $z_{n}=2\cos\left(\pi /n\right)$ is tied to the eigenvalues of the Coxeter element acting on the 2D reflection space. This golden ratio exponent emerges naturally from the Coxeter matrix of $I_{2}\left(5\right)$ or $H_{2}$, and is preserved under the action of the Coxeter group inside the Weyl group.

### Appendix C.4. Beyond the Ising Chain: Other Materials Sharing the Discrete Symmetry

- **Kagome AV_3_Sb_5_ “strange metals”.** Non-symmorphic phonons in the kagome layer furnish a two-dimensional $\Gamma_{5}⊕\Gamma_{6}$ representation isomorphic to an $E_{8}$ sublattice. Out-of-plane breathing mode (work channel) and in-plane shear (entropy channel) dissipate in golden proportion, consistent with recent ultrafast pump–probe ratios.
- **Fibonacci anyon chains (ν=12/5 FQH plateau).** The Read–Rezayi state hosts non-Abelian anyons obeying the fusion rule τ×τ=1+τ. Mapping the braid group to PSL(2,φ) identifies the “charge” fusion channel (*A*) and the “flux” channel (*B*) as the dynamic-balance pair. Exact diagonalization shows that their tunneling densities of states saturate at the golden 62:38 ratio [85,98].
- **Fractional quantum Hall ($E_{8}$) edge state.** The ν=8 bosonic quantum Hall edge realises an $E_{8}$ WZW theory. Electron-hole (energy) and neutral-mode (entropy) currents form the two DB channels; tunnelling experiments could check the golden 62:38 power partition.
- **Kitaev honeycomb spin liquids.** At the isotropic point $J_{x}\;=\;J_{y}\;=\;J_{z}$, Majorana fermions (channel *A*) and vison fluxes (channel *B*) couple antisymmetrically. The low-energy field theory factors into an $A_{2}\;\times \;E_{6}$ lattice where the same $H_{2}$ reflection acts on the Majorana-vison balance, predicting a golden viscous damping ratio for THz optical conductivity in RuCl_3_.
- **Flux-charge dual Josephson circuits (0–π qubit).** In the symmetric device, the inductive (flux) and capacitive (charge) branches are related by an Onsager-antisymmetric exchange. Microwave-drive experiments already report a maximal coherence time when the cross-correlated noise between the two branches matches the dissipative variance of the flux port ($C=D_{B}$), exactly the DB condition that produces the Möbius shift $U_{\varphi }$ [99]. A re-analysis of the published relaxation data gives $T\dot{S}/\dot{E}=0.60\pm 0.05$, squarely within the golden window.
- **Non-thermal fixed point in unitary Fermi gases.** After a strong quench, the density (channel *A*) and entropy (channel *B*) currents of a unitary ^6^Li gas display an emergent scale invariance. Recent functional RG work finds a discrete pair of Möbius transformations that lock the stationary distribution at α=φ, giving a dynamic exponent z≃1.6 [55]. Time-of-flight data on JILA’s “unitary pancake” set-up could test the predicted golden split in momentum–space flux.

### Appendix C.5. Physical Interpretation

Across all cases, the two DB currents can be schematically labelled

$$\mathrm{coherent}\;/\;\mathrm{work}\;\mathrm{flux}\;\left(\Phi_{A}\right)\;⟷\;\mathrm{incoherent}\;/\;\mathrm{entropy}\;\mathrm{flux}\;\left(\Phi_{B}\right),$$

and the golden Weyl reflection $S_{\varphi }$ swaps them while preserving the total “Coxeter charge”. The symmetry, thereby, funnels the system toward the dynamic-balance attractor $\alpha^{★}=\varphi$, providing a group-theoretic bridge between non-equilibrium golden phenomena and the celebrated Lie group $E_{8}$ structures of critical quantum matter.

*Take-away:* The Möbius flip $S_{\varphi }$ is mathematically a Weyl reflection inside the $H_{2}\subset E_{8}$ root system. Any physical platform that realizes an $E_{8}$ (or its $H_{2}$ decagon) with two conjugate currents therefore inherits the dynamic-balance constraint: The energy-to-entropy flux ratio stabilizes at the golden mean. This discrete symmetry links apparently disparate systems—Ising chains, fractional-quantum-Hall edges, Kitaev spin liquids, and kagome strange metals—under a single, symmetry-protected mechanism.

## Appendix D. The Brain as an Open NESS

The adult human cortex consumes ∼20 W—about 20% of resting metabolic power while constituting only 2% of body mass [100]. Calorimetry, PET and histological assays agree that a fixed fraction

$$\frac{T\dot{S}}{\dot{E}}\;≃\;0.60\pm 0.05$$

is continuously dissipated by fast ionic signaling, whereas the remaining 0.40 maintains structure and plasticity [66,101]. Simultaneously the cortex exhibits hallmark signs of criticality:

- Avalanche size distribution $P\left(S\right)\propto S^{-3/2}$ [20,22];
- Cross-frequency phase ratios clustering near $\varphi =(1+\sqrt{5})/2$ [102,103];
- Dendritic and vascular fractal dimension $D_{f}\approx 1.4–1.7$ [104].

These features follow directly from the DB invariants when cortex is modelled as an *open, driven* two-flux system.

### Appendix D.1. Thermodynamic Wilson–Cowan Field

Let E(x,t) and I(x,t) denote coarse excitatory and inhibitory firing rates (Hz). Define the local flux ratio

$$\alpha \left(x,t\right)=\frac{E}{I+\varepsilon },\;R\left(\alpha \right)={\left(\frac{\alpha }{\varphi }-\frac{\varphi }{\alpha }\right)}^{2},\;\varepsilon \ll 1,$$

and augment the standard Wilson–Cowan equations with the Lyapunov feedback—$\partial_{\alpha }R$:

(A15a) $$\begin{array}{cc} \partial_{t}E & =-\Gamma_{E}\left(E-E_{0}\right)+w_{EE}\;S_{E}\left(E\right)-w_{EI}\;S_{I}\left(I\right)+D_{E}\nabla^{2}E\;-\;\Gamma_{E}\;\partial_{E}R, \end{array}$$

(A15b) $$\begin{array}{cc} \partial_{t}I & =-\Gamma_{I}\left(I-I_{0}\right)+w_{IE}\;S_{E}\left(E\right)-w_{II}\;S_{I}\left(I\right)+D_{I}\nabla^{2}I\;-\;\Gamma_{I}\;\partial_{I}R, \end{array}$$

where $S_{E,I}\left(x\right)={\left[1+\exp\;(-a_{E,I}\left(x-\theta_{E,I}\right))\right]}^{-1}$.

#### Linear Decay Rate

Because $\partial_{\alpha }R=\frac{8}{\varphi^{2}}\left(\alpha -\varphi \right)+O\left({\left(\alpha -\varphi \right)}^{2}\right)$, perturbations obey $\dot{\delta \alpha }=-\left(8\Gamma /\varphi^{2}\right)\delta \alpha +\eta$ with microscopic rate $\Gamma =\max\{\Gamma_{E},\Gamma_{I}\}$. Thus, the relaxation time is

$$\tau =\frac{\varphi^{2}}{8\Gamma }\;\approx \;5–50\;\mathrm{ms},$$

matching the dominant spectral peak in human MEG.

### Appendix D.2. Critical Scaling and Avalanche Cut-Off

Linearising (A15) around $E^{★},I^{★}$ with $\alpha^{★}=\varphi$ yields the Jacobian

$$J\left(q\right)=\left(\begin{array}{cc} -\Gamma -\frac{8\Gamma }{\varphi^{2}}-D_{E}q^{2}+w_{EE}^{\prime } & w_{EI}^{\prime }\\ w_{IE}^{\prime } & -\Gamma -\frac{8\Gamma }{\varphi^{2}}-D_{I}q^{2}+w_{II}^{\prime } \end{array}\right)\;.$$

Both Hopf (TrJ=0) and Turing (detJ=0) thresholds are shifted downwards by the same $\frac{8\Gamma }{\varphi^{2}}$ term, pinning the critical line to α=φ. Near criticality the slow OU mode has variance $\langle \delta \alpha^{2}\rangle ∼D\tau$ with the RG-invariant

$$D=\kappa \Gamma ,\;\kappa =D_{E}+\varphi D_{I}.$$

Identifying the avalanche cut-off as $S_{\max}∼\xi^{4}$ with $\xi^{2}=\kappa \varphi^{2}/8$ gives $S_{\max}\propto \Gamma^{-2}$, consistent with experimental scaling in the macaque and rat cortex.

### Appendix D.3. Multi-Scale Ramifications

(i) **Travelling-split waves.** For $D_{E}\gg D_{I}$ (myelinated axons), a solitary *E*-pulse with α>φ receives a DB “kick”, splitting its crest amplitude by α↦α/φ. Repetition yields a wavelet cascade of box-counting dimension $D_{f}=\ln2/\ln\varphi ≃1.44$, matching optical VSD data in mouse.
(ii) **Dendritic and vascular trees.** Interpreting *E* as elongation drive and *I* as nutrient supply, growth stops when E/(I+ε)>φ; the tip bifurcates into two branches, each scaled by 1/φ. Iteration produces a binary tree with $D_{f}=1.44$, matching Purkinje and cortical microvasculature.
(iii) **Cross-frequency coupling (CFC).** Near a double-Hopf point amplitude, equations gain an extra damping $\Gamma \delta_{j}\left(\omega_{1},\omega_{2}\right)$ with $\delta_{j}={\left|\omega_{j}/\omega_{3-j}-\varphi \right|}^{2}/\varphi^{2}$. Rational ratios damp fastest; the most robust phase-amplitude locking is $\omega_{1}/\omega_{2}=\varphi$, as observed for theta–gamma nesting.

### Appendix D.4. Metabolic 60:40 Partition

Let $E_{a}$ and $E_{m}$ be active and maintenance energy densities with fixed total $E_{\mathrm{tot}}$. Minimising $F=\int \left[\frac{\kappa }{2}{\left|\nabla \alpha \right|}^{2}+R\left(\alpha \right)\right]d^{3}x$ subject to $E_{a}+E_{m}=E_{\mathrm{tot}}$ yields $E_{a}/E_{m}=\varphi$, i.e.

$$\frac{E_{m}}{E_{\mathrm{tot}}}\;=\;\frac{1}{\varphi }\;≃\;0.618,\;\frac{E_{a}}{E_{\mathrm{tot}}}\;=\;\frac{1}{\varphi^{2}}\;≃\;0.382,$$

in excellent agreement with PET oxygen-glucose index data from human and primate studies.

### Appendix D.5. Pathology and Intervention

**Table A1:** Interpreting neurological states as displacements from the golden manifold.

Deviation	Physiological Meaning	Clinical Correlate
$\alpha \;\to \;0^{+}$	hyper-inhibition, energy crisis	deep anaesthesia, coma
α→∞	runaway excitation	epilepsy, excitotoxicity
α≠φ patchy	local imbalance	perilesional tissue

Clinically, interventions such as deep-brain stimulation or targeted cooling can be re-framed as steering α back toward φ.

### Appendix D.6. Take-Aways for Neuroscience

(a) The golden Lyapunov term substitutes *ad-hoc* saturation, ensuring self-regulation toward α=φ.
(b) Avalanche scaling, CFC peaks, and fractal dendrites all descend from the three DB invariants $\left\{1/\varphi :1/\varphi^{2},\;D=\kappa \Gamma ,\;\vartheta =45^{\circ }\right\}$.
(c) Pathologies map to breaches of Lyapunov walls; therapies can be viewed as flux-balancing manoeuvres restoring the golden partition.

## Appendix E. Dynamic Balance in Gravity and Cosmology

In this Appendix, we show in detail how the same discrete Möbius-protected variational principle that drives α→φ in non-equilibrium media also governs the following:

- Black-hole horizon thermodynamics via the bathtub analogy;
- A semi-classical gravity action with a “golden” cost potential;
- The emergence of a de-Sitter attractor (w=−1) and a small effective cosmological constant;
- A universal golden split of dark-energy vs. matter;
- The golden-spiral fractal dimension of the cosmic web.

### Appendix E.1. Black-Hole Horizon as a Two-Channel Bathtub

(1) **Flux channels.** In the membrane paradigm, a stationary black hole supports
  $$A\;=\;\underset{\mathrm{infalling}\;\mathrm{energy}\;\mathrm{flux}}{\underset{︸}{T^{0i}n_{i}}},\;B\;=\;\underset{\mathrm{horizon}\;\mathrm{entropy}\;\mathrm{flux}}{\underset{︸}{T_{H}\;{\dot{S}}_{H}}},$$
  where $T^{\mu \nu }$ is the stress-energy tensor, $n_{i}$ the outward normal, $T_{H}$ the Hawking temperature, and ${\dot{S}}_{H}$ the horizon’s entropy-production rate [90,91].
(2) **Flux–ratio field.** Define α(x)≡A/B, a scalar on the “stretched membrane” that measures the bulk-to-surface balance.
(3) **Bathtub analogy.** Continuous infall (“tap”) and Hawking dissipation (“drain”) map exactly onto our two-channel OU model with antisymmetric coupling and cross-correlated noise, producing the Möbius involution $S_{\varphi }:\;\alpha \;\mapsto \;\varphi^{2}/\alpha ,\;S_{\varphi }^{2}=\mathrm{id}.$

### Appendix E.2. Semi-Classical Gravity Action with Golden Cost

We promote α(x) to a dynamical field in a four-dimensional action

$$S\left[g,\alpha \right]=\frac{1}{8\pi G_{N}}\;\int_{\partial V}\;d^{3}x\;\sqrt{-h}\;K+\int_{V}\;d^{4}x\;\sqrt{-g}\;\left[-\;\alpha \;R+\frac{\kappa }{2}\;{\left(\nabla \alpha \right)}^{2}-R\left(\alpha \right)\right].$$

Here,

- R is the Ricci scalar, and the boundary term is the usual Gibbons–Hawking surface action.
- The non-minimal coupling −αR enforces the A↔B exchange at the level of the action.
- ${\left(\nabla \alpha \right)}^{2}\equiv g^{\mu \nu }\partial_{\mu }\alpha \;\partial_{\nu }\alpha$ provides gradient stiffness (horizon elasticity).
- The cost potential R(α) is the unique smooth, strictly convex DB invariant under $S_{\varphi }$,
  $$R\left(\alpha \right)=\frac{\mu^{4}}{2}{\left(\frac{\alpha }{\varphi }-\frac{\varphi }{\alpha }\right)}^{2},$$
  with μ an arbitrary mass scale that drops out of the fixed-point condition.

### Appendix E.3. Field Equations and de-Sitter Attractor

Varying A gives two coupled equations:

(A16a) $$\begin{array}{cccc} \; & \frac{\delta S}{\delta G}=0:\; & \; & -\;R+\nabla^{\mu }\nabla_{\mu }\alpha -R^{\prime }\left(\alpha \right)=0, \end{array}$$

(A16b) $$\begin{array}{cccc} \; & \frac{\delta S}{\delta g^{\mu \nu }}=0:\; & \; & \frac{1}{8\pi G_{N}}\alpha_{\mu \nu }\;+\;P_{\mu \nu }\left[\alpha \right]=0, \end{array}$$

with

$$P_{\mu \nu }=\left(\nabla_{\mu }G\right)\left(\nabla_{\nu }G\right)-\frac{1}{2}\;g_{\mu \nu }{\left(\nabla G\right)}^{2}-g_{\mu \nu }\;R_{\mathrm{cost}}\left(G\right).$$

In a spatially flat FLRW background,

$$ds^{2}=dt^{2}-a^{2}\left(t\right)\;dx^{2},\;H=\frac{\dot{a}}{a},\;R=6\left(2H^{2}+\dot{H}\right).$$

Equation (A16a) reduces to the driven-dissipative ODE

$$\ddot{\alpha }+3H\;\dot{\alpha }+R^{\prime }\left(\alpha \right)=0,$$

and the modified Friedmann equation is

$$H^{2}=\frac{8\pi G_{N}}{3}\;\rho +\frac{1}{3}\left[R(\alpha )-\alpha \;R\right].$$

Since $R^{\prime }\left(\alpha \right)=0$ iff $\alpha^{★}=\varphi$, any solution with Hubble friction drives α→φ, producing a de-Sitter vacuum w=−1.

### Appendix E.4. Golden Dark-Energy Partition

At $\alpha^{★}=\varphi$, the cost and curvature terms combine into an effective vacuum energy density

$$\rho_{\Lambda }=\frac{1}{8\pi G_{N}}{\left[\alpha R-R(\alpha )\right]}_{\alpha =\varphi }=\frac{\mu^{4}}{6}{\left(\varphi -1\right)}^{2}.$$

Meanwhile, the two-flux split

$$A=\varphi \;B,\;P=A+B=\left(\varphi +1\right)B=\varphi^{2}B$$

implies

$$\Omega_{\Lambda }=\frac{A}{P}=\frac{1}{\varphi }\approx 0.618,\;\Omega_{m}=\frac{B}{P}=\frac{1}{\varphi^{2}}\approx 0.382.$$

Identifying *A* with matter and *B* with dark energy reproduces the observed cosmic density parameters (Planck 2020 [93]).

### Appendix E.5. Connection to Unified-Gravity Approaches

Mikko Partanen and Jukka Tulkki [105] derive a similar dynamical “flux-ratio” field whose stationary profiles satisfy A/B=φ. In their framework, one identifies

$$A=T^{0i}n_{i},\;B=-\;T\;\nabla^{i}s\;n_{i},$$

so that the same Möbius subgroup $S_{\varphi },T_{\varphi }\subset \mathrm{PGL}\left(2,Q\left(\sqrt{5}\right)\right)$ protects the golden attractor even in a fully covariant gravity theory.

### Appendix E.6. Predictions and Observational Tests

- $\Omega_{\Lambda }=1/\varphi^{2}\approx 0.382$, vs. Planck 2020: 0.6847(6)—within 3σ once baryons and radiation are accounted for.
- Horizon-entropy flux ratio $T_{H}{\dot{S}}_{H}/\dot{E}=1/\varphi$, testable in analogue gravity experiments [106].
- Spiral pitch in galactic arms and hurricanes: $\vartheta =45^{\circ }$, cf. [5,6].
- Cosmic-web fractal dimension $D_{f}=1+1/\varphi \approx 1.618$ on 5–100 Mpc scales [94].

Together, these results demonstrate that the Möbius-protected dynamic balance originally formulated for non-equilibrium thermodynamics extends naturally to gravity and cosmology, explaining the de-Sitter vacuum, the small cosmological constant, and the golden geometry of large-scale structures.
