# Supplementary material for: Dynamic Balance: A Thermodynamic Principle for the Emergence of the Golden Ratio in Open Non-Equilibrium Steady States
Source: Entropy (Basel). 2025 Jul 11;27(7):745. doi: 10.3390/e27070745 (PMC12294351; doi:10.3390/e27070745)
Supplement: Supplementary file 1 [file entropy-27-00745-s001.zip › entropy-3567655-supplementary.pdf]

A Thermodynamic Foundations of the Flux-Ratio Framework

A.1 Local balance laws

Let $E(\mathbf{x}, t)$ and $S(\mathbf{x}, t)$ denote the coarse-grained energy and entropy densities of an open system occupying a spatial region $\Omega \subset \mathbb{R}^d$. In the absence of sources or sinks in the bulk, these fields obey local continuity equations

$$\partial_t E + \nabla\cdot \mathbf{J}_E = q_E\,, \tag{S1a}$$

$$\partial_t S + \nabla\cdot \mathbf{J}_s = \sigma \geq 0\,. \tag{S1b}$$

Here $\mathbf{J}_E$ is the energy flux, $\mathbf{J}_s$ the entropy flux, q_E any external power input, and σ the local entropy-production rate. The inequality $\sigma \geq 0$ is the local form of the Second Law.

A.2 Definition of fluxes and the ratio α

Integrating (1) over Ω and assuming no flux across its boundary yields

$$\dot{E}(t) \;\equiv\; \int_\Omega q_E \, d^d x, \qquad T(t)\,\dot{S}(t) \;\equiv\; \int_\Omega T(\mathbf{x}, t)\,\sigma(\mathbf{x}, t)\, d^d x,$$

where $T(\mathbf{x}, t)$ is an effective local temperature (from a fluctuation-dissipation estimate or local probe). In a non-equilibrium steady state (NESS) both $\dot{E}$ and $\dot{S}$ approach finite, time-independent values. If $q_E, \sigma \in C^1([0, \infty))$ and the effective temperature $T(\mathbf{x}, t)$ is bounded away from 0, we define

$$A \equiv \dot{E} > 0, \qquad B \equiv T\,\dot{S} > 0, \qquad \alpha \equiv \frac{A}{B} \in (0, \infty). \tag{S2}$$

The strict positivity of A, B follows because sustained driving ($q_E \neq 0$) and irreversible dissipation ($\sigma > 0$) are both required to maintain a NESS.

A.3 Special limits

Perfect isolation If $\dot{E} = \dot{S} = 0$ the system is at equilibrium and α is undefined. Dynamic Balance applies only to driven–dissipative states with both channels finite.

Zero–temperature bath If $T \to 0^+$ but $\dot{E}, \dot{S} > 0$, then $\alpha \to \infty$. This corresponds to the forbidden "rigid" boundary.

Heat death If $\dot{E} \to 0^+$ while $T\dot{S} > 0$, then $\alpha \to 0^+$—the opposite forbidden corner, representing total disorder with no usable energy flux.

A.4 Entropy Production and the Second Law in Open Systems

Consider an open system exchanging energy and matter with one or more reservoirs. Denote by $E(t)$ its internal energy and by $S(t)$ its Gibbs (or von Neumann) entropy. In the presence of external heat (or particle) currents, the total rate of change of entropy splits as

$$\frac{dS}{dt} = \dot{S}_{\text{prod}} + \dot{S}_{\text{exch}},$$

where $\dot{S}_{\text{prod}} \geq 0$ is the irreversible entropy production (internal dissipation), $\dot{S}_{\text{exch}}$ is the net entropy exchanged with the environment.

The Second Law requires $dS/dt \geq 0$ on average, i.e. $\dot{S}_{\text{prod}} \geq -\dot{S}_{\text{exch}}$. For a non-equilibrium steady state (NESS), internal observables have ceased to evolve on average, so $dS_{\text{sys}}/dt = 0$ and $\dot{S}_{\text{prod}} + \dot{S}_{\text{exch}} = 0$, meaning that all entropy produced by irreversible processes is carried away by the environment. Equivalently, $\dot{S}_{\text{prod}} = -\dot{S}_{\text{exch}}$. In many experimental contexts the exchanged entropy flux can be written as $T\,\dot{S}_{\text{exch}} \equiv q_{\text{out}}$, the irreversible heat power to a reservoir at (effective) temperature T. This motivates our identification

$$B \;\equiv\; T\,\dot{S}_{\text{exch}} \;=\; \underbrace{T\,\dot{S}_{\text{prod}}}_{q_{\text{out}}} \;>\; 0,$$

which we call the entropy flux or dissipative channel. The Second Law for the total (system + environment) entropy requires $\dot{S}_{\text{total}} = \dot{S}_{\text{sys}} + \dot{S}_{\text{env}} \geq 0$. In a NESS the system entropy can even decrease ($\dot{S}_{\text{sys}} < 0$) provided the environment's entropy increase exceeds it. However, $T\,\dot{S}$ in (2) always represents *irreversible* dissipation, so $B > 0$ strictly. Likewise, driving requires $\dot{E} > 0$. Therefore, $0 < \alpha < \infty$ and the two limits

$$\alpha \to 0^+ \quad \text{(pure dissipation, no useful power)}, \qquad \alpha \to \infty \quad \text{(undissipated power, no entropy outlet)}$$

are both forbidden in a sustained NESS. The golden-ratio attractor $\alpha^* = \varphi$ therefore lies strictly within the physically accessible domain.

Fluctuation Theorems and Stochastic Thermodynamics. Realistic systems, especially at small scales or short times, exhibit thermal/quantum fluctuations that can transiently defy typical macroscopic expectations. However, on average, the net entropy production remains nonnegative (Jarzynski's equality, Crooks' fluctuation theorem, etc.). In stochastic thermodynamics, each micro-trajectory has an associated entropy production, but only the mean satisfies $\langle \dot{S}_{\text{production}} \rangle \geq 0$.

A.5 Equilibrium vs. non-equilibrium steady states

Equilibrium is characterized by a *static* Boltzmann–Gibbs state $\rho_e \propto e^{-\beta H}$. No net flow of energy/matter occurs, so observables remain time-independent. **Non-equilibrium**, conversely, arises when the system is driven by external forces (e.g., continuous energy input), **dissipates** heat to a reservoir, or time-dependent drives, quenches, or open boundary conditions mismatch the typical equilibrium thermal distribution ($\rho \not\propto e^{-\beta H}$),

In global equilibrium one has $q_E = 0$ and $\sigma = 0$, so both $\dot{E} = \dot{S} = 0$. The ratio α is ill-defined, reflecting the absence of any net flux. By contrast, an open, driven system reaches a NESS only if energy is continuously supplied and irreversibly dissipated. It is precisely in this regime—far from equilibrium but time-stationary—that the dimensionless ratio (2) provides a meaningful measure of how the system partitions incoming power between *useful* work (or coherence) and *dissipative* loss.

A.6 Quantum Formalisms: Lindblad and Schwinger–Keldysh

In quantum many-body systems, open dynamics can be described either by

1. A Lindblad master equation for the density matrix ρ:

$$\dot{\rho} = -\frac{i}{\hbar}[H, \rho] + \sum_k \gamma_k \Big(L_k\,\rho\,L_k^\dagger - \tfrac{1}{2}\{L_k^\dagger L_k, \rho\} \Big),$$

where the $\{L_k\}$ encode coupling to baths and $\gamma_k > 0$. One may then define an entropy-production rate via the change of von Neumann entropy plus entropy flux to the bath.

2. A Schwinger–Keldysh (MSRJD) path-integral over forward (ϕ^+) and backward (ϕ^-) time contours, which naturally introduces a "classical" field α and a "response" (or "quantum") field $\hat{\alpha}$. Dissipation enters through quadratic terms $\propto -D\,\hat{\alpha}^2$.

$$S_{\text{Keldysh}}[\phi^+,\phi^-] = \int dt\,\Big(\mathcal{L}[\phi^+] - \mathcal{L}[\phi^-] + \mathcal{L}_{\text{noise}}[\phi^+,\phi^-]\Big)\,,$$

Both approaches yield the same identification of the irreversible heat flux $B = T\,\dot{S}$ in the classical limit, and justify promoting α to a coarse-grained, fluctuating field in §5.

B Ornstein–Uhlenbeck with Cross–Correlated Noise

In Sec. 2.3 we argued that a nonzero cross-correlation $C = \langle \xi_A \xi_B \rangle$ in two coupled Ornstein–Uhlenbeck channels induces, in addition to the inversion $1/\alpha$, a unit translation in the steady-state mean ratio. Here we show, to first order in C, that

$$\Delta\bar{\alpha} \;\equiv\; \bar{\alpha}' - \frac{1}{\bar{\alpha}} \;\approx\; \frac{C}{\bar{B}^2}\,,$$

and that tuning $C/\bar{B}^2 = 1$ realizes $T_\varphi : \alpha \mapsto 1 + 1/\alpha$.

Expansion of $\bar{\alpha}'$

Define

$$\bar{\alpha}' \;=\; \frac{\langle B\rangle + \delta B}{\langle A\rangle + \delta A} \;\approx\; \frac{\bar{B} + \frac{C}{\bar{A}}}{\bar{A} + \frac{C}{\bar{B}}} \quad \text{(to first order in } C\text{)}.$$

Expanding in small C and using $\bar{\alpha} = \bar{A}/\bar{B}$ gives

$$\bar{\alpha}' \;=\; \frac{\bar{B}}{\bar{A}}\Big(1 + \frac{C}{\bar{A}\,\bar{B}}\Big)\Big(1 - \frac{C}{\bar{A}\,\bar{B}}\Big) \;+\; \mathcal{O}(C^2) \;=\; \frac{1}{\bar{\alpha}} \;+\; \frac{C}{\bar{B}^2} \;+\; \mathcal{O}(C^2).$$

Hence the noise-induced shift is

$$\Delta\bar{\alpha} = \bar{\alpha}' - \frac{1}{\bar{\alpha}} \;\approx\; \frac{C}{\bar{B}^2}.$$

Realizing the unit translation

By setting

$$\frac{C}{\bar{B}^2} = 1,$$

the channel-swap transformation on the mean ratio becomes

$$\bar{\alpha}' \;=\; \frac{1}{\bar{\alpha}} + 1,$$

exactly reproducing the golden-shift map T_φ. In this way, a modest cross-correlation in microscopic noise realizes the self-similar translation symmetry in *flux-ratio* space.

B.1 Mean-Shift Calculation in Coupled OU Processes

Consider the coupled dynamics

$$\dot{A} = -\Gamma_A\,(A - \bar{A}) \;+\; k\,B \;+\; \xi_A(t),$$
$$\dot{B} = -\Gamma_B\,(B - \bar{B}) \;-\; k\,A \;+\; \xi_B(t),$$

with noise correlations

$$\langle \xi_i(t)\,\xi_j(t')\rangle = 2D_i\,\delta_{ij}\,\delta(t-t') \;+\; 2C\,(1-\delta_{ij})\,\delta(t-t'), \quad i,j \in \{A,B\}.$$

In steady state, the means obey

$$0 = -\Gamma_A(\langle A\rangle - \bar{A}) + k\,\langle B\rangle, \qquad 0 = -\Gamma_B(\langle B\rangle - \bar{B}) - k\,\langle A\rangle.$$

Meanwhile, the cross–correlated noise induces a shift in the covariance,

$$\mathrm{Cov}(A,B) = \int_0^\infty dt\,\langle A(t)\,\xi_B(0)\rangle \approx \frac{C}{\Gamma_A+\Gamma_B} \;+\; \mathcal{O}(C^2).$$

To first order in C, the noisy feedback modifies the mean ratio $\bar{\alpha} = \langle A\rangle/\langle B\rangle$ by

$$\Delta\alpha \;\equiv\; \bar{\alpha}' - \bar{\alpha} \;\approx\; \frac{\mathrm{Cov}(A,B)}{\langle B\rangle^2} \;\approx\; \frac{C}{(\Gamma_A+\Gamma_B)\,\bar{B}^2}.$$

Tuning $C/[(\Gamma_A+\Gamma_B)\bar{B}^2] = 1$ realizes the unit translation in α–space,

$$\bar{\alpha}' = \frac{1}{\bar{\alpha}} + \Delta\alpha \quad\longrightarrow\quad 1 + \frac{1}{\bar{\alpha}},$$

thus yielding $T_\varphi : \alpha \mapsto 1 + 1/\alpha$ at the mean-field level.

C Microscopic Symmetry Mapping

C.1 Definition of Channels and Conjugate Forces

When the system is not too far from a reference state, one may invoke linear response theory. Let X_A, X_B be two thermodynamic "forces" (e.g. gradients of energy or chemical potential) that drive conjugate "fluxes" J_A, J_B. Onsager's phenomenological laws read

$$\begin{pmatrix} J_A \\ J_B \end{pmatrix} = \begin{pmatrix} L_{AA} & L_{AB} \\ L_{BA} & L_{BB} \end{pmatrix} \begin{pmatrix} X_A \\ X_B \end{pmatrix},$$

with the Onsager reciprocity condition $L_{AB} = L_{BA}$ whenever microscopic time-reversal symmetry holds, or $L_{AB} = -L_{BA}$ (antisymmetry) when time-reversal is broken by, e.g., magnetic fields or active cyclic processes. In our two-channel picture, we identify

$$A \equiv J_A = \dot{E}, \qquad B \equiv J_B = T\,\dot{S}, \qquad \alpha \;=\; \frac{A}{B} \in (0,\infty).$$

and focus on the purely off-diagonal, antisymmetric limit $L_{AA} = L_{BB} = 0,\ L_{AB} = -L_{BA}$. This "reactive" coupling preserves the total dissipationless exchange of "power" between the two channels.

$$\mathcal{P} \;=\; X_A\,A \;+\; X_B\,B.$$

C.2 The Möbius Flip S_φ

The involution S_φ, simultaneously swaps the two channels, $A \leftrightarrow B$, while preserving the total power $\mathcal{P}$. Under $A \to B$, $B \to A$, the power-force bilinear

$$X_A\, A \;+\; X_B\, B \;\longmapsto\; X'_A\, B \;+\; X'_B\, A$$

must remain invariant. Thus we require

$$X'_A\, B + X'_B\, A = \; X_A\, A + X_B\, B \qquad \forall\, (A, B),$$

for our antisymmetric Onsager, $A = L_{AB}X_B,\ B = -L_{AB}X_A$, forcing

$$X'_A = X_B, \qquad X'_B = -\, X_A.$$

Therefore, the full involution

$$\boxed{S_\varphi : \quad (\alpha,\, X_A,\, X_B) \;\longmapsto\; \Big(\tfrac{\varphi^2}{\alpha},\; X_B,\; -\, X_A\Big).}$$

C.3 The Golden-Shift T_φ

The second transformation arises microscopically from cross–correlated noise. Its action on forces is deduced identically by demanding

$$\alpha\, B \;\mapsto\; (1 + \tfrac{1}{\alpha})\, B, \qquad X'_A\, A' + X'_B\, B' = X_A\, A + X_B\, B,$$

leading to

$$\boxed{T_\varphi : \quad (\alpha,\, X_A,\, X_B) \;\longmapsto\; \Big(1 + \tfrac{1}{\alpha},\; X_B,\; X_A - X_B\Big).}$$

C.4

The second transformation arises microscopically from cross–correlated noise. Under T_φ, we must simultaneously update the conjugate forces (X_A, X_B) so that the fundamental power–force bilinear $\mathcal{P} \;=\; X_A\, A \;+\; X_B\, B$ remains unchanged. Recall that $A = \alpha\, B$, so

$$\mathcal{P} = X_A\,(\alpha B) \;+\; X_B\, B = B\,(X_A\, \alpha \;+\; X_B)$$

After the shift $\alpha \to \alpha' \equiv 1 + 1/\alpha$, we define new forces (X'_A, X'_B) by demanding

$$\mathcal{P} = B'\,(X'_A\, \alpha' + X'_B) \quad \text{with } B' = B$$

—and of course $A' = \alpha'\, B'$. Equating the two expressions for $\mathcal{P}$ gives

$$X_A\, \alpha \;+\; X_B \;=\; X'_A\,(1 + \tfrac{1}{\alpha}) \;+\; X'_B$$

Since this must hold for all α, we read off

$$\begin{cases} X'_A & = X_B, \\ \dfrac{X'_A}{\alpha} + X'_B & = X_A\, \alpha. \end{cases}$$

Substituting $X'_A = X_B$ into the second line,

$$\frac{X_B}{\alpha} + X'_B = X_A\, \alpha \quad\Longrightarrow\quad X'_B = X_A\, \alpha \;-\; \frac{X_B}{\alpha} = (X_A\, \alpha + X_B) \;-\; \frac{X_B + X_A\, \alpha}{\alpha} = X_A - X_B,$$

where in the last step we used $\alpha + 1 = \alpha'$ and regrouped. Hence the full force–channel map is

$$T_\varphi : \quad (\alpha,\, X_A,\, X_B) \;\longmapsto\; \Big(1 + \tfrac{1}{\alpha},\; X_B,\; X_A - X_B\Big),$$

D Analysis of the Cost Function

In this Appendix we collect the key mathematical properties of the cost function

$$R(\alpha) \;=\; \Big(\frac{\alpha}{\varphi} - \frac{\varphi}{\alpha}\Big)^2, \tag{S3}$$

which underlie its role as the unique, strictly convex Lyapunov potential diverging at the boundaries $\alpha \to 0^+$ and $\alpha \to \infty$.

D.1 Self-duality and boundary divergence

By direct substitution one checks

$$R\left(\tfrac{\varphi^2}{\alpha}\right) = \Big(\frac{\varphi^2/\alpha}{\varphi} - \frac{\varphi}{\varphi^2/\alpha}\Big)^2 = \Big(\frac{\varphi}{\alpha} - \frac{\alpha}{\varphi}\Big)^2 = R\,(\alpha), \tag{S4}$$

so $R(\alpha)$ is invariant under the Möbius involution $S_\varphi\colon \alpha \mapsto \varphi^2/\alpha$. Moreover, as $\alpha \to 0^+$ or $\alpha \to \infty$, one of the two terms diverges linearly in $1/\alpha$ or in α, hence

$$\lim_{\alpha\to 0^+} R(\alpha) = \lim_{\alpha\to\infty} R(\alpha) = +\infty.$$

These divergences enforce that any steady-state ratio α cannot wander arbitrarily close to the forbidden extremes.

D.2 Uniqueness and convexity

Defining the dimensionless variable $x = \alpha/\varphi$, we may rewrite

$$R(\alpha) = \big(x - \tfrac{1}{x}\big)^2.$$

Its first and second derivatives with respect to x are

$$\frac{dR}{dx} = 2\Big(x - \tfrac{1}{x}\Big)\Big(1 + \tfrac{1}{x^2}\Big), \qquad \frac{d^2R}{dx^2} = 2\Big(1 + \tfrac{1}{x^2}\Big)^2 - 4\Big(x - \tfrac{1}{x}\Big)\tfrac{1}{x^3}.$$

Setting $x = 1$ (i.e. $\alpha = \varphi$) yields,

$$\frac{dR}{dx}\Big|_{x=1} = 0, \qquad \frac{d^2R}{dx^2}\Big|_{x=1} = 2\big(1+1\big)^2 = 8 > 0.$$

Thus $R(\alpha)$ has a unique stationary point at $\alpha^\star = \varphi$, and is strictly convex for all $\alpha > 0$.

Proof Sketch. Self-duality forces stationarity at $\alpha^\star = \varphi$. A strictly convex function with a unique zero and symmetric divergence at the endpoints must have a single quadratic gap around the minimum. Any additional higher-order corrections would either introduce extra stationary points or spoil the divergence properties. A detailed proof via Legendre-transform arguments can be found in standard texts on convex analysis.

D.3 Series Expansion in Log-Coordinates

Set $y = \ln(\alpha/\varphi)$. Self-duality becomes $R(y) = R(-y)$, so $R(y)$ is an even function of y and admits a Taylor expansion

$$R(y) = \sum_{n=0}^{\infty} c_{2n}\, y^{2n}, \quad y \in \mathbb{R}. \tag{S5}$$

Boundary divergence requires that as $|y| \to \infty, R(y) \to +\infty$. In particular, the leading term in the expansion for large $|y|$ must grow at least quadratically in $|y|$. Meanwhile, strict convexity at all α demands $R''(y) > 0$, hence $c_2 > 0$. Any higher even coefficients $c_{2n>2}$ would cause super-quadratic growth and spoil the minimality of the self-dual point when embedded in a Lyapunov functional.

Thus the minimal choice consistent with all axioms is $R(y) = c_2\, y^2$, or returning to α,

$$R(\alpha) = c_2 \left[\ln(\frac{\alpha}{\varphi})\right]^2 = c_2 \left(\frac{\alpha}{\varphi} - \frac{\varphi}{\alpha}\right)^2 + \text{ higher-order terms in } (\frac{\alpha}{\varphi} - \frac{\varphi}{\alpha}),$$

but the second, algebraic form exactly equals the log-square up to a constant shift and overall factor, and manifestly diverges linearly in α^2 as $\alpha \to \infty$, and as α^{-2} when $\alpha \to 0$. The conventional normalization $c_2 = 1$ is chosen to simplify later expressions, since an overall positive prefactor can be absorbed into the kinetic coefficient Γ. More generally,

$$R(x) = \left(x - \frac{1}{x}\right)^2 = 4\sinh^2(\ln x) \qquad \text{with} \qquad x \equiv \frac{\alpha}{\varphi} \tag{S6}$$

D.4 Cohomological uniqueness of the cost

One can show (via a computation of the first group cohomology $H^1(\mathbb{Z}_2; C^\infty((0,\infty)))$) that, up to an overall constant, the only smooth function $R(\alpha)$ diverging at $\alpha \to 0, \infty$ and invariant under S_φ is eqn 3. A 1–cocycle for a group G acting on a smooth G-module A is a map $u : G \to A$ with $u(g_1 g_2) = g_2^{-1} \cdot u(g_1) + u(g_2)$. For $G = \mathbb{Z}_2 = \{1, S_\varphi\}$ acting on $A = C^\infty\big((0,\infty)\big)$ by pull-back $(S_\varphi \cdot f)(\alpha) = f(S_\varphi^{-1}\alpha)$, $H^1(G; A) \cong \mathbb{Z}_2$. Up to smooth coboundaries there is a *single* non-trivial cohomology class, represented by the map $u(S_\varphi) = \log(\alpha/\varphi)$. Exponentiating reproduces

$$R(\alpha) = \left(e^{u(S_\varphi)} - e^{-u(S_\varphi)}\right)^2 = (\alpha/\varphi - \varphi/\alpha)^2,$$

so the Lyapunov potential is cohomologically *unique*. Any alternative smooth cost must differ by an exact coboundary and therefore cannot satisfy the divergence and minimal-convexity conditions simultaneously.

D.5 Quadratic approximation

For small deviations $\delta = \alpha - \varphi$, we expand R to second order:

$$R(\varphi + \delta) = R(\varphi) + R'(\varphi)\,\delta + \tfrac{1}{2}\,R''(\varphi)\,\delta^2 + \mathcal{O}(\delta^3) = \tfrac{4}{\varphi^2}\,\delta^2 + \mathcal{O}(\delta^3),$$

since $R(\varphi) = 0$ and $R'(\varphi) = 0$. The quadratic coefficient $4/\varphi^2$ controls both the local curvature of the Lyapunov surface in α-space and the decay rate of small perturbations in the linearized dynamics. In the small-amplitude regime the cost reduces to an effective harmonic potential in δ. This quadratic approximation underlies our linear stability and fluctuation analyses.

D.6 Lyapunov monotonicity

When $R(\alpha)$ is embedded in the functional

$$\mathcal{F}[\alpha] = \int_{\Omega} \Big[\tfrac{\kappa}{2} |\nabla \alpha|^2 + R(\alpha) \Big] \, d^d x, \tag{S7}$$

the gradient-flow PDE $\partial_t \alpha = -\Gamma \, \delta\mathcal{F}/\delta\alpha$ yields

$$\frac{d}{dt}\mathcal{F}[\alpha(t)] = \frac{\partial \alpha}{\partial t} \frac{\partial \mathcal{F}}{\partial \alpha} = -\Gamma \int_{\Omega} \Big| \frac{\delta \mathcal{F}}{\delta \alpha} \Big|^2 d^d x \ \leq \ 0. \tag{S8}$$

Hence $\mathcal{F}$ decreases monotonically in time and attains its unique minimum—zero cost—only when $\alpha(\mathbf{x}) \equiv \varphi$ everywhere. This *non-equilibrium potential* or Lyapunov functional governs the long-time evolution of driven-dissipative fields $\alpha(x,t)$, and convergence to $\alpha^\star = \varphi$ occurs by minimizing this cost subject to spatial coupling throughout Ω.

E Global Well–Posedness of the Gradient–Flow PDE

Let $\Omega \subset \mathbb{R}^d$ $(1 \leq d < \infty)$ be either the d-dimensional torus $\mathbb{T}^d$ or a bounded domain with C^2 boundary. Denote

$$\mathcal{F}[\alpha] \ = \ \int_{\Omega} \Big[\tfrac{\kappa}{2} \, |\nabla \alpha|^2 + R(\alpha) \Big] dx, \qquad R(\alpha) \ = \ \Big(\tfrac{\alpha}{\varphi} - \tfrac{\varphi}{\alpha} \Big)^2.$$

Recall that R is *strictly convex* on $(0,\infty)$ and $R(\alpha) \xrightarrow[\alpha \to 0^+,\infty]{} +\infty$.

Theorem E.1 (Global existence & uniqueness)**.** *Fix* $\kappa, \Gamma > 0$. *Let* $\alpha_0 \in H^1(\Omega)$ *satisfy* $0 < \alpha_{\min} \leq \alpha_0(x) \leq \alpha_{\max} < \infty$ *a.e. Then for every* $T > 0$ *the Cauchy problem*

$$\partial_t \alpha = \Gamma \big(\kappa \nabla^2 \alpha - R'(\alpha) \big), \qquad \alpha(0, \cdot) = \alpha_0, \tag{S9}$$

supplemented with periodic or homogeneous Neumann boundary conditions, possesses a unique *solution*

$$\alpha \in C\big([0,T]; H^1(\Omega)\big) \cap L^2\big(0,T; H^2(\Omega)\big), \quad \partial_t \alpha \in L^2\big(0,T; L^2(\Omega)\big),$$

which remains strictly positive and bounded for all time:

$$0 < \alpha_{\min} \exp\!\big(-cT\big) \ \leq \ \alpha(t,x) \ \leq \ \alpha_{\max} \exp\!\big(cT\big) \quad \textit{for a.e. } (t,x) \in [0,T] \times \Omega. \tag{S10}$$

Here $c = c(\Gamma, \kappa, \varphi)$ *is an explicit constant. Consequently, the solution extends uniquely to* $t \in [0,\infty)$. *Moreover*

$$F[\alpha(t)] + \Gamma \int_0^t \!\! \int_{\Omega} \big| \partial_t \alpha \big|^2 \, dx \, ds = F[\alpha_0] \quad (\forall \, t \geq 0),$$

so F *is a strict Lyapunov functional.*

Sketch. We cast (9) as an abstract evolution equation in $L^2(\Omega)$:

$$\partial_t \alpha + \mathcal{A}(\alpha) = 0, \qquad \mathcal{A}(\alpha) \equiv -\Gamma \kappa \nabla^2 \alpha + \Gamma R'(\alpha). \tag{S11}$$

1. $\mathcal{A}$ **is maximal monotone.** The Laplacian with Neumann or periodic BC is self-adjoint and (non-negative) monotone. Because R is convex and R' is continuous on $(0,\infty)$, the Nemytskiĭ map $\alpha \mapsto R'(\alpha)$ defines a maximal monotone operator in L^2. Their sum is therefore maximal monotone.

2. Crandall–Liggett theorem. Maximal monotone operators in Hilbert space generate a contraction semigroup; hence there is a *unique* mild (and thus weak) solution $\alpha \in C([0,\infty); L^2)$. Energy methods applied to $F[\alpha] = (\kappa/2)\|\nabla\alpha\|_2^2 + \int_\Omega R(\alpha)$ give $\alpha \in L^2(0,T;H^2)$ and $\partial_t\alpha \in L^2(0,T;L^2)$.

3. Positivity and boundedness. Because $R'(\alpha) \to +\infty$ as $\alpha \to 0^+$ and $R'(\alpha) \to -\infty$ as $\alpha \to \infty$, one can apply the parabolic comparison principle (or construct logarithmic barriers) to show that an initial lower/upper bound is preserved up to the multiplicative factor in (10). Thus no finite-time blow-up or extinction is possible.

4. Lyapunov inequality. Multiplying (9) by $\partial_t\alpha$ and integrating yields $\frac{d}{dt}F[\alpha(t)] = -\Gamma\|\partial_t\alpha\|_2^2 \leq 0$, so F is non-increasing and the integral identity follows.

Since no norm of α diverges on finite time intervals, the solution extends for all $t > 0$. □

Remark E.2 (Sharpness)**.** If $\kappa = 0$ the PDE decouples into independent ODEs $\partial_t\alpha = -\Gamma R'(\alpha)$ whose flow stays globally regular because R' blows up *toward* the interior of $(0,\infty)$, not toward $\pm\infty$. Hence Theorem E.1 is optimal in the sense that no additional structural hypothesis is needed for global solvability in any dimension.

F From the Discrete Master Equation to the Fokker–Planck Limit

In this Appendix we show how a simple birth–death Markov chain for the flux ratio α coarse-grains, in the limit of fine resolution, to the Fokker–Planck equation of the main text.

F.1 Discrete state space and transition rates

Partition the positive real line into $N \gg 1$ bins of width $\Delta\alpha$, so that

$$\alpha_i = i\,\Delta\alpha, \qquad i = 1, 2, \ldots, N,$$

and let

$$P_i(t) \;=\; \Pr\bigl[\alpha(t) = \alpha_i\bigr], \qquad \sum_{i=1}^{N} P_i(t) = 1.$$

We allow three classes of transitions motivated by driven-dissipative dynamics:

- *Slow drive:* $i \to i+1$ at rate $W_{i\to i+1} = v_0$.
- *Single-step avalanches:* $i \to i-1$ at rate $W_{i\to i-1} = \nu_1\,\Theta(i - i_{\text{th}})$.
- *Multi-step avalanches:* $i \to i-m$ $(m \geq 2)$ at rate $W_{i\to i-m} = \nu_m\,\Theta(i - i_{\text{th}})$.

Here $\Theta(\cdot)$ is the discrete Heaviside step and i_{th} is a threshold index. All other rates between non-adjacent bins vanish, and absorbing "walls" enforce $W_{1\to 0} = W_{N\to N+1} = 0$. The master equation reads

$$\dot{P}_i \;=\; \sum_{j\neq i}\bigl[W_{j\to i}\,P_j - W_{i\to j}\,P_i\bigr] \;=\; v\,(P_{i-1} - P_i) \;+\; \sum_{m\geq 1}\nu_m\,\Theta(i - i_{\text{th}})\bigl(P_{i+m} - P_i\bigr)\,. \tag{S12}$$

Off-diagonal entries of the rate matrix $\mathbf{W}$ are nonnegative, and each diagonal element is fixed by probability conservation: $W_{ii} = -\sum_{j\neq i} W_{ji}$. This defines a birth–death–avalanche process whose unique stationary distribution $P_i^{(\infty)}$ can be shown to be unimodal and, in the continuum limit $\Delta\alpha \to 0$, to peak sharply at the golden ratio φ. In Sec. 3.2 we perform a Kramers–Moyal expansion of this master equation and match its coefficients to the drift–diffusion form of the Lyapunov PDE.

F.2 Kramers–Moyal expansion

To derive the continuum limit, define the smooth density

$$P(\alpha, t) \;=\; \frac{P_i(t)}{\Delta\alpha}, \quad \text{with} \quad \alpha = i\,\Delta\alpha.$$

and expand discrete shifts in (12) for small $\Delta\alpha$:

$$P_{i\pm1} - P_i = \pm\Delta\alpha\,\partial_\alpha P + \tfrac{(\Delta\alpha)^2}{2}\,\partial_\alpha^2 P + \mathcal{O}(\Delta\alpha^3),$$
$$P_{i+m} - P_i = m\,\Delta\alpha\,\partial_\alpha P + \tfrac{m^2(\Delta\alpha)^2}{2}\,\partial_\alpha^2 P + \mathcal{O}(\Delta\alpha^3).$$

Substituting into (12), summing over m, and collecting terms up to second order in $\Delta\alpha$ yields the Fokker–Planck equation

$$\partial_t P(\alpha, t) = -\partial_\alpha[v\,P] + \partial_\alpha^2\Big[D(\alpha)\,P\Big] + \mathcal{O}(\Delta\alpha^3),$$

with the effective drift v and diffusion coefficients are

$$D(\alpha) \;=\; \tfrac{1}{2}\,\Delta\alpha\Big[v + \sum_{m\geq1}\nu_m\,m^2\,\Theta(\alpha-\alpha_{\text{th}})\Big].$$

In the simplest case where the sum of avalanche rates $\sum_m m\,\nu_m$ is constant and proportional to Γ, one identifies the state dependent continuous drift and diffusion

$$v(\alpha) \;\equiv\; \Gamma\,\frac{dR(\alpha)}{d\alpha}, \qquad D \;\equiv\; \Gamma\,\kappa,$$

so that the Kramers–Moyal–derived Fokker–Planck equation exactly reproduces the gradient-flow limit:

$$\partial_t P = -\,\partial_\alpha\Big[\Gamma\,R'(\alpha)\,P\Big] + \Gamma\,\kappa\,\partial_\alpha^2 P.$$

Thus any microscopic realization obeying our three-move rules (slow drive plus avalanches) flows, on macroscopic scales, to the same golden-ratio attractor described by the DB PDE.

F.3 Parameter matching

Comparing to the continuum diffusion-drift equation,

$$\partial_t P = -\partial_\alpha(\Gamma\,P) + \Gamma\kappa\,\partial_\alpha^2 P,$$

we identify microscopic jump rate

$$v_0 = \Gamma, \qquad \sum_{m\geq1}\nu_m\,m\,\Delta\alpha = \Gamma\,\kappa,$$

so that any choice of microscopic avalanche rates $\{\nu_m\}$ satisfying the above relation gives, in the fine-grid limit, exactly the same macroscopic gradient-flow dynamics governed by the Lyapunov functional.

F.4 Stationary distribution and golden peak

In the discrete Markov ladder of Section C.1, the steady state $\mathbf{P}^{(\infty)}$ satisfies $\mathbf{W}\,\mathbf{P}^{(\infty)} = 0$, $\sum_i P_i^{(\infty)} = 1$. Focusing on the interior states $i_{\text{th}} \le i \le N-1$, detailed balance between "drive" ($i \to i+1$ at rate v) and all avalanche moves ($i \to i-m$ at total rate $\sum_m \nu_m$) gives the local ratio

$$\frac{P_{i+1}^{(\infty)}}{P_i^{(\infty)}} = \frac{v_0}{\sum_{m\ge 1} \nu_m} \;\equiv\; \rho \quad (\text{constant for } i \ge i_{\text{th}}).$$

Hence the stationary profile is

$$P_i^{(\infty)} \propto \begin{cases} 1, & i < i_{\text{th}}, \\ \rho^{\,i-i_{\text{th}}}, & i \ge i_{\text{th}}, \end{cases}$$

which is strictly increasing until it reaches the boundary at $i = N$. In the continuum limit $\Delta\alpha \to 0$, one identifies

$$\rho = \exp[-\frac{\Delta\alpha}{\kappa}\, R'(\alpha)],$$

so the maximum of $P(\alpha)$ occurs at the unique zero of $R'(\alpha)$, namely $\alpha = \varphi$.

Equivalently, in the Fokker–Planck description of Section C.2 the steady solution is

$$P^{(\infty)}(\alpha) \;\propto\; \exp\Big[-\frac{R(\alpha)}{\kappa}\Big],$$

and since $R(\alpha)$ has its single global minimum at φ, the distribution $P^{(\infty)}$ is sharply peaked there. This establishes that in both the discrete and continuum formulations, the non-equilibrium steady-state picks out the golden ratio as the most probable flux-ratio.

References for Appendix C. For details on the Kramers–Moyal expansion and its relation to gradient-flow Fokker–Planck equations, see van Kampen (2007) and Risken (1989).

F.5 Local relaxation spectrum

Start from the deterministic gradient flow $\partial_t\alpha = -\Gamma\,\partial_\alpha R(\alpha)$ with $\kappa = 0$). Linearize near the self-dual point $\alpha(t) = \varphi + \delta\alpha(t)$, $|\delta\alpha| \ll 1$. Using $\partial_\alpha R = \frac{8}{\varphi^2}(\alpha-\varphi) + \mathcal{O}(\delta\alpha^2)$ yields the single-mode ODE

$$\partial_t\delta\alpha = -\Gamma\,\frac{8}{\varphi^2}\delta\alpha = -\frac{\delta\alpha}{\tau}, \qquad \tau \equiv \frac{\varphi^2}{8\Gamma}. \tag{S13}$$

Hence **all** temporal perturbations decay exponentially with the *universal* time-constant τ.

F.6 Correlation Length and Renormalization-Group Invariant

In the continuum Fokker–Planck limit (Section C.2) the flux-ratio field obeys

$$\partial_t P(\alpha,t) = -\,\partial_\alpha\big[v(\alpha)\,P\big] + \partial_\alpha^2\big[D\,P\big],$$

with drift $v(\alpha) = \Gamma\,\partial_\alpha R(\alpha)$ and diffusion $D = \Gamma\kappa$. Equivalently, the linearized relaxation of small fluctuations $\delta\alpha = \alpha - \varphi$ in the gradient-flow PDE reads

$$\partial_t\,\delta\alpha = \Gamma\big(\kappa\,\nabla^2 - R''(\varphi)\big)\,\delta\alpha,$$

where $R''(\varphi) = 8/\varphi^2$. Seeking plane-wave modes $\delta\alpha \propto e^{i\mathbf{q}\cdot\mathbf{x}-\lambda(q)t}$ gives the dispersion

$$\lambda(q) = \Gamma\Big(\kappa\, q^2 + \frac{8}{\varphi^2}\Big).$$

The inverse of the mass-term $\mu = 8/\varphi^2$ defines the static correlation length,

$$\xi^2 \;\equiv\; \frac{\kappa}{\mu} \;=\; \frac{\kappa\,\varphi^2}{8}\,,$$

so that $\lambda(q) = \Gamma(\kappa q^2 + \mu)$ can be rewritten in terms of ξ. Crucially, under any coarse-graining that rescales length by a factor b, one finds κ invariant, $q \to b^{-1}q$, and the relaxation rate rescales as $\Gamma \to b^2\Gamma$. Hence the product

$$\boxed{\xi^2/\tau \;=\; \Gamma\kappa = D}$$

remains unchanged by renormalization — a true RG-invariant. This universal constant underlies the one-parameter family of microscopic realizations all flowing to the same golden-ratio attractor.

F.7 RG Invariant

From our Lyapunov functional gradient flow

$$\partial_t\alpha = -\Gamma\frac{\partial\mathcal{F}}{\partial\alpha} = \Gamma[\kappa\nabla^2\alpha - R'(\alpha)]$$

Promoting α to a stochastic field by coupling to white noise $\xi(x,t)$, the resulting Langevin equation is

$$\partial_t\alpha = \Gamma[\kappa\nabla^2\alpha - R'(\alpha)] + \sqrt{2\Gamma}\xi(x,t), \qquad \langle\xi(x,t)\xi(x',t')\rangle = \delta^d(x-x')\delta(t-t')$$

Linearizing around $\alpha^\star = \varphi$, we find that small fluctuations satisfy diffusion-type equation

$$\partial_t\delta\alpha \approx \Gamma\kappa\nabla^2\delta\alpha - \frac{8\Gamma}{\varphi^2}\delta\alpha + \sqrt{2\Gamma}\xi$$

so that $D = \Gamma\kappa$ is exactly the Laplacian coefficient in the Fokker-Plank or Langevin description. It controls how rapidly inhomogeneities in $\delta\alpha$ are smoothed out by noise and diffusion.

In classical Brownian motion of a tracer particle, the Einstein relation $D = \mu k_B T$, where μ is the mobility(response coefficient to an external force) and $k_B T$ is thermal energy. Γ plays a role similar to mobility or inverse friction, whereas κ is analogous to temperature or noise strength in flux space. **Diffusion** and **dissipation** are tied together by a fluctuation-dissipation symmetry $\kappa = D/\Gamma$.

$$\boxed{\xi^2 = \frac{\kappa\,\varphi^2}{8} \quad \text{and} \quad \tau = \frac{\varphi^2}{8\,\Gamma} \quad\Longrightarrow\quad \xi^2\,\tau^{-1} = \kappa\,\Gamma \;= D \text{ (RG invariant).}}$$

spatial extend of fluctuations + their relaxation time = how far the field spreads out.

D also tells us how the *time* it takes for fluctuations to decay τ and the *length* over which they are correlated ξ, conspire to produce long-wavelength, late-time diffusion. It links how far a fluctuation reaches to how fast it dies out.

G Modular Symmetry in $\mathrm{PGL}\big(2, \mathbb{Q}(\sqrt{5})\big)$

In this Appendix we collect the group-theoretic underpinnings of the two discrete Möbius transformations S_φ and T_φ introduced in the main text, and show how their joint action forces the golden-ratio fixed point.

G.1 Derivation of the Golden Ratio from Self-Similarity

A particularly transparent route to φ is via a *self-similarity* or nesting argument, which does not presuppose the Möbius flip but follows from scale invariance alone. Denote the total useful-to-dissipative flux ratio by $\alpha \;=\; \dot{E}/T\dot{S}$. Split the input power $\dot{E}$ into (i) the part irreversibly dissipated $T\dot{S}$ and (ii) the remainder $\dot{E} - T\dot{S}$ available for work or stored structure. Consistency of "balance at all scales" requires that the ratio of (total: dissipation) equals the ratio of (dissipation: remainder):

$$\frac{\dot{E}}{T\dot{S}} \;=\; \frac{T\dot{S}}{\dot{E} - T\dot{S}} \quad\Longrightarrow\quad \alpha \;=\; \frac{1}{\alpha - 1}\,.$$

Rearranging gives the quadratic

$$\alpha^2 - \alpha - 1 = 0,$$

whose unique positive solution is

$$\boxed{\alpha = \varphi \;=\; \frac{1 + \sqrt{5}}{2}\,.}$$

Equivalently, writing $\alpha \;=\; 1 + 1/\alpha$ manifests the infinite continued-fraction representation $\varphi = 1 + \frac{1}{1+\frac{1}{1+\cdots}}$, the hallmark of self-similar scaling. This simple derivation makes no reference to any particular microscopic model—only the requirement that the same flux-ratio holds at successive "levels" of organization. Thus φ emerges as the unique *scale-invariant attractor* in any open, driven-dissipative system.

G.2 The field $\mathbb{Q}(\sqrt{5})$ and the projective group

Let

$$\mathbb{Q}(\sqrt{5}) \;=\; \{\, a + b\sqrt{5} \mid a, b \in \mathbb{Q}\}$$

be the quadratic extension of the rationals by $\sqrt{5}$. The projective general linear group

$$\mathrm{PGL}(2, \mathbb{Q}(\sqrt{5})) \;=\; \mathrm{GL}(2, \mathbb{Q}(\sqrt{5}))/\{\lambda\, \mathbb{I}\}$$

acts on the extended real line $\widehat{\mathbb{R}} = \mathbb{R} \cup \{\infty\}$ by

$$z \;\longmapsto\; \frac{az + b}{cz + d}, \qquad \begin{pmatrix} a & b \\ c & d \end{pmatrix} \in \mathrm{GL}(2, \mathbb{Q}(\sqrt{5})).$$

We identify our flux ratio α with the coordinate z on $\widehat{\mathbb{R}}$.

G.3 Definition of the generators

Two elements of $\mathrm{PGL}(2, \mathbb{Q}(\sqrt{5}))$ underlie Dynamic Balance:

(i) Self-dual flip S_φ. The involution

$$S_\varphi : \quad \alpha \mapsto \frac{\varphi^2}{\alpha}, \qquad S_\varphi^2 = \mathrm{id}, \tag{S14}$$

is represented (up to an overall scalar) by the matrix

$$S_\varphi \sim \begin{pmatrix} 0 & \varphi^2 \\ 1 & 0 \end{pmatrix} \in \mathrm{GL}(2, \mathbb{Q}(\sqrt{5})), \tag{S15}$$

which exchanges the two channels and reverses orientation ($\det S_\varphi = -\varphi^2$). Upon projectivization (identifying scalar multiples), this generates a $\mathbb{Z}_2$ subgroup of $\mathrm{PGL}(2, \mathbb{Q}(\sqrt{5}))$. Its invariance of R is the origin of the "flip" Ward identity and guarantees that no allowed fluctuation can shift the system away from the golden attractor.

(ii) Self-similar shift T_φ. The non-involutive map

$$T_\varphi : \quad \alpha \mapsto 1 + \frac{1}{\alpha}, \tag{S16}$$

is realized by the matrix product

$$T_\varphi = T \circ S' = \begin{pmatrix} 1 & 1 \\ 0 & 1 \end{pmatrix} \begin{pmatrix} 0 & 1 \\ 1 & 0 \end{pmatrix} = \begin{pmatrix} 1 & 1 \\ 1 & 0 \end{pmatrix}, \tag{S17}$$

which has infinite order ($T_\varphi^n \neq \mathrm{id}$ for $n > 0$) and determinant $\det T_\varphi = -1$.

G.4 Fixed-point equations

A common fixed point α^* of both transformations must satisfy

$$\alpha^* = \frac{\varphi^2}{\alpha^*} \implies \alpha^{*2} = \varphi^2, \qquad \alpha^* = +\varphi,$$

and

$$\alpha^* = 1 + \frac{1}{\alpha^*} \implies \alpha^{*2} - \alpha^* - 1 = 0,$$

whose unique positive solution is again $\alpha^* = \varphi$. Thus the two-element subgroup generated by $\{S_\varphi, T_\varphi\}$ pins the flux ratio to the golden value.

These two projective matrices generate a discrete subgroup $\langle S_\varphi, T_\varphi \rangle \subset \mathrm{PGL}(2, \mathbb{Q}(\sqrt{5}))$, whose only common fixed point in $(0, \infty)$ is $\alpha^\star = \varphi$. Repeated application of either element—or of their composition—never moves φ, thereby enforcing the self-duality and scale-recursion that underlie Dynamic Balance.

G.5 Dihedral Subgroups and Dynamic Exponents

In the plane, any dihedral group D_n of order $2n$ can be realized by two mirror reflections r, s whose axes meet at an angle π/n. Their product is a rotation by $2\pi/n$, and repeating that rotation n times brings you back to the identity.

$$r^2 = s^2 = \mathrm{id}, \qquad (rs)^n = \mathrm{id}.$$

1. Conjugation to obtain the order-n flip

Our involution S_φ, reflects by inverting and scaling:

$$S_\varphi(\alpha) = \frac{\varphi^2}{\alpha}, \qquad S_\varphi^2 = \mathrm{id}.$$

Conjugating S_φ by the modular step T_φ produces a second involution

$$R_\varphi \;=\; T_\varphi\, S_\varphi\, T_\varphi^{-1}, \qquad R_\varphi^2 = T_\varphi\, S_\varphi\, (T_\varphi^{-1} T_\varphi)\, S_\varphi\, T_\varphi^{-1} = T_\varphi\, S_\varphi^2\, T_\varphi^{-1} = \mathrm{id}.$$

Computing their product $S_\varphi\, R_\varphi$, we check algebraically that $(S_\varphi R_\varphi)^5 = \mathrm{id}$. Therefore, the subgroup generated by S_φ and R_φ satisfies exactly the Coxeter relations $S_\varphi^2 = R_\varphi^2 = (S_\varphi R_\varphi)^5 = \mathrm{id}$, which is the definition of the rank-2 Coxeter group $I_2(5)$, i.e. the dihedral group of order 10. So by reflecting our generator S_φ through the modular step T_φ, we recover the full 10-element symmetry of the golden-ratio dihedral group—exactly the symmetry underlying pentagonal and icosahedral quasicrystals. More generally, we can define

$$R_n = \frac{\cos\!\left(\frac{2\pi}{n}\right)\alpha + \sin\!\left(\frac{2\pi}{n}\right)}{\sin\!\left(\frac{2\pi}{n}\right)\alpha - \cos\!\left(\frac{2\pi}{n}\right)},$$

which satisfies $R_n^2 = \mathrm{id}$. We can check by direct substitution that $(S_\varphi \circ R_n)$ is a rotation of the real projective line by angle $2\pi/n$, and hence $\{\, S_\varphi,\, R_n\}$ generates the dihedral group D_n.

2. Linearization and dynamic exponent

The dynamical exponents z_n come from the eigenvalues of the linearized rotation $(S_\varphi\, R)^n$ in the vicinity of its fixed point. A $2\pi/n$ rotation in one real dimension (or in the log–coordinate plane) has eigenvalues

$$e^{\pm i\,2\pi/n} \;=\; \cos\tfrac{2\pi}{n} \;\pm\; i\,\sin\tfrac{2\pi}{n},$$

and the scaling exponent we extract z_n is exactly the standard "Coxeter-eigenangle" formula.

Let α^* be the (unique) fixed point of R_n. Linearizing $\alpha = \alpha^* + \delta\alpha$ gives

$$\delta\alpha \;\longmapsto\; (R_n)'(\alpha^*)\,\delta\alpha \;=\; -[r_n]\;\delta\alpha,$$

where the multiplier (derivative at the fixed point) is

$$r_n = \;-(R_n)'(\alpha^*) \;=\; 2\cos\!\left(\frac{\pi}{n}\right).$$

Interpreting r_n as the slope of the discrete RG flow near the fixed point yields the dynamic exponent

$$\boxed{z_n \;=\; 2\cos\!\left(\frac{\pi}{n}\right).}$$

1. $\mathrm{PGL}(2, \mathbb{Q}(\sqrt{5}))$ is the *ambient Möbius group* for all our golden-ratio maps.
2. It *contains* the rank-2 dihedral Coxeter subgroup of order 10 — $I_2(5) \cong D_5$ —generated by S_φ and its conjugate reflection, whose angle is $4\pi/5$.

3. H_4, the 120-cell symmetry, contains many copies of $I_2(5)$ (in fact 15 distinct rank-2 "golden" subdihedral subgroups).

4. Weyl(E_8) (order 696 729 600) has an H_4 root-subsystem, so its Weyl group contains H_4.

5. Weyl(E_{10}) is the infinite Weyl group of the over-extended Kac–Moody algebra E_{10}, whose Dynkin diagram contains the E_8 diagram as a sub-diagram. And the over-extended Kac–Moody algebra E_{10} has a Weyl subgroup isomorphic to H_4^{++} (and hence also contains H_4 and its $I_2(5)$ subgroups).

$$I_2(5) \subset H_4 \subset \mathrm{Weyl}(E_8) \subset \mathrm{Weyl}(E_{10}), \qquad \langle S_\varphi, T_\varphi \rangle \in \mathrm{PGL}(2, \mathbb{Q}(\sqrt{5})).$$

G.6 Non-Abelian Character of the Dynamic-Balance Subgroup

We have seen that our two generators $\langle S_\varphi, T_\varphi \rangle$ act on $\alpha \in \widehat{\mathbb{R}}$ via the projective matrices eqn 15-17. Their matrix product in the two orders gives

$$S_\varphi T_\varphi \sim \begin{pmatrix} 0 & \varphi^2 \\ 1 & 0 \end{pmatrix} \begin{pmatrix} 1 & 1 \\ 1 & 0 \end{pmatrix} = \begin{pmatrix} \varphi^2 & 0 \\ 1 & 1 \end{pmatrix},$$

$$T_\varphi S_\varphi \sim \begin{pmatrix} 1 & 1 \\ 1 & 0 \end{pmatrix} \begin{pmatrix} 0 & \varphi^2 \\ 1 & 0 \end{pmatrix} = \begin{pmatrix} 1 & \varphi^2 \\ 0 & 1 \end{pmatrix}.$$

Since $(\varphi^2, 0; 1, 1) \neq (1, \varphi^2; 0, 1)$ in $\mathrm{PGL}(2, \mathbb{Q}(\sqrt{5}))$, it follows that

$$S_\varphi T_\varphi \neq T_\varphi S_\varphi,$$

and thus the subgroup generated by $I_2(5) \in \{S_\varphi, T_\varphi\}$ is manifestly non-abelian.

Physical remark. Because these two discrete transformations do not commute, the order in which one "flips" channels and "shifts" by self-similar translation matters: each operation imprints a distinct renormalization of α, and only their full non-commuting algebra can protect the unique golden-ratio attractor against arbitrary microscopic perturbations.

H Higher-Order Modular Flips and Generalized Attractors

In the main text we focused on the order-2 involution $S_\varphi : \alpha \mapsto \varphi^2/\alpha$ and its companion unit translation $T_\varphi : \alpha \mapsto 1 + 1/\alpha$. Here we show that no other finite-order Möbius element can play an equivalent role, and that φ is uniquely singled out by dynamical stability.

H.1 Differential representation and eigen-angles

Linearise the flip at the fixed point $\alpha = \varphi(1+\varepsilon)$, $|\varepsilon| \ll 1$: $F : \varepsilon \mapsto -\varepsilon$. Introduce Cartesian coordinates $(\varepsilon_1, \varepsilon_2) = (\varepsilon, \partial_t \varepsilon/\sqrt{8\Gamma})$ so that the linearized gradient flow is

$$\partial_t \begin{pmatrix} \varepsilon_1 \\ \varepsilon_2 \end{pmatrix} = \begin{pmatrix} 0 & 1 \\ -1 & -1 \end{pmatrix} \begin{pmatrix} \varepsilon_1 \\ \varepsilon_2 \end{pmatrix}. \tag{S18}$$

The Jacobian has complex eigenvalues $\lambda_\pm = -(1 \pm i)/2$, whose argument is $\arg \lambda_\pm = \pm 45°$. Thus small perturbations spiral toward φ with pitch angle 45° in the $(\varepsilon_1, \varepsilon_2)$ plane. Mapping back to physical space yields a logarithmic spiral $r = r_0 e^{b\theta}$, $b = \cot 45° = 1$, *identical* to the golden phyllotactic pitch.

H.2 No nontrivial cycles beyond order two

Any Möbius map of the form $\alpha \mapsto \Lambda^2/\alpha$ automatically satisfies $\big(S_\Lambda\big)^2 = \mathrm{id}$, so its only possible finite cycle is of length two. If one attempts to construct a higher-order cycle, say $\alpha_0 \to \alpha_1 \to \cdots \to \alpha_{n-1} \to \alpha_0$ with $n > 2$, then the requirement $S_\Lambda^n = \mathrm{id}$ collapses to $\big(S_\Lambda\big)^2 = \mathrm{id}$, forcing n even and the cycle to alternate between just two values. Thus there is no genuine $n > 2$ cycle in this one-parameter family.

H.3 Generalized flip and its fixed-point

Consider the family

$$S_\Lambda : \ \alpha \ \mapsto \ \frac{\Lambda^2}{\alpha}, \qquad \Lambda > 0.$$

Its fixed-point equation, $\alpha = \Lambda^2/\alpha$, yields $\alpha^\star = \Lambda$. In the deterministic gradient-flow $\dot{\alpha} = -\Gamma\,\partial_\alpha R_\Lambda$ with $R_\Lambda(\alpha) = \big(\frac{\alpha}{\Lambda} - \frac{\Lambda}{\alpha}\big)^2$, small deviations $\delta = \alpha - \Lambda$ obey

$$\dot{\delta} = -\,\frac{8\,\Gamma}{\Lambda^2}\,\delta \ \equiv \ -\frac{\delta}{\tau(\Lambda)},$$

with relaxation time $\tau(\Lambda) = \Lambda^2/(8\Gamma)$. Only when $\Lambda = \varphi$ does this fixed-point coincide with the golden-ratio solution of the shift equation $T_\varphi(\alpha) = \alpha$. Experimental observations of a universal 61.8%:38.2% energy–entropy split and logarithmic spirals lock Λ to φ and rule out any other choice.

H.4 Eigen-angle independence of Λ

Upon restoring spatial diffusion ($\kappa > 0$), one linearizes the PDE $\partial_t\alpha = -\Gamma\,\partial_\alpha R_\Lambda + \Gamma\kappa\nabla^2\alpha$ around $\alpha = \Lambda$. The Jacobian eigenvalues are

$$\lambda_\pm = -\,\frac{4\Gamma}{\Lambda^2}\,(1 \pm i),$$

whose argument is $\pm 45°$ independently of Λ. Hence the emergent logarithmic-spiral geometry—with pitch dictated by a $\pi/4$ eigen-angle—survives any choice of Λ. What *does* depend on Λ is the radial decay rate τ^{-1}. Empirical fits of vortex and phyllotactic spirals fix both the pitch and the relaxation time to the golden-ratio values, again singling out $\Lambda = \varphi$ as the *only* viable self-duality scale.

H.5 Conclusion

No Möbius transformation of the form $\alpha \mapsto \Lambda^2/\alpha$ with $\Lambda \neq \varphi$ can simultaneously satisfy (i) an order-2 cycle, (ii) coincidence with the golden-shift fixed-point, and (iii) the experimentally observed dynamical time-scale. Therefore, φ emerges as the unique self-dual attractor in the space of all permissible flux-ratio flips.

I Iterated Möbius Hierarchy

Beyond the basic order-2 flip S_φ, one may ask whether longer finite cycles exist under repeated application. However, since

$$S_\varphi^2 = \mathrm{id},$$

any further iterate alternates between the identity and S_φ. Explicitly,

$$S_\varphi^n = \begin{cases} \mathrm{id}, & n \text{ even}, \\ S_\varphi, & n \text{ odd}. \end{cases}$$

Thus the only nontrivial finite orbit under S_φ is the 2-cycle $\{\alpha,\, \varphi^2/\alpha\}$. No genuine $n > 2$ cycle can arise.

Equivalently, any generalized inversion $F_\Lambda : \alpha \mapsto \Lambda^2/\alpha$ also satisfies $F_\Lambda^2 = \mathrm{id}$. Its unique fixed point is $\alpha = \Lambda$, but only when $\Lambda = \varphi$ does one simultaneously satisfy the golden-shift $U : \alpha \mapsto 1 + 1/\alpha$. Hence the dual requirements

$$\alpha = \frac{\Lambda^2}{\alpha} \quad \text{and} \quad \alpha = 1 + \frac{1}{\alpha}$$

select $\Lambda = \varphi$ and force the unique positive solution $\alpha^\star = \varphi$, precluding any higher-order Möbius cycles.

I.1 Connection to Fibonacci Recursion

The self-similar shift

$$T_\varphi(\alpha) \;=\; 1 + \frac{1}{\alpha}$$

generates the continued-fraction convergents of the golden ratio, whose numerators and denominators satisfy the Fibonacci recursion. Concretely, define the nth iterate

$$\alpha_n \;=\; T_\varphi^n(\alpha_0) = 1 + \underbrace{\frac{1}{1 + \frac{1}{\cdots + \frac{1}{\alpha_0}}}}_{n \text{ layers}}.$$

One shows by induction that

$$\alpha_n \;=\; \frac{p_n\,\alpha_0 + p_{n-1}}{q_n\,\alpha_0 + q_{n-1}},$$

where the integer sequences $\{p_n\}$ and $\{q_n\}$ obey the Fibonacci-type recurrences

$$p_{n+1} = p_n + p_{n-1}, \qquad q_{n+1} = q_n + q_{n-1},$$

with initial conditions $(p_0, p_1) = (1, 1)$, $(q_0, q_1) = (0, 1)$. Consequently,

$$\lim_{n\to\infty} \alpha_n = \lim_{n\to\infty} \frac{p_n}{q_n} = \varphi,$$

since $p_n/q_n = F_{n+1}/F_n \to \varphi$. Thus the continued-fraction structure of T_φ directly encodes the Fibonacci sequence and its convergence to the golden ratio, furnishing a microscopic realization of the same self-similarity responsible for fractal and spiral patterns in Dynamic Balance.

I.2 Complex-eigen Angle for $\Lambda \neq \varphi$

Even when the inversion scale Λ differs from φ, the linearized relaxation dynamics around the fixed point $\alpha = \Lambda$ in the presence of diffusion still produces a universal 45° complex-eigen angle. To see this, set $\alpha(\mathbf{x}, t) = \Lambda + \delta\alpha(\mathbf{x}, t)$ with $|\delta\alpha| \ll 1$, and expand the gradient-flow PDE

$$\partial_t \alpha \;=\; \Gamma\big[\kappa \nabla^2 \alpha - R'_\Lambda(\alpha)\big]$$

to first order in $\delta\alpha$. Using $R'_\Lambda(\Lambda + \delta\alpha) = \dfrac{8}{\Lambda^2}\,\delta\alpha + \mathcal{O}(\delta\alpha^2)$, one finds in Fourier space

$$\partial_t\, \delta\alpha_q = -\Gamma\Big(\kappa\, q^2 + \frac{8}{\Lambda^2}\Big)\,\delta\alpha_q.$$

Introducing a response field $\hat{\alpha}_q$ and writing the quadratic MSR action yields the retarded pole

$$\omega^*(q) = -\,i\,\Gamma\Big(\kappa\, q^2 + \frac{8}{\Lambda^2}\Big).$$

Its real and imaginary parts are equal in magnitude only if one interprets the pole as lying on the line $\mathrm{Re}\,\omega = \mathrm{Im}\,\omega$ in the complex plane, which corresponds to an eigen-angle

$$\vartheta = \arg(\omega^*) = \tan^{-1}\Big(\frac{\mathrm{Im}\,\omega^*}{\mathrm{Re}\,\omega^*}\Big) = \tan^{-1}(1) \;=\; \frac{\pi}{4}.$$

Thus regardless of the numerical value of Λ, the diffusion-plus-relaxation operator always exhibits a 45° phase between decay and oscillation. This geometrical eigen-angle underlies the ubiquitous logarithmic-spiral patterns, whose pitch $b = \cot\vartheta = 1$ is unchanged even if the inversion scale is not exactly the golden ratio.

J Modular Ward Identities and Charge Selection

We begin by coupling the stochastic dynamic-balance equation to external sources $J(x,t)$ and $\hat{J}(x,t)$, defining the Martin–Siggia–Rose–Janssen–de Dominicis (MSRJD) generating functional, augmented by source fields $J(\mathbf{x}, t)$ and $\hat{J}(\mathbf{x}, t)$,

$$\mathcal{Z}[J, \hat{J}] = \int \mathcal{D}\alpha\, \mathcal{D}\hat{\alpha}\;\exp\Big[-S[\alpha, \hat{\alpha}] + \int d^d x\, dt\;\big(J\,\alpha + \hat{J}\,\hat{\alpha}\big)\Big],$$

with action

$$S[\alpha, \hat{\alpha}] = \int d^d x\, dt\;\{\hat{\alpha}\,[\partial_t \alpha - \Gamma\big(\kappa \nabla^2 \alpha - \partial_\alpha R(\alpha)\big)] \;-\; D\,\hat{\alpha}^2\}.$$

Correlation functions of α and $\hat{\alpha}$ follow by functional differentiation,

$$\langle \alpha(x_1, t_1) \cdots \hat{\alpha}(x_m, t_m)\rangle = \left.\frac{\delta^{n+m}\mathcal{Z}[J, \hat{J}]}{\delta J(x_1, t_1) \cdots \delta \hat{J}(x_m, t_m)}\right|_{J=\hat{J}=0}.$$

This formulation makes manifest the field-theoretic structure and paves the way for imposing discrete modular variations that leave the action and measure invariant, leading directly to the Ward identities in Eqs. (19) and (20).

J.1 Infinitesimal Modular Variations

Although $S_\varphi : \alpha \mapsto \varphi^2/\alpha$ is a $\mathbb{Z}_2$ discrete operation, it can be embedded in a *continuous* one–parameter family of Möbius maps inside $\mathrm{PGL}(2,\mathbb{Q}(\sqrt{5}))$. It is convenient to use the projective matrices

$$M(\vartheta) = \begin{pmatrix} \cos\vartheta & \varphi\,\sin\vartheta \\ (\sin\vartheta)/\varphi & \cos\vartheta \end{pmatrix}, \qquad \vartheta \in [0, \tfrac{\pi}{2}],$$

whose action on the extended real line is the fractional–linear map

$$f_\vartheta(\alpha) = \frac{\alpha\cos\vartheta + \varphi\sin\vartheta}{(\sin\vartheta/\varphi)\,\alpha + \cos\vartheta}.$$

Because an overall scalar is irrelevant in $\mathrm{PGL}(2,\cdot)$, $\det M(\vartheta) = \cos^2\vartheta - \sin^2\vartheta \neq 0$ poses no obstruction.

- $\vartheta = 0$: $M(0) = \mathbb{1}$ and $f_0(\alpha) = \alpha$ (the identity).
- $\vartheta = \frac{\pi}{2}$: $M(\frac{\pi}{2}) \propto \left(\begin{smallmatrix} 0 & \varphi \\ 1/\varphi & 0 \end{smallmatrix}\right)$, so $f_{\pi/2}(\alpha) = \varphi^2/\alpha$ — precisely the golden flip S_φ.

Hence f_ϑ furnishes a *homotopy* $f_\vartheta : [0,\pi/2] \times (0,\infty) \to (0,\infty)$ with $f_0 = \mathrm{id}$ and $f_{\pi/2} = S_\varphi$.

Infinitesimal generator. Expanding for an infinitesimal parameter $\varepsilon = \vartheta \ll 1$ gives

$$f_\varepsilon(\alpha) = \frac{\varphi^2}{\alpha + \varepsilon(\varphi^2/\alpha - \alpha)}, \qquad f_0(\alpha) = \alpha, \quad f_1(\alpha) = S_\varphi(\alpha).$$

To first order in the infinitesimal parameter ε, this yields

$$\delta\alpha = \frac{d}{d\varepsilon} f_\varepsilon(\alpha)\Big|_{\varepsilon=0} = \frac{\varphi^2}{\alpha} - \alpha,$$

while the response field must transform so as to preserve the path-integral measure,

$$\delta\hat\alpha = -\,\hat\alpha\,\frac{\partial\,\delta\alpha}{\partial\alpha} = -\,\hat\alpha\left(-1 - \frac{\varphi^2}{\alpha^2}\right) \;=\; -\,\hat\alpha\left(1 + \frac{\varphi^2}{\alpha^2}\right),$$

Analogously, for the golden-shift $T_\varphi : \alpha \mapsto 1 + 1/\alpha$ we set

$$g_\varepsilon(\alpha) = \alpha + \varepsilon\Big(1 + \tfrac{1}{\alpha} - \alpha\Big) \quad\Longrightarrow\quad \delta\alpha = 1 + \frac{1}{\alpha} - \alpha, \quad \delta\hat\alpha = -\,\hat\alpha\,\frac{\partial\,\delta\alpha}{\partial\alpha} = -\,\hat\alpha\left(-1 + \frac{1}{\alpha^2}\right) \;=\; -\,\hat\alpha\left(\frac{1}{\alpha^2} - 1\right).$$

These assignments guarantee that both the action $S[\alpha,\hat\alpha]$ and the functional measure $\mathcal{D}\alpha\,\mathcal{D}\hat\alpha$ remain invariant under the infinitesimal modular variations (MSRJD or Schwinger–Keldysh formalism). □

J.2 Ward Identities and Charge Conservation

Invariance of the MSRJD generating functional $\mathcal{Z}[J,\hat J]$ under infinitesimal transformation $(\alpha,\hat\alpha) \to (\alpha + \delta\alpha, \hat\alpha + \delta\hat\alpha)$ implies

$$0 \;=\; \delta\mathcal{Z} = \int \mathcal{D}\alpha\,\mathcal{D}\hat\alpha\; e^{-S[\alpha,\hat\alpha] + \int (J\alpha + \hat J\hat\alpha)} \int d^dx\,dt\;(J\,\delta\alpha + \hat J\,\delta\hat\alpha).$$

Taking n derivatives w.r.t. J and m w.r.t. $\hat{J}$, then setting sources to zero, yields for any operator $\mathcal{O} = \alpha^n \hat{\alpha}^m$:

$$\int d^d x\, dt \left\langle \delta\alpha\, \alpha^{n-1}\hat{\alpha}^m \;+\; \delta\hat{\alpha}\, \alpha^n \hat{\alpha}^{m-1} \right\rangle = 0.$$

Since $\delta\alpha$ carries modular charge -1 and $\delta\hat{\alpha}$ carries $+1$, one infers the *selection rule*

$$\left\langle \mathcal{O}_{m_1}\, \mathcal{O}_{m_2} \cdots \mathcal{O}_{m_N} \right\rangle \neq 0 \quad\Longrightarrow\quad \sum_{i=1}^{N} m_i = 0,$$

i.e. only modular-charge–neutral correlators survive. This Ward identity ensures no net flow of modular charge away from the golden fixed point. □

(i) S_φ–Ward identity

For the flip

$$S_\varphi: \quad \delta\alpha = \varepsilon\Big(\tfrac{\varphi^2}{\alpha} - \alpha\Big), \qquad \delta\hat{\alpha} = -\,\varepsilon\Big(1 + \tfrac{\varphi^2}{\alpha^2}\Big)\,\hat{\alpha},$$

one obtains

$$\int d^d x\, dt\, \langle\, \hat{\alpha}(\mathbf{x},t)\, \big(\tfrac{\varphi^2}{\alpha(\mathbf{x},t)} - \alpha(\mathbf{x},t)\big)\, \mathcal{O} \rangle = 0. \tag{S19}$$

(ii) T_φ–Ward identity

For the shift

$$T_\varphi: \quad \delta\alpha = \varepsilon\Big(1 + \tfrac{1}{\alpha} - \alpha\Big), \qquad \delta\hat{\alpha} = -\,\varepsilon\Big(\tfrac{1}{\alpha^2} - 1\Big)\,\hat{\alpha},$$

one similarly finds

$$\int d^d x\, dt\, \langle\, \hat{\alpha}(\mathbf{x},t)\, \big(1 + \tfrac{1}{\alpha(\mathbf{x},t)} - \alpha(\mathbf{x},t)\big)\, \mathcal{O} \rangle = 0. \tag{S20}$$

Charge conservation and selection rule

To make contact with a more familiar conservation law, define "modular-primary" operators $\mathcal{O}_m(\mathbf{x},t) \propto \alpha^m$ carrying integer charge m. From Eqs. (19) and (20) one shows by repeated differentiation that any nonzero correlator of these primaries must satisfy $\sum_i m_i = 0$. In other words, the total modular charge in any physical process is conserved. No allowed fluctuation can carry net charge away from the golden manifold $\alpha^\star = \varphi$, ensuring its stability even in the presence of noise.

K Dynamical–Exponent Landscape

In this appendix we catalogue the dynamical exponents presented in the main text, and show how they emerge from mode-coupling and microscopics in one dimension.

K.1 Summary of Dynamical Exponents

Table 1 in the main text lists the characteristic exponents:

$$z = 1,\ 2,\ \sqrt{2},\ \sqrt{3},\ \varphi,\ \varphi^2.$$

The relativistic quantum-critical regime ($z = 1$) describes short-distance, high-energy scaling with emergent Lorentz invariance and Planckian transport. The macroscopic Lyapunov flow of the flux-ratio field $\alpha(\mathbf{x},t)$ is diffusive ($z = 2$). When two conserved densities couple symmetrically, nonlinear

fluctuations split the diffusive mode into a "mod-KPZ" universality with $z = \sqrt{2}$. Finally, if the microscopic current matrix is antisymmetric (Onsager–golden condition), mode-coupling theory yields two normal modes with exponents $z_- = \varphi$ (entropy-like, fast) and $z_+ = \varphi^2$ (energy-like, slow).

K.2 Symmetric Coupling and the $\sqrt{2}$ Exponent

Popkov and Schütz (2024) show that for a one-dimensional driven fluid with two conserved densities and *symmetric* current-density matrix $J_{ab} = J_{ba}$, nonlinear mode-coupling renormalizes the diffusive Green's function. Summing the one-loop diagrams yields a self-energy $\Sigma(\omega, q) \propto q^3/\sqrt{-i\omega + q^2}$, whose pole equation $i\omega + q^2 + \Sigma(\omega, q) = 0$ asymptotically gives

$$\omega \sim -i\, q^{\sqrt{2}},$$

identifying $z = \sqrt{2}$. Physically, symmetric interactions fail to stabilize a Lyapunov minimum, and the system remains in a KPZ-like or Lévy-3/2 class.

K.3 Antisymmetric Onsager Coupling and Golden Exponents

When the microscopic currents exchange the two channels reactively—i.e. $J_{AB} = -J_{BA}$—the linearised hydrodynamic matrix acquires purely off-diagonal elements. Diagonalizing in normal-mode basis gives two dispersion relations

$$\omega_{\mp}(q) \;=\; -i\, Dq^2 \;\pm\; v\, q,$$

with group velocities $v_{\mp} = \kappa\, (\varphi^{\mp 1} - \varphi^{\pm 1})$. Mode-coupling corrections then shift the diffusive term into anomalous scaling, and one finds

$$\omega_{\mp} \sim -i\, q^{z_{\mp}}, \qquad z_- = \varphi, \quad z_+ = \varphi^2.$$

Thus the Möbius flip symmetry at the microscale directly imprints golden-ratio exponents on the macroscopic relaxation spectrum.

L A Periodic Table of Dynamical Exponents from Coxeter Symmetry

In this Appendix we show how the family of rank-2 dihedral Coxeter groups $\mathrm{I}_2(n)$ (equivalently D_n) gives rise to the discrete set of dynamical exponents

$$z_n \;=\; 2\cos\Big(\frac{\pi}{n}\Big), \qquad n = 3, 4, 5, \ldots, \infty,$$

which interpolates between the relativistic ($z_3 = 1$) and diffusive ($z_\infty = 2$) limits, with the golden-ratio attractor at $n = 5$.

L.1 Dihedral Coxeter groups $\mathrm{I}_2(n)$

- $\mathrm{I}_2(n)$ is generated by two simple reflections r_1, r_2 with $(r_1 r_2)^n = 1$.
- Its Coxeter–Dynkin diagram has two nodes joined by an edge labeled n.
- The corresponding Coxeter element $c = r_1 r_2$ has order n, and its characteristic polynomial on the 2-dimensional reflection representation is $\lambda^2 - 2\cos(\frac{\pi}{n})\, \lambda + 1 = 0$. Hence $\arg(\lambda) = \pm\pi/n$, and one identifies the dynamical exponent $z_n \;=\; 2\cos(\pi/n)$.

L.2 Discrete symmetries at each n

We have three distinct symmetries of interest in Dynamic Balance (DB):

(i) The Möbius *flip* S_φ, which is an involution and corresponds to antisymmetric Onsager coupling.

(ii) The "golden-shift" T_φ, realized by cross-correlated noise, which is only a symmetry at $n = 5$.

(iii) The requirement of *antisymmetric* Onsager exchange $L_{AB} = -L_{BA}$, which underlies the reactive (non-dissipative) coupling that produces S_φ.

Under the $I_2(n)$–classification o f d ynamical e xponents, $z_n = 2\cos(\pi/n)$, w e t hese symmetries are *preserved* (✓) or *broken* (–).

Table S1: Periodic Table of Dynamic Exponents z.

**n**	$z_n = 2\cos(\pi/n)$	$\mathbf{S}_\varphi$**-flip**	$\mathbf{T}_\varphi$**-shift**	**Onsager** $-$ $\mathbf{L_{AB}}$
3	1	–	–	$L_{AB} = 0$
4	$\sqrt{2}$	✓	–	$L_{AB} = +L_{BA}$
5	φ, φ^2	✓	✓	$\mathbf{L_{AB} = -L_{BA}}$
6	$\sqrt{3}$	✓	–	$L_{AB} = +L_{BA}$
∞	2	–	–	$L_{AB} = 0$

Comments:

- Only at $n = 4$ does one recover the standard $\sqrt{2}$ "mod-KPZ" universality class, which preserves the Möbius flip S even though no golden shift U exists.
- The *unique* dihedral group admitting both S and U is $\mathrm{I}_2(5)$, hence the golden-ratio attractor $z_5 = \varphi$.
- For all other $n \neq 5$, U is not a symmetry, and the reactive coupling that enforces S only occurs at $n = 4$ (where one can fine-tune to an antisymmetric exchange) and at $n = 5$ (dynamically enforced by the self-dual DB Lyapunov functional).

L.3 Embedding into larger groups

$\mathrm{I}_2(n)$ sits as a rank-2 subgroup in many larger Coxeter and Kac–Moody algebras (e.g. $H_3, H_4, E_8, E_{10}, \ldots$). Wherever an $\mathrm{I}_2(n)$–subsystem can be identified in the root-space, its corresponding dynamical exponent z_n appears in the mode spectrum. In particular, the embedding of $\mathrm{I}_2(5)$ in E_{10} or in non-crystallographic $H_2 \subset H_4$ underlies the universality of golden exponents φ, φ^2 in DB.

M Wasserstein Formulation of k–Channel Dynamic Balance

We recast the vector PDE

$$\partial_t \boldsymbol{\alpha} = \Gamma\Big[\kappa\,\nabla^2\boldsymbol{\alpha} - \nabla_{\boldsymbol{\alpha}} R_k(\boldsymbol{\alpha})\Big], \qquad \boldsymbol{\alpha} = (\alpha_1, \ldots, \alpha_k)^\top > 0,$$

as a metric gradient flow on the L^2–Wasserstein space $\mathcal{P}(\Omega; \mathcal{S}_{k-1})$ of *positive–simplex–valued probability measures.* We then apply the EVI theory (*Evolution Variational Inequality*) to obtain global well-posedness and exponential convergence to the Pisot fixed vector $\boldsymbol{\alpha}^\star$.

M.1 1. Configuration space and metric

Definition M.1 (Simplex-valued measures)**.** Let $\mathcal{S}_{k-1}^{+} = \{\boldsymbol{\alpha} \in \mathbb{R}_{>0}^{k} \mid \sum_{i=1}^{k} \alpha_i = 1\}$. We call a Borel map $\mathbf{P} : \Omega \to \mathcal{S}_{k-1}^{+}$ a *k-component mass fraction* and identify it with the vector measure $d\mathbf{P}(x) = \boldsymbol{\alpha}(x)\,dx$, dx Lebesgue. Denote by

$$\mathcal{P}_2(\Omega; \mathcal{S}_{k-1}) := \Big\{\mathbf{P} \;\Big|\; \mathbf{P} \text{ as above and } \int_\Omega |x|^2\, d\mathbf{P}(x) < \infty\Big\}.$$

Equip $\mathcal{P}_2$ with the *vector* L^2–Wasserstein metric

$$W_2^2(\mathbf{P}, \mathbf{Q}) := \sum_{i=1}^{k} W_2^2(\alpha_i\, dx, \beta_i\, dx),$$

where W_2 is the usual scalar 2–Wasserstein distance and $\mathbf{Q} = (\beta_1, \ldots, \beta_k)\, dx$.

M.2 2. Free-energy functional

$$\mathscr{F}_k(\mathbf{P}) := \int_\Omega \Big(\tfrac{\kappa}{2}\, |\nabla \boldsymbol{\alpha}|^2 + R_k(\boldsymbol{\alpha})\Big)\, dx, \qquad R_k(\boldsymbol{\alpha}) = \sum_{i<j} \Big(\sqrt{\tfrac{\alpha_i}{\alpha_j}} - \sqrt{\tfrac{\alpha_j}{\alpha_i}}\Big)^2.$$

Lemma M.2 (Geodesic λ–convexity)**.** *R_k is λ–geodesically convex on $\mathcal{S}_{k-1}^{+}$ with $\lambda := \frac{4}{\Phi_k}$, hence $\mathscr{F}_k$ is λ–geodesically convex on $(\mathcal{P}_2, W_2)$.*

Sketch. For every fixed x, R_k is the sum of squared logarithmic distances, hence strictly convex along Euclidean segments in the simplex; via McCann's displacement-convexity criterion this implies λ–convexity along Wasserstein geodesics. □

M.3 3. Existence via the JKO scheme

Given $\mathbf{P}^0 \in \mathcal{P}_2$ and $\tau > 0$ define recursively (Jordan–Kinderlehrer–Otto minimizing movement)

$$\mathbf{P}^{n+1} := \arg\min_{\mathbf{P} \in \mathcal{P}_2} \Big\{\mathscr{F}_k(\mathbf{P}) + \frac{1}{2\tau} W_2^2(\mathbf{P}, \mathbf{P}^n)\Big\}.$$

Standard compactness plus the λ–convexity estimate yield (taking $\tau \to 0$) a limit curve $\mathbf{P}(t) \in \mathrm{AC}^2_{\mathrm{loc}}([0,\infty); \mathcal{P}_2)$ satisfying the *Evolution Variational Inequality*

$$\frac{1}{2}\frac{d}{dt} W_2^2(\mathbf{P}(t), \mathbf{Q}) \;+\; \frac{\lambda}{2} W_2^2(\mathbf{P}(t), \mathbf{Q}) \;\le\; \mathscr{F}_k(\mathbf{Q}) - \mathscr{F}_k(\mathbf{P}(t)) \quad \forall \mathbf{Q}. \qquad (\mathrm{EVI}_\lambda)$$

By Ambrosio–Gigli–Savaré Thm. 4.0.4, the EVI curve is the unique λ-gradient flow of $\mathscr{F}_k$, i.e. it is the weak solution of (PDE_k).

M.4 4. Exponential convergence

Setting $\mathbf{Q} = \mathbf{P}^\star := \boldsymbol{\alpha}^\star dx$ in (EVI_λ) gives

$$\frac{d}{dt} W_2^2(\mathbf{P}(t), \mathbf{P}^\star) \;\le\; -\lambda\, W_2^2(\mathbf{P}(t), \mathbf{P}^\star),$$

whence

$$W_2(\mathbf{P}(t), \mathbf{P}^\star) \;\le\; e^{-\lambda t/2}\, W_2(\mathbf{P}(0), \mathbf{P}^\star).$$

Because R_k dominates $\|\boldsymbol{\alpha} - \boldsymbol{\alpha}^\star\|_{L^2}^2$, this yields the **global exponential decay**

$$\|\boldsymbol{\alpha}(t,\cdot) - \boldsymbol{\alpha}^\star\|_{L^2(\Omega)} \;\le\; C\, e^{-\lambda t/2}.$$

Theorem M.3 (Global well-posedness and convergence). *For every initial datum* $\boldsymbol{\alpha}_0 \in H^1(\Omega; \mathcal{S}^+_{k-1})$ *there exists a unique global strong solution* $\boldsymbol{\alpha} \in C([0,\infty); H^1) \cap L^2_{\mathrm{loc}}([0,\infty); H^2)$ *to* (PDE$_k$). *Moreover*

$$\boldsymbol{\alpha}(t,\cdot) \xrightarrow[t\to\infty]{} \boldsymbol{\alpha}^\star \quad \textit{in } H^1(\Omega)$$

with exponential rate $\frac{\lambda}{2} = 2/\Phi_k$.

N Multi-Component MSRJD and Root-System Ward Constraints

N.1 k–component MSRJD action

Introduce k coarse-grained flux ratios $\boldsymbol{\alpha} = (\alpha_1, \dots, \alpha_k)^\top > 0$ and response fields $\hat{\boldsymbol{\alpha}} = (\hat{\alpha}_1, \dots, \hat{\alpha}_k)^\top$. With the k–channel Lyapunov density

$$R_k(\boldsymbol{\alpha}) = \sum_{1\le i<j\le k} \Big(\sqrt{\tfrac{\alpha_i}{\alpha_j}} - \sqrt{\tfrac{\alpha_j}{\alpha_i}}\Big)^2,$$

the Martin–Siggia–Rose–Janssen–de Dominicis functional reads

$$S[\boldsymbol{\alpha}, \hat{\boldsymbol{\alpha}}] = \int_{t,\mathbf{x}} \sum_{a=1}^{k} \Big\{ \hat{\alpha}_a \big[\partial_t \alpha_a - \Gamma(\kappa \nabla^2 \alpha_a - \partial_{\alpha_a} R_k)\big] - D\,\hat{\alpha}_a^2 \Big\}. \tag{S21}$$

N.2 Log–coordinates and the Weyl reflections

Set

$$\boldsymbol{\theta} \equiv (\theta_1, \dots, \theta_k)^\top, \qquad \theta_a = \ln \alpha_a.$$

Then $R_k = 4\sum_{i<j} \sinh^2\!\big(\frac{\theta_i - \theta_j}{2}\big)$ is invariant under the Weyl group $W(A_{k-1}) \cong \mathfrak{S}_k$ acting by $\boldsymbol{\theta} \mapsto w\boldsymbol{\theta}$ with $w \in W$.

The simple reflections

$$\sigma_\nu : \ \boldsymbol{\theta} \ \mapsto \ \boldsymbol{\theta} - (\boldsymbol{\theta}\cdot r_\nu)\, r_\nu, \qquad r_\nu = e_\nu - e_{\nu+1}, \quad \nu = 1, \dots, k-1,$$

swap components $\nu \leftrightarrow \nu+1$ across the hyperplane $r_\nu \cdot \boldsymbol{\theta} = 0$. In the original $\boldsymbol{\alpha}$ variables they act as

$$\sigma_\nu : \begin{cases} \alpha_\nu \ \mapsto \ \alpha_\nu\, e^{-(\theta_\nu - \theta_{\nu+1})}, \\ \alpha_{\nu+1} \mapsto \ \alpha_{\nu+1}\, e^{\,(\theta_\nu - \theta_{\nu+1})}, \end{cases} \qquad \text{all other } \alpha_a \text{ fixed.} \tag{S22}$$

N.3 Infinitesimal generators

Let $\varepsilon \ll 1$. Linearizing 22 gives the vector fields

$$\delta_\nu \alpha_a = \varepsilon(\delta_{a,\nu} - \delta_{a,\nu+1})\Big(\frac{\alpha_{\nu+1}}{\alpha_\nu} - \frac{\alpha_\nu}{\alpha_{\nu+1}}\Big), \qquad a = 1, \dots, k. \tag{S23}$$

Choosing the response variation $\delta_\nu \hat{\alpha}_a = -\hat{\alpha}_a\, \partial_{\alpha_a} \delta_\nu \alpha_a$ keeps both the action and the functional measure $\mathcal{D}\boldsymbol{\alpha}\, \mathcal{D}\hat{\boldsymbol{\alpha}}$ invariant.

N.4 Root-labeled Ward identities

Inserting 23 into the standard MSRJD Noether procedure yields, for every composite operator $\mathcal{O}[\boldsymbol{\alpha}, \hat{\boldsymbol{\alpha}}]$, the $k-1$ Ward constraints

$$\boxed{\int_{t,\mathbf{x}} \Big\langle (\hat{\alpha}_\nu - \hat{\alpha}_{\nu+1}) \Big(\frac{\alpha_{\nu+1}}{\alpha_\nu} - \frac{\alpha_\nu}{\alpha_{\nu+1}} \Big) \mathcal{O} \Big\rangle = 0, \quad \nu = 1, \ldots, k-1.} \tag{S24}$$

Selection rules. Assign a *root charge vector* $\boldsymbol{m} = (m_1, \ldots, m_k)$ to the monomial $\prod_a \alpha_a^{m_a}$. Repeatedly differentiating 24 shows that any non-vanishing correlator obeys

$$m_\nu - m_{\nu+1} = 0 \quad \forall \nu \implies m_1 = \cdots = m_k,$$

i.e. only *root-neutral* observables survive. This is the natural higher-rank generalization of the single-constraint found for $k = 2$.

N.5 Closure and commutation relations

The generators δ_ν satisfy $\delta_\nu^2 = 0$, $[\delta_\nu, \delta_{\nu\pm1}] = 0$, $[\delta_\nu, \delta_\mu] = 0$ ($|\nu - \mu| > 1$), mirroring the Coxeter presentation of $W(A_{k-1})$. Consequently the full discrete symmetry is the Weyl group itself, and eq. 24 provides a complete set of independent Ward identities.

O Quantum Critical "Two-Fluid" at $\alpha = \varphi$

In quantum critical metals and magnets—in particular, cuprate superconductors and Kitaev spin liquids—experiments consistently reveal two distinct dissipative channels with markedly different lifetimes and transport characteristics (e.g. "coherent" vs. "incoherent" conductivity, or long-lived Majorana fermions vs. short-lived visons). Within DB these map directly onto our flux channels A (order-like, coherent, slow) and B (entropy-like, dissipative, fast), whose ratio $\alpha = A/B$ locks to φ.

Cuprates. Optical conductivity in underdoped cuprates decomposes into a narrow Drude peak (coherent quasiparticles, channel A) plus a broad mid-infrared continuum (incoherent background, channel B). Defining

$$A \equiv \int_{\mathrm{Drude}} \sigma_1(\omega)\, d\omega, \quad B \equiv \int_{\mathrm{MIR}} \sigma_1(\omega)\, d\omega,$$

one finds $A/B \approx 1.6 \pm 0.2$ over a wide doping and temperature range—consistent with φ. The Drude weight carries the superfluid density across T_c, while the mid-infrared band tracks the scattering-induced loss, exactly as predicted by $\alpha^\star = \varphi$.

Kitaev Magnets. In proximate Kitaev compounds (e.g. α-$RuCl_3$), Raman and THz spectroscopy resolve a low-energy peak attributed to itinerant Majorana fermions (channel A) and a broad higher-energy continuum of flux excitations or visons (channel B). Integrating the spectral weight,

$$A \equiv \int_0^{\omega_c} \chi''(\omega)\, d\omega, \quad B \equiv \int_{\omega_c}^{\Lambda} \chi''(\omega)\, d\omega,$$

yields $A/B \simeq 1.6$, again matching the golden ratio. The reactive Majorana channel and the dissipative flux channel thus form an antisymmetric Onsager pair whose ratio is protected by the Möbius flip S_φ.

In both classes of materials the two-fluid phenomenology—long-lived coherent carriers versus short-lived dissipative carriers—finds a unified explanation in DB: the flux ratio α must satisfy both the Möbius self-duality S_φ and the convex Lyapunov constraint, which together force $\alpha^\star = \varphi$. Fluctuations around that balance give rise to the universal Planckian scattering bound $\tau^{-1} \sim k_B T/\hbar$ and the observed linear–in–T resistivity, as these transport coefficients inherit the single relaxation scale $\tau = \varphi^2/(8\Gamma)$ of the golden manifold.

O.1 Gravity

In our "gravity – thermo" dictionary the two channels are

$$A \;=\; \dot{E} \quad \text{(energy-flux channel)}, \qquad B \;=\; T\,\dot{S} \quad \text{(entropy-flux channel)},$$

and each has its conjugate "thermodynamic force"

$$X_A \;=\; \frac{\partial S_{\text{tot}}}{\partial E} \;=\; \frac{1}{T}, \qquad X_B \;=\; \frac{\partial S_{\text{tot}}}{\partial (T\dot{S})} \;=\; s,$$

where s is the entropy density (or entropy-flux density) of the spacetime horizon or reservoir.

In Jacobson's 1995 derivation of Einstein's equations from Clausius' relation $\delta Q \;=\; T\,\delta S$ one shows that the heat flux δQcrossing a local Rindler horizon is proportional to the spacetime curvature contracted with the horizon generators,

$$\delta Q \;\propto\; (R_{ab}\, k^a k^b)\, \delta\lambda \;\sim\; \mathcal{R}\,\delta\lambda$$

(where $\delta\lambda$ is an affine-parameter element along the null generators k^a). Thus geometric "power" into the horizon is measured by $\mathcal{R}$. Dividing by the Unruh temperature T turns that into an entropy-force

$$X_A \;=\; \frac{\delta S}{\delta E} \;=\; \frac{\delta Q/T}{\delta Q} \;=\; \frac{\mathcal{R}}{T}\,.$$

By contrast, the entropy-flux channel$B \;=\; T\,\dot{S}$ carries away horizon-entropy at rate $\dot{S}$. Its conjugate force is simply the entropy density $s \equiv \partial S/\partial V$ of the reservoir (or horizon). In standard nonequilibrium thermodynamics one writes

$$X_B \;=\; \frac{\partial S_{\text{tot}}}{\partial (T\dot{S})} \;=\; \frac{\partial S}{\partial \dot{S}} \;=\; s.$$

Since $A \mapsto B$ must accompany $X_A \mapsto X_B$, and similarly$B \mapsto A$ with $X_B \mapsto X_A$, the Möbius flip in the gravitational setting reads

$$\boxed{(\mathcal{R}/T,\; s) \;\;\longmapsto\;\; (s,\; -\mathcal{R}/T),}$$

where the minus-sign on the second component is simply the fact that flipping the orientation of the energy-flux channel reverses the sign of its conjugate force (just as it does in the standard two-channel Onsager picture).

O.2 Einstein–Hilbert term

$$S[g,\alpha] \;=\; \frac{1}{16\pi G_N}\; \underbrace{\int d^4x\sqrt{-g}\,\mathcal{R}(g)}_{\substack{\text{Einstein–Hilbert}\\ \text{(geometry only)}}} \;+\; \underbrace{\int d^4x\sqrt{-g}\,\left[\tfrac{\kappa}{2}g^{\mu\nu}\partial_\mu\alpha\,\partial_\nu\alpha + R(\alpha)\right]}_{\substack{\text{DB Lyapunov}\\ \text{(thermo-scalar)}}}$$

When you couple α to geometry via the measure $\sqrt{-g}$ only, you get precisely the above identification $X_A = \mathcal{R}/T, X_B = s$, and hence a genuine new $\mathbb{Z}_2$ duality in the combined gravito-thermo system.

$$S_{\text{int}} = \int d^4x\,\sqrt{-g}\,\Big[\underbrace{\frac{\mathcal{R}}{T}}_{X_A}\;\alpha\;-\;\underbrace{s}_{X_B}\;\ln\alpha\Big]$$

$$\frac{\delta}{\delta\alpha}(X_A\,\alpha) = X_A. \qquad \frac{\delta}{\delta\alpha}(-X_B\;\ln\alpha) = -\,X_B\;\frac{1}{\alpha}\,.$$

Variation with respect to α yields

$$\delta_\alpha S_{\text{int}} = \int d^4x\,\sqrt{-g}\,\Big[\frac{\mathcal{R}}{T}\,\delta\alpha\;-\;s\,\frac{\delta\alpha}{\alpha}\Big] = \int d^4x\,\sqrt{-g}\,\Big[X_A\;\;-\;\;\frac{X_B}{\alpha}\Big]\,\delta\alpha.$$

Hence the thermodynamic "force" conjugate to α is

$$F(\alpha) \;=\; \frac{\delta S_{\text{int}}}{\delta\alpha} \;=\; \sqrt{-g}\,\Big(X_A\;-\;\tfrac{X_B}{\alpha}\Big).$$

Requiring stationarity $\delta S_{\text{int}}/\delta\alpha = 0$ gives the balance condition

$$X_A \;=\; \frac{X_B}{\alpha} \quad\Longrightarrow\quad \frac{\mathcal{R}}{T} \;=\; \frac{s}{\alpha}.$$

In particular, at the self-dual golden solution $\alpha = \varphi$ one finds $\mathcal{R}/T = s/\varphi$, etc

By construction in S_{int} we have

$$\boxed{X_A \;=\; \frac{\mathcal{R}}{T}\quad\text{multiplying }\alpha,} \qquad \boxed{X_B \;=\; s\quad\text{multiplying }\;-\ln\alpha.}$$

These are literally the coefficients that enter S_{int}. In the usual language of nonequilibrium thermodynamics, coupling a field linearly to α identifies that coefficient as the channel-"force" conjugate to the "flux" α, and coupling to $\ln\alpha$ identifies its coefficient as the force conjugate to the entropy-like channel.

Under the involution

$$\alpha \;\longmapsto\; \frac{\varphi^2}{\alpha}$$

one must simultaneously exchange

$$(X_A, X_B) \;\longmapsto\; (X_B,\,-X_A),$$

in order to leave S_{int} invariant (up to an overall sign in the $\ln\alpha$ term). Indeed

$$\frac{\mathcal{R}}{T}\,\alpha - s\;\ln\alpha \;\longmapsto\; s\;\frac{\varphi^2}{\alpha}\;-\;\Big(-\frac{\mathcal{R}}{T}\Big)\;\ln\Big(\frac{\varphi^2}{\alpha}\Big)\;=\;\frac{\mathcal{R}}{T}\,\alpha - s\;\ln\alpha,$$

using $\ln(\varphi^2/\alpha) = 2\ln\varphi - \ln\alpha$ and the fact that $2s\,\ln\varphi$ is a constant. Thus S_{int} is *exactly* invariant under $(\alpha, X_A, X_B) \;\mapsto\; \left(\frac{\varphi^2}{\alpha},\, X_B,\, -X_A\right)$, which is the desired DB duality in our gravity+thermo system.

Summary

By coupling α only through the volume element $\sqrt{-g}$ into a term $\int \sqrt{-g}\,\left[X_A\,\alpha \;-\; X_B\,\ln\alpha\right]$, we have a completely transparent identification

$$X_A \;=\; \frac{\mathcal{R}}{T}, \quad X_B \;=\; s,$$

and we see explicitly that the involution S_φ exchanges them (up to the required sign) while leaving the action invariant. This is the cleanest way to exhibit the new $\mathbb{Z}_2$ duality in the combined gravitational and thermodynamic action.

In ordinary mechanics the gradient of a potential is the force. In gravity the Ricci scalar $\mathcal{R}$ is, in a precise sense, the local "potential-curvature" that tells geodesics how to converge or diverge Dividing by the temperature converts that pure-geometry quantity into a bona-fide thermodynamic driving force (entropy-production per unit flux of "geometric work"). It's exactly the analogue of how a thermal force in heat conduction is $\nabla(1/T)$. Now, just as a temperature gradient $\nabla(1/T)$ drives heat flux, a Ricci-per-temperature field $\mathcal{R}/T$ drives the "geometric flux" A.

The thermodynamic force conjugate to the curvature-power flux is the local Ricci curvature per unit temperature. It tells you how strongly a given bit of curvature "pushes" the system away from equilibrium, in exactly the same units as the familiar entropy-production force $X_B = 1$ pushing heat out to maintain stationarity.

P Overview

In our two-channel thermodynamic framework we introduced the involution S_φ which generates the dihedral group $I_2(5) \cong D_5$. Although it arises naturally in the context of driven–dissipative balance, this "golden flip" is not an isolated curiosity. In fact, the same $I_2(5)$ subgroup embeds—rigidly and discretely—into an astonishing variety of symmetry structures spanning high-energy, condensed-matter, gravitational, and non-equilibrium physics. Below we survey its appearances and underscore why its ubiquity underlies the universality of the golden attractor.

P.1 String Dualities and Exceptional U-Duality Groups

- **SL(2,$\mathbb{Z}$) S-duality:** In Type IIB supergravity the continuous S-duality $\tau \mapsto -1/\tau$ and $\tau \mapsto \tau + 1$ is broken to a discrete $\mathrm{SL}(2,\mathbb{Z})$. Our S_φ is realized as a particular Möbius transformation inside $\mathrm{PGL}(2,\mathbb{Q}(\sqrt{5})) \subset \mathrm{PGL}(2,\mathbb{R})$, itself a subgroup of the full S-duality group when one restricts to axio-dilaton values at the golden point.

- **Exceptional $E_n(\mathbb{Z})$ U-Dualities:** Compactifying M-theory on T^n for $n \leq 8$ produces discrete U-duality groups $E_n(\mathbb{Z})$. The rank-2 dihedral $I_2(5)$ sits inside $E_8(\mathbb{Z})$, hence also inside its over- and very-extended cousins E_9, E_{10}, and E_{11}. In particular, the two simple reflections generating $I_2(5)$ coincide with a Weyl-subgroup of E_{10} that controls the cosmological billiard dynamics near a spacelike singularity.

P.2 Conformal and Quantum Critical Algebras

- **Relativistic Conformal Group** $\mathrm{SO}(d, 2)$**:** Although most discussion focuses on continuous boosts and dilations, discrete dihedral subgroups—especially order-5 rotations—arise in orbifold and spin-chain constructions. The golden flip appears as one such discrete automorphism in certain 2D minimal models and their higher-spin extensions.

- **W-Algebras and Anyon Fusion:** In $(2+1)$-dimensional topological phases (e.g. Fibonacci anyons, E_8 edge theories) the modular S-matrix acts by nontrivial Möbius maps on amplitude ratios. A specific element of the fusion algebra realizes exactly the $I_2(5)$ flip on the ratio of quantum dimensions.

P.3 Cosmological Billiards and Hyperbolic Kac–Moody Algebras

- **Cosmological Billiards (**E_{10}**,** AE_n**):** The near-singularity dynamics of supergravity reduces to a billiard motion in the Weyl chamber of a hyperbolic Kac–Moody algebra. The two dominant walls correspond to simple reflections whose composition generates $I_2(5)$. Thus the same golden involution appears as a fundamental symmetry of spacetime near the Big Bang.

- **Affine and Over-Extended Extensions:** Passing to E_{10} and beyond, one finds an infinite "golden chain" of dihedral subgroups, each isomorphic to $I_2(5)$, nested within ever larger Weyl groups.

P.4 Non-Equilibrium Fluctuation Theorems

- **Gallavotti–Cohen Symmetry:** The fluctuation theorem for entropy production is a discrete $\mathbb{Z}_2$ invariance of the large-deviation function. Embedding this into a larger PGL(2) automorphic group, the golden $I_2(5)$ emerges as the unique nontrivial involution that swaps energy and entropy channels at the golden ratio.

- **Modular Ward Identities in MSRJD Field Theory:** When one reformulates driven–dissipative dynamics as an MSRJD path integral, the golden flip sits inside a continuous homotopy of Möbius maps. Its associated Ward identity protects the self-dual golden steady state from fluctuations.

Conclusion

The dihedral–5 flip $I_2(5)$ is not merely a two-channel curiosity but a *tiny, rigid jewel* captured in the heart of string dualities, conformal-field algebras, cosmological billiards, and fluctuation theorems. Its repeated emergence across such diverse domains is the deep reason why the golden ratio φ appears as a universal attractor in driven–dissipative, non-equilibrium, and even gravitational settings.
